# Microbial Community Dynamics during Biodegradation of Crude Oil and Its Response to Biostimulation in Svalbard Seawater at Low Temperature

**DOI:** 10.3390/microorganisms9122425

**Published:** 2021-11-24

**Authors:** Hiie Nõlvak, Nga Phuong Dang, Marika Truu, Angela Peeb, Kertu Tiirik, Megan O’Sadnick, Jaak Truu

**Affiliations:** 1 Institute of Molecular and Cell Biology, University of Tartu, 51010 Tartu, Estonia; marika.truu@ut.ee (M.T.); angela.peeb@ut.ee (A.P.); kertu.tiirik@ut.ee (K.T.); jaak.truu@ut.ee (J.T.); 2 Department of Cold Climate Technology, SINTEF Narvik AS, N-8504 Narvik, Norway; nga.dang@sintef.no (N.P.D.); megan.osadnick@sintef.no (M.O.)

**Keywords:** arctic seawater, crude oil, hydrocarbon degradation potential, biostimulation, taxonomic classification methodology, oil hydrocarbon-degrading microbial taxa

## Abstract

The development of oil exploration activities and an increase in shipping in Arctic areas have increased the risk of oil spills in this cold marine environment. The objective of this experimental study was to assess the effect of biostimulation on microbial community abundance, structure, dynamics, and metabolic potential for oil hydrocarbon degradation in oil-contaminated Arctic seawater. The combination of amplicon-based and shotgun sequencing, together with the integration of genome-resolved metagenomics and omics data, was applied to assess microbial community structure and metabolic properties in naphthenic crude oil-amended microcosms. The comparison of estimates for oil-degrading microbial taxa obtained with different sequencing and taxonomic assignment methods showed substantial discrepancies between applied methods. Consequently, the data acquired with different methods was integrated for the analysis of microbial community structure, and amended with quantitative PCR, producing a more objective description of microbial community dynamics and evaluation of the effect of biostimulation on particular microbial taxa. Implementing biostimulation of the seawater microbial community with the addition of nutrients resulted in substantially elevated prokaryotic community abundance (103-fold), a distinctly different bacterial community structure from that in the initial seawater, 1.3-fold elevation in the normalized abundance of hydrocarbon degradation genes, and 12% enhancement of crude oil biodegradation. The bacterial communities in biostimulated microcosms after four months of incubation were dominated by Gammaproteobacterial genera *Pseudomonas*, *Marinomonas*, and *Oleispira*, which were succeeded by *Cycloclasticus* and *Paraperlucidibaca* after eight months of incubation. The majority of 195 compiled good-quality metagenome-assembled genomes (MAGs) exhibited diverse hydrocarbon degradation gene profiles. The results reveal that biostimulation with nutrients promotes naphthenic oil degradation in Arctic seawater, but this strategy alone might not be sufficient to effectively achieve bioremediation goals within a reasonable timeframe.

## 1. Introduction

Marine ecosystems are exposed to petroleum hydrocarbons through a variety of natural mechanisms, such as natural seeps, and anthropogenic activities, such as accidental oil spills during oil production and transport. Climate change has elevated the risk of crude oil spillage, especially in cold Arctic marine environments, where the severely reduced sea-ice-covered area has enabled the growth of oil exploration activity as well as prolonged and more frequent usage of Arctic shipping routes [1]. In Arctic regions, the complete clean-up of oil spills using traditional methods such as skimmers or beams is often challenging due to remote or poorly accessible locations and extreme weather conditions [2]. Consequently, bioremediation techniques, which rely on the potential of indigenous seawater microbial communities to degrade oil hydrocarbons, have been suggested as suitable and labor-effective clean-up methods in remote Arctic locations. An understanding of seawater microbial community structure and its potential for the degradation of different oil compounds is essential for determining the framework of a possible bioremediation approach.

Crude oils are extremely complex mixtures of aliphatic and aromatic hydrocarbons, asphaltenes, and resins, which have been part of the marine environment for millions of years due to natural seepage. Owing to this exposure, marine microbial communities possess remarkable potential and a diverse range of pathways for degrading these compounds [3]. Overall, more than 350 prokaryotic genera have been shown to degrade hydrocarbons (Supplementary File S1), including a variety of marine psychrophilic and psychrotolerant bacterial genera, such as *Colwellia*, *Cycloclasticus*, *Loktanella*, *Marinomonas*, *Pseudomonas*, and *Sulfitobacter* [4], most of which belong to the phyla Proteobacteria (especially classes Alpha-, Beta-, and Gammaproteobacteria) and Bacteroidetes.

Among several bioremediation methods, biostimulation with the addition of nutrients or the use of dispersants to increase the oil degradation rate of indigenous microbes, as relatively easily implemented and cost-effective clean-up actions, have been suggested to be combined with other clean-up technologies in seawater [4]. In cold seawater, microbial growth and consequently its oil degradation rate can be limited by the low availability of nutrients such as nitrogen and phosphorus [3]. Biostimulation with nutrient addition helps to compensate for this deficiency and to enhance oil degradation by allowing excess microbial growth, while the addition of dispersants makes oil more accessible to microbes by breaking it into small droplets with a higher surface-to-volume ratio [4]. The accelerated oil degradation rate in warm seawater in response to nutrient addition has been shown in microcosm and mesocosm studies [5,6,7,8]. In Arctic seawater, the effect of biostimulation and dispersant use on the crude oil biodegradation rate has been addressed in several studies [9,10,11]. However, previous research has overlooked the potential of biostimulation with nutrient addition in oil-contaminated Arctic seawater.

In recent years, the rapid development of microbial analysis tools, especially whole DNA metagenomics-based approaches enabling simultaneous retrieval of both taxonomic and functional information, has provided unprecedented amounts of information for use in bioremediation strategy development [12]. Knowledge of the diversity and structure of microbial communities in oil-contaminated Arctic seawater has expanded with several amplicon- or metagenome-based community sequencing studies using different taxonomic classification methods and reference databases [10,11,13,14,15]. The determination of microbiome structure and diversity is based on the assignment of individual reads to taxa by comparing them to reference databases [12], but a “gold standard” method has yet to be established. Hence, for a more realistic assessment of oil biodegradation potential and the realization of bioremediation approaches, information on the possible occurrence and nature of technical biases introduced to the diversity and structure estimates of microbial communities in Arctic seawater by widely used classifiers is needed.

The main objective of this study was to assess the effect of biostimulation on the dynamics of microbial community abundance, structure, and metabolic potential for oil hydrocarbon degradation in oil-contaminated Arctic seawater. For this purpose, a combination of amplicon-based and shotgun sequencing with several different taxonomic assignment methods, together with genome-resolved metagenomics and omics data integration, was applied in an oil-amended microcosm experiment.

## 2. Materials and Methods

### 2.1. Experimental Setup and Sampling

In order to evaluate the petroleum hydrocarbon degradation potential of the microbial community of Arctic seawater, an eight-month-long microcosm experiment was conducted in the lab of the Northern Research Institute in Narvik (Norway). The surface seawater (100 L total) was collected from three locations (78°14′31.4″ N, 14°44′20.1″ E; 78°13′35.6″ N, 14°10′18.3″ E; and 78°09′21.3″ N, 14°03′17.2″ E, respectively) at Svalbard in April 2016 using a sterilized bucket and stored at 4 °C in sterilized 40 L containers during transportation to the lab. Equal volumes of seawater from different locations were pooled, and the composite seawater was used to establish the microcosms immediately after arrival in the lab. Troll B type (North Sea naphthenic) crude oil of 2013 (provided by Statoil, Mongstad, Norway), previously pasteurized by heating at 70 °C for 30 min for three consecutive days, was used in this experiment.

Twelve 1-L bottles (DWK Life Sciences, Mainz, Germany) covered with a cotton stopper and filled with 900 mL of pooled seawater were used for the microbial community analysis. For oil degradation treatments (SWO), four bottles were supplemented with 9 g of crude oil. To evaluate the effect of biostimulation on oil degradation (SWOB), four bottles were supplemented with 9 g of oil and 4.5 g of Full fertilizer^®^ 22-3-10 (Yara, Glomfjord, Norway) (final concentration of NO_3_^−^ 500 mg/L and NH_4_^+^ 580 mg/L; P 130 mg/L; K 430 mg/L). Four seawater bottles without any additives (SW) served as controls. The microcosms were incubated at 4 °C in the dark without agitation. After four months of incubation, two bottles of each treatment were collected for microbiological analysis. Water from the two remaining bottles for both treatments was collected after eight months of incubation. In the text, samples taken at different sampling times are differentiated by the respective numbers in the abbreviations of the treatments.

In parallel, twelve 200 mL microcosms (four replicates of sterilized seawater with 2 g of oil (SWO_S_), four replicates of seawater with 2 g of crude oil (SWO), and four replicates of biostimulated (1 g of NPK fertilizer) seawater with 2 g of crude oil (SWOB)) were incubated under similar conditions and were used to assess the amount of remaining oil in the seawater. Two replicates (entire microcosms) of each treatment were sampled after four months, and the two remaining parallel replicates were sampled after eight months of incubation.

### 2.2. Chemical Analyses

Concentrations of nitrate-nitrogen (NO_3_-N), ammonium-nitrogen (NH_4_-N), and total phosphorus (P_tot_) in the initial pooled seawater and microcosms at the time of sampling were determined spectrometrically using a nitrate test in seawater, ammonium cell test, and phosphate cell test, respectively (Merck Millipore, Darmstadt, Germany) following the manufacturer’s instructions. Total nitrogen (N_tot_) and total organic carbon (TOC) concentrations were analyzed by Akvaplan Niva, Tromsø, Norway. The pH was determined using pH/Cond 340i meter (WTW GmbH, Weilheim, Germany), the salinity was measured with a YSI 30-25FT salinity meter (YSI Inc., Yellow Springs, OH, USA), and dissolved oxygen content was determined with an FDO^®^925 optical oxygen sensor (WTW GmbH). The values of the measured physicochemical parameters are given in Table S1.

For the aliphatic hydrocarbon and total hydrocarbon content (THC) analysis, hydrocarbons were extracted from water with dichloromethane (DCM, 1:3 *v*/*v*). The DCM mixture was then concentrated by evaporation. The concentrated extract was purified by solid-phase extraction through a silica column and further evaporated before gas chromatography analysis. The aliphatic hydrocarbons and THC of the samples were determined by GC-FID analysis using an Agilent 7890A model gas chromatograph equipped with a flame ionization detector (Agilent Technologies, Inc., Santa Clara, CA, USA) and Agilent CP-Sil 8 CB Fused Silica capillary columns (25 m × 0.32 mm ID, 0.25 µm film thickness) (Agilent Technologies, Inc.). Hydrogen was used as the carrier gas (flow 2.5 mL/min), and the injection volume was 1 µL in splitless mode. The following temperature program was used: 55 °C for 3 min, ramping 25 °C/min to 300 °C for 10 min.

The analysis of 16 priority polycyclic aromatic hydrocarbons (PAHs) was performed on a gas chromatography-mass spectroscopy (GC-MS) system (Agilent 7890A GC, Agilent Technologies, Inc.) coupled with Agilent 5975C MSD (Agilent Technologies, Inc.) and equipped with an Agilent HP-5ms Ultra Inert Fused Silica capillary column (30 m × 0.25 mm ID, 0.25 µm film thickness) (Agilent Technologies, Inc.). Helium was used as a carrier gas at a constant pressure of 14.093 psi, and the injection volume was 1 µL in splitless mode. The following temperature program was used: 50 °C for 2 min, ramping 30 °C/min to 100 °C, 6 °C/min to 230 °C for 2 min, 20 °C/min to 280 °C for 5 min, and 20 °C/min to 310 °C for 10 min. Temperatures of the MSD transfer line, MS ion source, and MS quadrupole were maintained at 310 °C, 230 °C, and 150 °C, respectively.

### 2.3. DNA Extraction

Two liters of the initial pooled seawater (SW0) and the entirety of collected microcosm samples (0.9 L) were filtered aseptically through 0.2 μm Sterivex SVGPL10RC filters (Merck Millipore). After the filtration, the filters were stored at −80 °C until DNA extraction. The DNA was extracted using DNeasy PowerWater Sterivex Kit (Qiagen, Foster City, CA, USA) according to the protocol of the manufacturer. The quantity and quality of DNA extracts were determined by spectrophotometry (Infinite M200, TecanAG, Grödig, Austria). The extracted DNA was stored at −20 °C prior to further analysis.

### 2.4. Quantitative PCR Conditions and Data Analysis

Quantitative PCR (qPCR) was used to determine the abundances of 16S rRNA genes specific to bacteria (B16S), archaea (A16S), *Colwellia*, *Cycloclasticus*, and *Pseudomonas* genera. The qPCR assays were performed on RotorGene^®^ Q with RotorGene Series Software v 2.0.2 (Qiagen). Stock solutions of target sequence-containing plasmids (Eurofins MWG Operon, Ebersberg, Germany) were used to create serially diluted standard curves, ranging from 25 to 10^8^ copies of each target gene. The qPCR reactions were performed in 10 µL volume containing 5 µL of Maxima SYBR Green Master Mix (Thermo Fisher Scientific Inc., Waltham, MA, USA), an optimized concentration of forward and reverse primers (Table S2), 1 µL of template DNA, and sterile distilled water. The used primers and optimized qPCR programs are described in Table S2. All qPCR samples were measured in triplicate, and negative controls were included in every qPCR run.

Quantification data were analyzed with the LinRegPCR program v 2020.0 [16]. The target gene abundance was calculated through the estimation of the fold difference between a sample and multiple data points from the standard curve, as described previously [17], and is presented as gene copy numbers per mL of analyzed water. The total 16S rRNA gene abundance (16S_tot_) was calculated by summing the bacterial and archaeal 16S rRNA gene abundances. The relative abundances (%) of archaea in the prokaryotic community and targeted genera in the bacterial community were also calculated.

### 2.5. Database of Genera Containing Oil Hydrocarbon-Degrading Organisms

A list of bacterial (*n* = 350) and archaeal genera (*n* = 14) containing oil hydrocarbon-degrading organisms (HDOs) was compiled (Supplementary File S1) based on reports in the literature (up to October 2021). An organism was considered an oil hydrocarbon degrader either when it was directly shown to degrade oil hydrocarbon compounds or when it possessed genes related directly to oil hydrocarbon degradation pathways.

### 2.6. Taxonomic Profiling of Prokaryotic Community

The prokaryotic community structure and composition were assessed using amplicon-based and whole-genome shotgun sequencing analysis. The sequence data of both methods are accessible in online repositories; the names of the repositories and accession numbers can be found at https://ebi.ac.uk/ena, PRJEB48192.

#### 2.6.1. Shotgun Metagenomic Sequencing

Due to low DNA concentrations in extracts, for the preparation of DNA libraries for the metagenomic analysis, the DNA from two parallel microcosms of the same treatment was pooled to fulfill the technical requirements of the method. The pooled DNA samples were purified and concentrated with NucleoSpin^®^ Gel and PCR Clean-up kit (MACHEREY-NAGEL GmbH & Co. KG, Düren, Germany) according to the manufacturer’s protocol. Paired-end sequencing libraries (2 × 150 bp) were constructed using the Nextera XT DNA Library Preparation kit (Illumina, San Diego, CA, USA) according to the manufacturer’s instructions and sequenced using the NovaSeq 6000 system (Illumina).

The quality of obtained raw metagenomic sequences was controlled using FastQC v 0.11.7 [18]. Reads <35 bp and poly-G tails were removed and bases with quality scores lower than 20 were trimmed with Cutadapt v 1.16 [19]. Coverage and diversity metrics of the quality-controlled metagenomic sequences were estimated using Nonpareil v 3.3.3 [20]. The characteristics of the metagenomic data are presented in Table S3.

Bacterial and archaeal communities were classified to species level using Kaiju v 1.7.3 [21] with the NCBI-nr database. Contigs were assembled using Megahit v 1.2.9 [22]. The minimum length of contigs was 1000 bp. Contigs were assembled into metagenome-assembled genomes (MAGs) with MetaBAT2 v 2.15 [23], followed by quality assessment with CheckM v 1.0.18 [24] and taxonomic assessment with Kaiju v 1.7.3 with the NCBI-nr database. dRep v 2.6.2 [25] was used to assess the relatedness of genomes, and MAGs with average nucleotide identity (ANI) score ≥95% were considered to belong to the same organism. Both contigs and MAGs were analyzed for hydrocarbon degradation-related gene (HDG, *n* = 92) annotations with HMMER v 3.3 using HMM models from the KEGG KO database (*n* = 91) [26]. For the long-chain alkane degradation-related *almA* gene, which is missing from the KEGG KO database, a custom HMM profile was built with HMMER v 3.3 using pairwise alignment results of *almA* sequences [27]. The values of HDGs of prokaryotic communities were calculated using MicrobeCensus v 1.1.1 [28] and are presented as gene-specific reads per kilobase per genome equivalent (RPKG). HDGs were regarded as present in a MAG when >50% of gene cluster components were registered.

#### 2.6.2. Amplicon-Based Sequencing

The universal primers 515F (5′-GTGYCAGCMGCCGCGGTAA-3′) and 926R (5′-CCGYCAATTYMTTTRAGTTT-3′) [29] were used for targeting the V3-V5 hypervariable region of both the bacterial and archaeal 16S rRNA genes in each microcosm. The PCR reaction mixture for amplification of each sample contained a unique combination of primers; each primer had a specific 6 bp long barcode sequence at the 5′ end [30]. All PCR reactions were performed in a 20 μL reaction mixture using Phusion Hot Start High-Fidelity Polymerase (Thermo Fisher Scientific Inc.) according to the manufacturer’s instructions and 0.4 μM primers at the following amplification conditions: 98 °C for 30 s; 25 cycles: 98 °C for 10 s; 60 °C for 30 s; 72 °C for 15 s; 72 °C for 8 min. The amplification of each sample was performed in triplicate. The replicate PCR products were pooled, and the concentration of each composite sample was determined with the TapeStation 2200 using D1000 ScreenTapes^®^ (Agilent Technologies, Inc.). Amplicons of all samples were finally pooled in equal proportions, and the mixture was purified and concentrated using the NucleoSpin^®^ Gel and PCR Clean-up kit (MACHEREY-NAGEL GmbH&Co. KG). The paired-end DNA library was prepared according to Herbold and co-authors [31] and sequenced on an Illumina^®^ MiSeq system (Illumina) at Microsynth AG (Balgach, Switzerland).

The paired-end reads were assembled into composite reads with Pear v 0.9.11 [32]. Bacterial and archaeal sequences were separated by adapter tags using BBMap v 37.86. The assembled reads were processed using Mothur v 1.40.4 [33]. Illumina reads were demultiplexed, and during data denoising, sequences were discarded if the average sequencing quality score dropped <25 over a 25-bp sliding window, were <100 bp, had any ambiguous bases, and had longer than 6 homopolymers. Chimeras were detected with the vsearch algorithm in de novo mode [34]. Quality-checked reads were clustered into operational taxonomic units (OTUs) using a distance-based greedy clustering method with a 97% similarity threshold [35]. Spurious OTUs with less than 3 sequences were discarded. The characteristics of amplicon-based sequencing data are given in Table S4.

#### 2.6.3. Comparison and Integration of Bacterial Community Taxonomic Classification Methods

In order to evaluate whether the choice of sequencing and taxonomic affiliation method influences the estimation of bacterial community structure and particularly the estimates for the oil-degrading taxa, five different taxonomic classification methods were applied for a comparative analysis of initial seawater and treatments after four and eight months of incubation. Metagenomic data were classified to the genus level using Kaiju [21] with the NCBI-nr database, Kaiju with MAR*_DB_* [36] databases, and Kraken2 v 2.1.1 [37] as well as Bracken v 2.6.1 [38] with the Standard Kraken2 database. Programs were run with default parameters using paired-end reads. The amplicon-based sequencing data were classified using SILVA alignment v 132 [39]. Sequences were classified using 80% confidence in bootstrap values. Eukaryotes and reads that were unable to be taxonomically assigned were removed from the subsequent analysis. These classification methods are referred to as Kaiju, Kaiju/MAR, Kraken2, Bracken, and Amplicon, respectively, in the further text and in figures.

The top 20 bacterial phyla (Proteobacteria were characterized at the class level) of each sample analyzed with all five taxonomic classification methods were ranked in descending order, assigning the highest rank value to the phylum with the highest proportion in the bacterial community. An average rank estimate for each phylum based on its ranks in five taxonomic classifications was also calculated. The proportions of bacterial genera containing HDOs were derived from taxonomic classification data according to an HDO database compiled in this study (Supplementary File S1) and used with the 16S_tot_ values of each sample to calculate the estimated abundances of potential hydrocarbon degraders. The overlap of the top 50 bacterial genera derived with five different classification methods as well as the presence of genera containing HDOs was visualized using the online Venn diagram tool: http://bioinformatics.psb.ugent.be/webtools/Venn/ (accessed on 10 September 2021). The clustering of samples based on the crl-transformed proportions of the top 50 genera in the bacterial community according to each classification method was performed using Euclidean distance and the Ward-linkage method, and analysis results were visualized as heatmaps using the ClustVis program [40].

The proportions of both the top 50 genera and genera containing HDOs in the bacterial community according to the five taxonomic classification methods were integrated using multiple co-inertia analysis (MCIA) with the package omicade4 in R v. 4.0.3 [41].

## 3. Results

### 3.1. Oil Hydrocarbon Depletion

**Figure 1:**
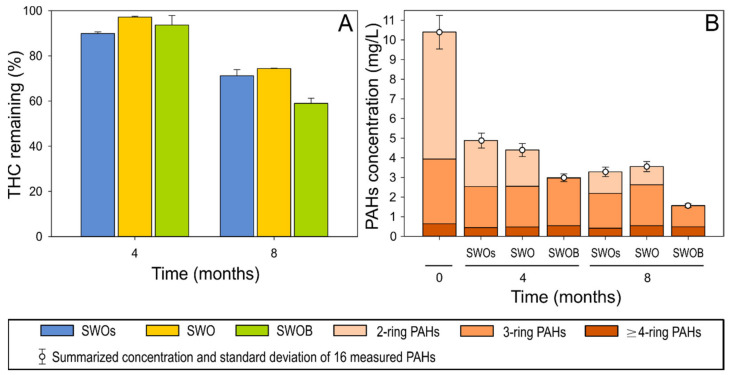
The proportion of total hydrocarbons (THC) remaining (**A**) and the concentration of 16 priority PAHs (**B**) in the oil-amended sterilized seawater (SWOs), oil-contaminated seawater (SWO), and biostimulated oil-contaminated seawater (SWOB) at 4 and 8 months. *N* = 2.

The depletion of THC was ≤10% in all treatment variants during the first four months of the experiment (Figure 1A). By the eighth month, 29% of THC was depleted in the sterilized treatment (SWOs) variant. Biodegradation in the non-biostimulated treatment (SWO) did not appear to substantially contribute to either THC or aliphatic hydrocarbon depletion. However, biodegradation in biostimulated (SWOB) microcosms added another 12% (total depletion of 41%) to THC depletion. The decreasing ratios of *n*-C_17_pPristane and *n*-C_18_/phytane also indicated the occurrence of biodegradation only in the SWOB treatment (Table S5).

The quantitative analysis of 16 priority PAHs indicated that the highest PAH depletion (71% and 85% at four and eight months, respectively) occurred in the SWOB treatment, while the depletion rates were relatively similar (53–58% and 65–68% after four and eight months, respectively) in SWOs and SWO (Figure 1B). After four months, almost complete depletion (99.5%) of naphthalene, representing two-ring PAHs, was detected in the SWOB variant (Table S5), while the depletion of three-ring PAHs in this variant was less than that in other treatments. However, by the eighth month, the depletion rate (66%) of three-ring PAHs (mainly fluorene and phenanthrene) in SWOB also considerably exceeded (by 21–30%) the rate in other treatments. The removal of ≥4-ring PAHs was in the range of 30–40% in all treatments.

### 3.2. Microbial Community Abundance and Composition

#### 3.2.1. Microbial Community Abundance

**Figure 2:**
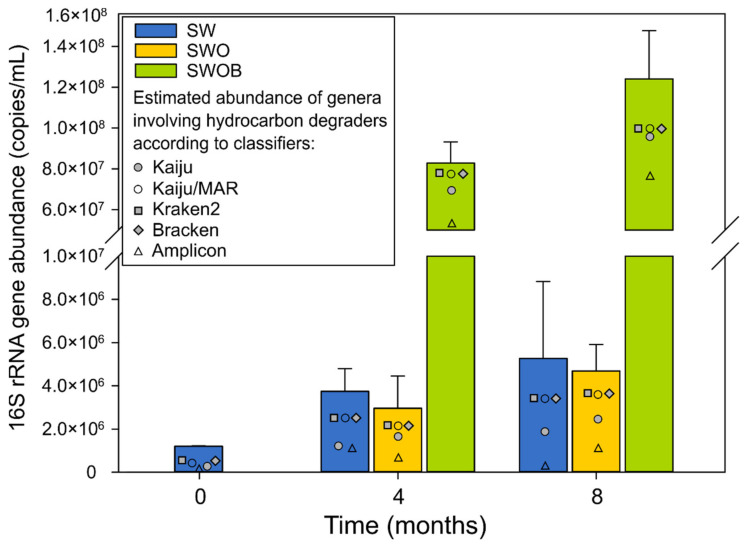
The average abundances and SD (*n* = 2) of summarized bacterial and archaeal 16S rRNA genes as well as estimated abundances of genera containing oil hydrocarbon degraders based on taxonomic assignment using Kaiju with NCBI-nr and MAR*_DB_* databases, Kraken2 and Bracken with Standard Kraken2 database, and amplicon-sequencing data with SILVA database in seawater (SW), oil-contaminated seawater (SWO) and biostimulated oil-contaminated seawater (SWOB) after 0, 4, and 8 months of incubation.

The average abundance of 16S_tot_ in microcosms on day 0 was 1.2 × 10^6^ copies/mL (Figure 2), with B16S accounting for 84.5% of the community. While the 16S_tot_ abundance underwent moderate growth in SW and SWO throughout the experiment (2.5–4.4-fold), the biostimulation (SWOB) approach markedly enhanced the total prokaryotic (69- and 103-fold increase at four and eight months, respectively), and especially the bacterial (Figure S1A; Table S6), community growth.

A16S abundance was on average 1.8 × 10^5^ copies/mL at the start of the experiment, which accounted for 15.5% of the microbial community in the initial seawater (Figure S1B,C). A16S abundance dropped 5.3–9.5-fold in SW and SWOB microcosms during the experiment. Archaea were more resilient in the SWO treatment, where the A16S abundance was decreased by 3.3- and 1.8-fold after four and eight months of incubation, respectively, compared to the start of the experiment. By the fourth month, the relative abundances of archaea had reached levels of 0.5% in SW, 2.1% in SWO, and 0.03% in SWOB treatment, which were maintained until the end of the experiment (Figure S1C).

#### 3.2.2. Microbial Community Structure

##### Estimation of Bacterial Community Structure According to Different Classification Methods

Since bacteria formed most of the prokaryotic community in the microcosms, further analysis focused on the bacterial community with classification using different methods yielding inconsistent results: 67–92% and 71–87% of total reads were classified using Kaiju and Kaiju/MAR approaches, respectively, while only 18–62% of reads could be classified when using Kraken and Bracken (Table S3).

**Figure 3:**
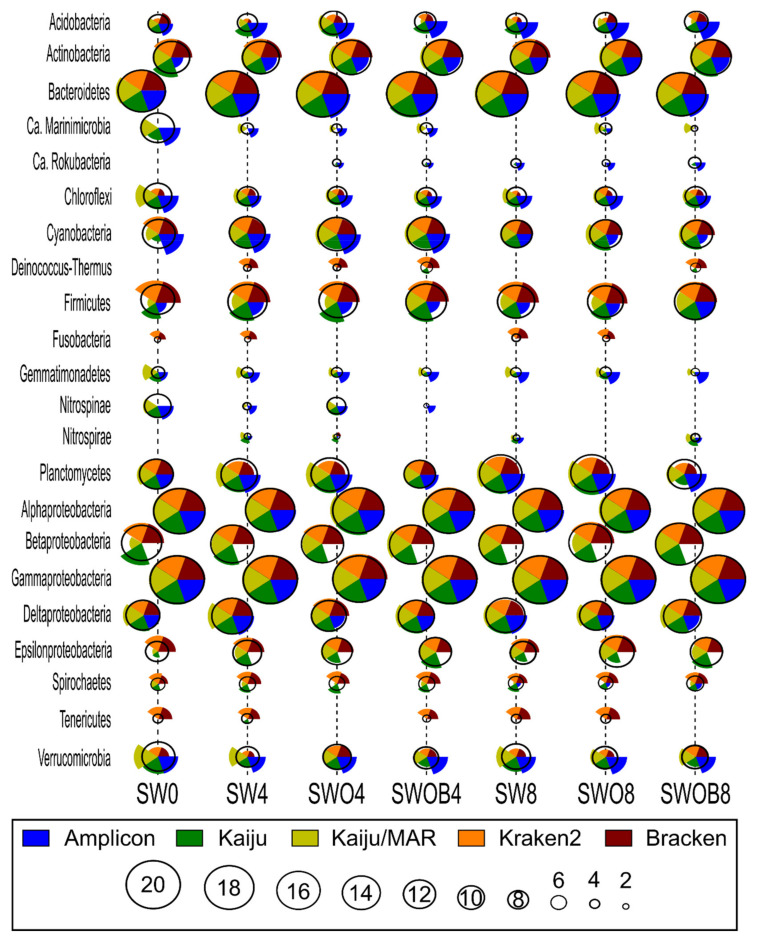
The comparison of bacterial community structure at the phylum level (Proteobacteria at class level) by ranks of the 20 most prominent taxa of each sample (highest rank value corresponds to highest proportion in bacterial community) by different taxonomic classification methodologies (Kaiju with NCBI-nr and MAR*_DB_* databases, Kraken2 and Bracken with Standard Kraken2 database, and amplicon-based data with SILVA database) in seawater (SW), oil-contaminated seawater (SWO), and biostimulated oil-contaminated seawater (SWOB). The numbers in sample codes denote time in months. The height of each pie chart slice corresponds to the rank estimate of the individual classification method for the taxon in question, while the calculated average rank for all classification methods is indicated by the black circle.

The average rank estimates calculated based on the ranks of 20 most abundant bacterial phyla and Proteobacterial classes in the bacterial community according to the five taxonomic classifications (Figure 3) only agreed on the ranks of the most dominant taxonomic groups (e.g., Alpha- and Gammaproteobacteria and Bacteroidetes). The rank estimates for less abundant phyla were variable depending on the specific taxon and taxonomic classification method (e.g., Acidobacteria, Firmicutes, and Verrucomicrobia).

In most cases, the closest rank estimate to the average of all methods was provided by Kaiju, although some notable exceptions included Cyanobacteria in SW0, and several estimates for Epsilonproteobacteria. Moreover, all of the bacterial phyla or Proteobacterial classes of the top 20 most abundant taxa according to all classification methods were present in the NCBI-nr database used with Kaiju. Kaiju/MAR showed higher rank estimates for Ca. Marinimicrobia, Chloroflexi, Gemmatimonadetes, and Verrucomicrobia compared to the average rank of all methods and the Kaiju estimate, while the rank estimates of Firmicutes and Acidobacteria were lower in both cases. Ca. Rokubacteria, present in the top 20 phyla according to NCBI-nr and SILVA databases, was absent from the MAR*_DB_* database. Kraken2 and Bracken yielded very similar rank estimates to each other (except for Acidobacteria in SW0 and SWO8) while often deviating notably from the average rank estimate of all methods. For instance, the rank estimates of Chloroflexi, Gemmatimonadetes, Nitrospirae, and Verrucomicrobia were always lower than the average rank estimate, while the rank estimates of Firmicutes, Deinococcus-Thermus, Fusobacteria, Spirochaetes, and Tenericutes were higher than the average rank estimate. In addition, Ca. Marinimicrobia, Nitrospinae, and Ca. Rokubacteria were absent from the Standard Kraken2 database. The greatest deviations in bacterial phyla ranks from the average of all methods (e.g., Acidobacteria, Cyanobacteria, and Verrucomicrobia) were often obtained by the Amplicon method. Furthermore, unlike the other reference databases used in this study, in the SILVA database, Beta- and Epsilonproteobacteria were not categorized as distinct Proteobacterial classes, and their ranks could not be taken into account in the analysis.

Despite similar average rank patterns in all samples, the proportions of phyla in the bacterial community determined by different taxonomic classification methods were mostly dissimilar, especially for the five most abundant taxa (Tables S7–S11), with the Amplicon method usually deviating the most. Of the top five taxa, the Amplicon method estimated a substantially lower proportion of Gammaproteobacteria and a higher proportion of Bacteroidetes compared to the other classification methods. For instance, the top-ranked Gammaproteobacteria proportion in SWOB4 was 80–86% according to metagenome-based classification methods and 47% according to the Amplicon method, while the proportion of Bacteroidetes in the same sample was 4–8% according to metagenome-based methods and 34% according to the Amplicon method. The deviations in the estimates of lesser taxa ranks among different methods also provided a good indication of proportion deviation tendencies among the methods for a particular taxon. For instance, the rank and proportion estimates of Firmicutes in SW0 were 18 and 9.4% for Bracken, 18 and 7.2% for Kraken2, 13 and 2% for Kaiju, 8 and 0.7% for Amplicon, and 6 and 0.15% for Kaiju/MAR, respectively (Figure 3; Tables S7–S11). Since Kaiju with the NCBI-nr database generally resulted in the highest number of classified reads and the closest rank estimate to the average of all methods on the phylum level, a detailed description of bacterial and archaeal community structure in microcosms according to this method is provided in Figures S6 and S7, respectively, and in Section S1 in Supplementary File S2.

**Figure 4:**
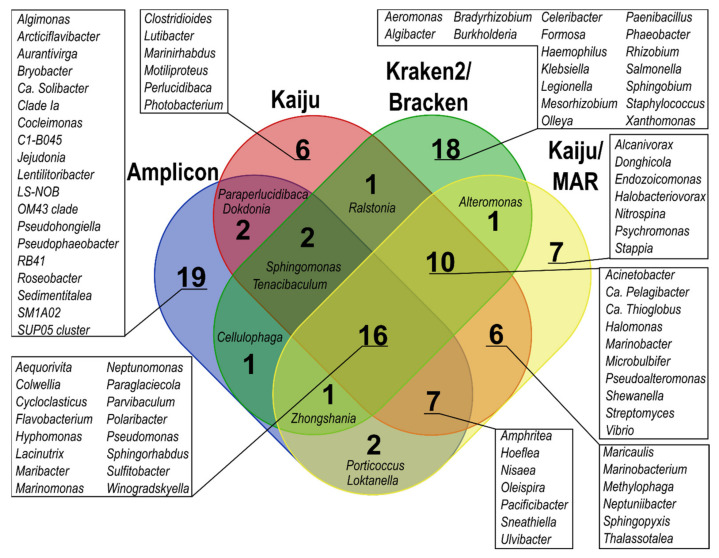
Venn diagram showing the overlap of the 50 predominant bacterial genera between different taxonomic classification methods.

At the genus level, only 16 of the top 50 most predominant bacterial genera across all samples overlapped among all taxonomic classification methods; the overlap increased to 26 genera when only metagenome-based classification methods were considered (Figure 4). Amplicon, Kraken2, and Bracken yielded the highest number of unique genera among the top 50 predominant bacterial genera compared to the other methods. In the detection of genera containing oil hydrocarbon degraders, 108 genera were common to all applied methods, and 191 common genera were found when only metagenome-based classification results were considered (Figure S2). The differences between the estimated proportions within the bacterial community in the same sample according to different classification methods were also substantial (>10%) for several genera, such as *Pseudomonas*, *Cycloclasticus*, *Colwellia*, *Flavobacterium*, and *Polaribacter*. In addition, *Marinomonas*, *Paraperlucidibaca*, *Hyphomonas*, and *Sphingorhabdus* proportions showed over 5% difference for one sample (Tables S12–S16). Notably, Kraken2 and especially Bracken showed considerable proportions (≥5% of the bacterial community) of *Klebsiella* (SW4, SWOB4, SWOB8), *Salmonella* (SW0), and *Staphylococcus* (SW0, SW8) (Tables S14 and S15), which were not among the predominant genera in data obtained with other classification methods.

##### Estimation of Bacterial Community Similarity According to Different Classification Methods

Despite substantial differences in the lists and proportions of the 50 most predominant bacterial genera according to different taxonomic assignment methods, the clustering analysis indicated separate groupings of SW, SWO, and SWOB samples, with the SWOB treatment showing the most distinct separation in the cases of all metagenome-based classification methods (Figure S3A–D). In the case of the Amplicon method, the SWOB treatment and three SW samples formed separate clusters, while SW0 samples and the SW8.1 sample clustered together with the SWO treatment (Figure S3E). Regardless of the taxonomic assignment method applied, the dominant genera specific to each treatment clustered similarly together on heatmaps. The dominant cluster containing *Pseudomonas*, *Cycloclasticus*, *Sphingorhabdus*, *Marinomonas*, *Sneathiella*, *Ulvibacter*, and *Aequorivita* was specific to the SWOB treatment, while a separate branch of *Hyphomonas* and *Parvibaculum* was dominant in the SWO treatment (Figure S3).

**Figure 5:**
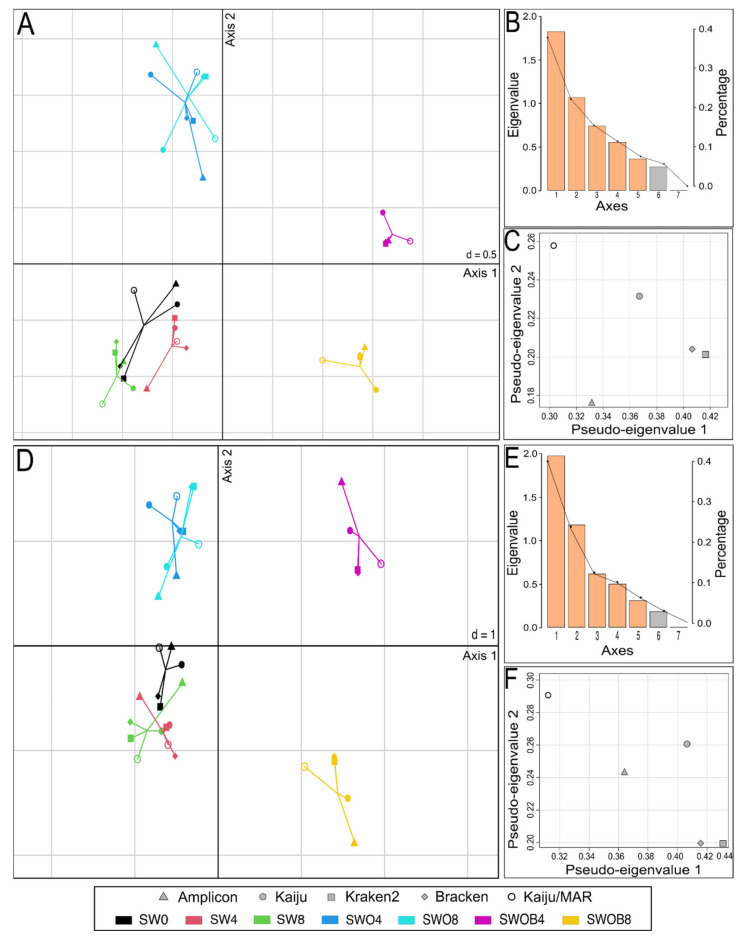
Multiple co-inertia analysis (MCIA) results based on five bacterial genera proportion datasets obtained with different taxonomic assignment methods (Kaiju with NCBI-nr database, Kaiju with MAR*_DB_* database, Kraken2 and Bracken with Standard Kraken2 database, and Amplicon-based sequencing with SILVA database) from seawater (SW), oil-contaminated seawater (SWO), and biostimulated oil-contaminated seawater (SWOB). The numbers in sample codes denote time in months. Subplots (**A**–**C**) depict MCIA results based on proportion data of the 50 predominant bacterial genera, and subplots (**D**–**F**) depict MCIA results based on proportion data of genera containing hydrocarbon-degrading organisms. Subplots (**A**,**D**) present the first two MCIA components in the sample space. Each sample is represented by a shape, where the five datasets for the sample are connected by lines to a center point (global score). (**B**,**E**) are Scree plots of absolute eigenvalues (bars) and the proportions of variance for the eigenvectors (line). (**C**,**F**) are data weighting graphs that show the pseudo-eigenvalue space of all datasets indicating how much variance of an eigenvalue is contributed by each dataset.

The datasets of the proportions of the 50 most prominent bacterial genera in the bacterial community and the proportions of genera containing HDOs according to different taxonomic assignment methods were integrated in the MCIA analysis (Figure 5). In both cases, the most variance was captured by the first MCIA axis (37.6% and 40.8%, respectively), which separated the SWOB treatment from the rest of the samples (Figure 5A,D). The second axis of MCIA captured 22.0% and 24.9% of the variance, respectively, and this axis emphasized the distinction between the samples of SW and SWO treatments.

Projections of all variables in the space of the first two MCIA axes indicated that irrespective of the taxonomic assignment method used, the SWOB treatment was associated with higher proportion values of genera *Pseudomonas*, *Sphingorhabdus*, and *Dietzia*, while three methods out of five also placed *Oleispira* and *Aequorivita* within this group, and two methods out of five included *Paraperlucidibaca* in this group (Figures S4 and S5). Notably, *Oleispira* was missing from the Standard Kraken2 reference database, and *Paraperlucidibaca* was absent from the MAR*_DB_* and Standard Kraken2 reference databases. Regardless of the taxonomic assignment method, the SWO treatment was associated with higher proportions of *Parvibaculum*, *Hyphomonas*, and *Lacinutrix* genera, while amplicon sequencing results also indicated increased proportions of *Jejudonia* and *Cellulophaga* in this treatment. The pseudo-eigenvalue space of genera proportion datasets derived with five different classification methods indicated that Kraken2 and Bracken data contributed the most variance along the first axis, while Kaiju/MAR and Kaiju data contributed high variance to the second axis (Figure 5C,F). The correlation (multivariate generalization of the Pearson correlation coefficient (RV)) between datasets of all four metagenome-based classification methods was RV ≥ 0.9 for the 50 predominant bacterial genera and RV ≥ 0.88 for bacterial genera containing HDOs (Table S17). The correlation of Amplicon data with metagenomic classification methods was markedly lower for predominant genera (RV = 0.81–0.85) and especially in genera containing HDOs (RV = 0.58–0.76).

##### Estimation of Bacterial Genera Proportions via Quantification

In order to estimate genera proportions in the bacterial community via methodology other than sequencing, *Pseudomonas*, *Cycloclasticus*, and *Colwellia* were targeted with genus-specific primers using qPCR, and their quantified abundances were normalized against B16S abundance. These genera could not be quantified from SW0, SW4, and SWO4, as all DNA of these samples had been used to construct sequencing libraries. In the case of *Colwellia*, the proportions determined with sequencing-based and quantification-based methods were reasonably similar; compared to the quantification approach, sequencing-based approaches slightly overestimated *Colwellia* proportions in the SWOB treatment and underestimated them in SW8 and SWO8 (Table 1). In the case of *Cycloclasticus*, all sequencing-based proportion estimates (except for Kraken2 and Bracken of SWOB8) were higher than the quantification-based proportion estimates (Table 1). The difference was especially pronounced for *Cycloclasticus* in SW8, for which proportion estimates obtained by Kaiju (with NCBI-nr database) and Kaiju/MAR exceeded the quantification-derived proportions by 35- and 65-fold, respectively. In the case of the genus *Pseudomonas*, metagenomic-classification-based proportion estimates were generally substantially higher, while the Amplicon proportions were in a similar or even lower range compared to the quantification-based proportions (Table 1). The greatest disagreement was recorded for the SWOB treatment, where Kraken2 and Bracken-based *Pseudomonas* proportions were 48–51% in SWOB4 and 32–34% in SWOB8, while the quantification approach showed that this genus accounted for 9–10% in these two samples.

### 3.3. Hydrocarbon Degradation Potential of Bacterial Community

#### 3.3.1. The Dynamics of Estimated Abundances of Genera Containing Hydrocarbon Degraders

The estimates of proportions of genera containing HDOs ranged from 13.6% to 95.4% in the analyzed samples according to different classification methods (Table S19), with the corresponding estimated abundances ranging from 1.4 × 10^5^ to 1.1 × 10^8^ copies/mL (Figure 2). The characterization of the community was based on classification with Kaiju using the NCBI-nr database, as it resulted in the highest number of detected genera containing HDOs (*n* = 339) in combination with the moderate summed proportion estimate (Figure 2; Table S19).

On day 0, the average proportion and estimated abundance of genera containing HDOs in seawater were 26.6% and 2.7 × 10^5^ copies/mL, respectively (Table S19). The abundances of genera containing HDOs increased throughout the experiment in all treatment variants, and by the end of the experiment, in SW and SWO, the increase was 7- and 9-fold, respectively. However, in the SWOB treatment, the abundances of genera containing HDOs had increased 358-fold by the end of the experiment, reaching a value of 9.7 × 10^7^ copies/mL.

#### 3.3.2. The Dynamics of Hydrocarbon Degradation-Related Genes

**Figure 6:**
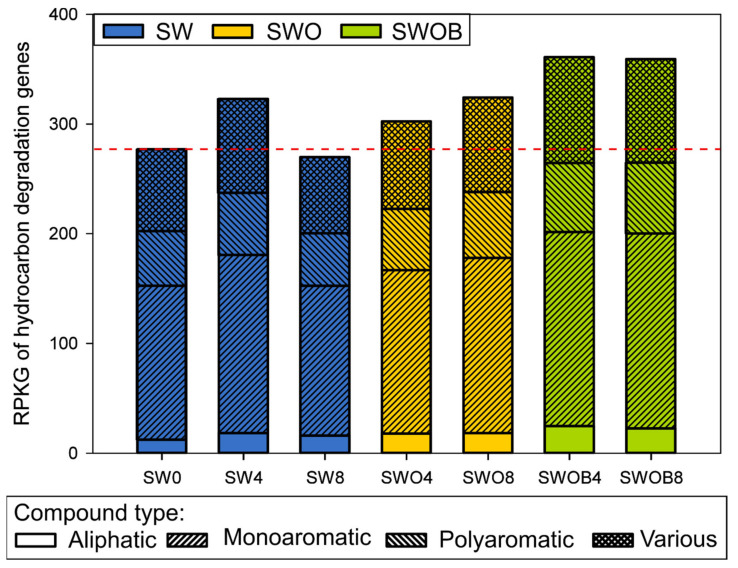
The summarized abundance of detected oil hydrocarbon degradation-related genes, presented as gene-specific reads per kilobase per genome equivalent (RPKG), in microbial communities of seawater (SW), oil-contaminated seawater (SWO), and biostimulated oil-contaminated seawater (SWOB). The numbers in the codes of samples denote incubation time in months. The genes are separated into groups by the compound types whose degradation they are involved in. “Various” means that the gene can be related to degradation pathways of more than one compound type.

A more direct estimation of the microbial community potential for oil hydrocarbon degradation was obtained by analyzing the normalized abundances of 92 HDGs. A total of 82–85 genes were detected across the samples (Table S20). The sum of the normalized abundances of HDGs in initial seawater was 277 RPKG. In SW, this value increased after four months of incubation, but a drop below the initial level was detected by the eighth month (Figure 6). On the other hand, in the SWO treatment, the summed RPKG value and the RPKG values of genes from different functional groups related to the degradation of aliphatic, monoaromatic (MAHs), and polyaromatic (PAHs) compounds only or several compounds simultaneously (“various” group) slightly increased throughout the experiment. The highest increase (1.3-fold) in the RPKG sum compared to SW0 was detected in SWOB4, and this level remained virtually unchanged in SWOB8.

#### 3.3.3. MAGs and Their Oil Hydrocarbon Degradation Potential

**Figure 7:**
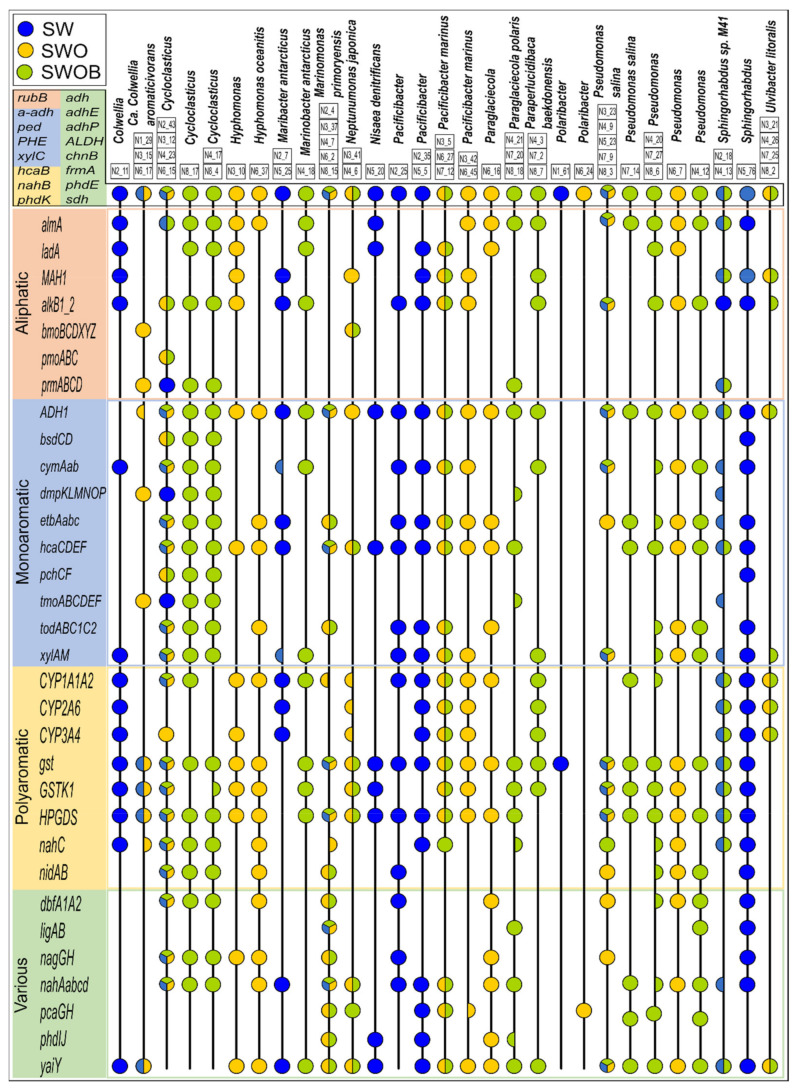
The metagenome-assembled genomes (MAGs) classified to either the genus or species level and belonging to major genera (>2%) based on whole bacterial community analysis and profiles of their oil hydrocarbon degradation genes derived from metagenomes in seawater (SW), oil-contaminated seawater (SWO), and biostimulated oil-contaminated seawater (SWOB). The MAGs with ANI score >95% are presented in one lane, and the characteristics of individual MAGs can be found in Table S21 following their MAG codes (e.g., N2_11). An HDG marker represented by a half-circle indicates that the respective gene was found only in some MAGs from the respective organism lane.

A total of 398 MAGs were created by contig assembly; 195 of these were of good quality (completeness >50% and contamination <10%), which were classified using Kaiju and analyzed for the presence of HDGs (Table S21). Seventy-two of the good-quality MAGs could be classified to at least the genus level. The relatedness analysis of good-quality MAGs indicated that several MAGs can be considered to belong to the same organism (Table S21) and resulted in 130 estimated organisms altogether. The HDG profiles of MAGs representing major genera (>2%) based on the total community analysis of treatments are visualized in Figure 7. The lists of taxonomic affiliations of MAGs and major OTUs determined using the amplicon-based sequencing approach showed considerable overlaps, especially for organisms belonging to major genera in treatments, such as *Colwellia*, *Cycloclasticus*, *Hyphomonas*, *Marinomonas*, *Pacificibacter*, *Paraglaciecola*, *Paraperlucidibaca*, *Pseudomonas*, and *Ulvibacter* (Tables S21 and S22).

The MAGs affiliated to predominant bacterial genera generally had a wide variety of HDGs covering the key steps of pathways for the degradation of aliphatic compounds, MAHs, and PAHs in all treatments, including SW (Figure 7). The MAGs attributed to the most abundant genera in the SWOB treatment, namely, *Cycloclasticus*, *Pseudomonas*, and *Sphingorhabdus*, also had the greatest number of different HDGs detected with relatively equal coverage of degradation pathways of different types of compounds. Another two predominant bacterial genera in the SWOB treatment, *Marinomonas* and *Paraperlucidibaca*, were also well represented, with several MAGs classified to the species level as *M. primoryensis* and *P. baekdonensis*, respectively. Their HDG profiles were more specific: all MAGs classified as *M. primoryensis* lacked genes related to aliphatic compounds degradation, while MAGs classified as *P. baekdonensis* showed a lower representation of genes that can simultaneously belong to degradation pathways of various types of hydrocarbon compounds (Figure 7). MAGs classified as *Cycloclasticus*, *Pseudomonas*, and *M. primoryensis* were detected in all treatments, while other MAGs were more treatment specific.

Fifteen HDGs (mainly different types of alcohol dehydrogenases) were present in all MAGs belonging to major bacterial genera (>2%) in treatments (Figure 7). While other aliphatic compound degradation genes were frequent in MAGs, gene clusters related to the degradation of short gaseous alkanes were rare. For instance, the methane-related *pmoABC* cluster was found only in *Cycloclasticus*, and the butane-related *bmoBCDXYZ* cluster was only detected in *Ca. C. aromaticivorans* and *N. japonica.* Among MAH degradation genes, the anaerobic phenol degradation-related *bsdCD* and 4-cresol dehydrogenase-encoding *pchCF* gene clusters were detected only in MAGs classified as *Cycloclasticus* and *Sphingorhabdus*. In addition, the toluene degradation-related *dmpKLMNOP* and *tmoABCDEF* gene clusters were detected only in MAGs classified as *Ca. C. aromaticivorans*, *Cycloclasticus*, *P. polaris*, and *S.* sp. *M41*. While all the analyzed HDGs related to PAH degradation were identified in numerous MAGs, the protocatechuate-4,5-dioxygenase-encoding *ligAB* gene cluster belonging to degradation pathways of various types of compounds was found only in MAGs classified as *M. primoryensis*, *P. polaris*, *Pseudomonas* (only MAG N4_12), and *Sphingorhabdus*.

In a few cases, the clustering of MAGs based on the ANI score did not agree with the taxonomic classification. For instance, MAG N2_11, classified as *Colwellia*, is located far from MAGs classified as *Ca. C. aromaticivorans*, and their HDG profiles were markedly different (Table S21), while MAG N7_14, classified as *Pseudomonas salina*, was clustered far from other *Pseudomonas salina* MAGs on the taxonomic tree and was not the same organism according to the ANI score. Moreover, N7_14 also missed the *nahC* gene and *nidAB* and *dbfA1A2* gene clusters, characteristic to most of the MAGs classified as *Pseudomonas*. MAG N6_16, attributed to *Paraglaciecola*, was grouped separately and far from MAGs classified as *P. polaris* on the taxonomic tree, and their HDG profiles were quite dissimilar. Finally, while showing similar HDG profiles, MAGs N4_17 and N8_4, classified as *Cycloclasticus*, clustered far from other *Cycloclasticus*-affiliated MAGs on the ANI score-based taxonomic tree.

## 4. Discussion

### 4.1. The Effect of Taxonomic Classification Method on the Estimation of Community Composition in Arctic Seawater-Derived Bacterial Communities

A growing body of Arctic marine microbiology research is characterizing microbial communities using data from either amplicon-based or shotgun metagenomic sequencing [11,13,14,15,42], and the latter is also used for functional profiling. For the taxonomic assignment of shotgun metagenomic data, numerous classifiers and reference databases are available that fall into several categories: (i) DNA-to-DNA methods, where perfect matches between sequence stretches and reference sequences (k-mers) are sought (e.g., Kraken2, Bracken, and PathSeq); (ii) DNA-to-protein methods, where sequence reads are compared with protein-coding sequences (e.g., Kaiju and DIAMOND); and (iii) DNA-to-marker methods, including only specific marker gene families in reference databases (e.g., MetaPhlAn2) [43,44]. However, it has been suggested that the classifier performance and ecological truthfulness and representativeness of the results may vary according to the sample type, taxa present, and composition of the reference database used [43]. Contrary to the results from small, simulated benchmarking datasets [43], in this study, Kaiju classified substantially more reads than Kraken2. This supports the notion that, in the case of environmental metagenomes, Kaiju is able to classify more reads than nucleotide-based methods [21].

The bacterial community structure and taxa proportion estimates varied widely between shotgun metagenomic and amplicon-based data. This discrepancy, characterized by lower proportions of Proteobacteria (especially Gammaproteobacteria) and higher proportions of Bacteroidetes in amplicon-based data compared to metagenome data, seems to be consistent in describing bacterial communities from different water habitats, such as freshwater [45] and Mediterranean seawater [46]. The deviation of amplicon-based taxa proportion estimates compared to metagenome datasets could be attributed to a combination of several factors: the coverage of utilized primers, general PCR bias, variance of copy numbers of the 16S rRNA genes between different taxa, discrepancy of taxon ranks between reference databases, and different sizes and curation levels of reference databases [12,45]. Different metagenome-based classification methods also yielded substantial differences in the estimated proportions of individual taxa at all taxonomic levels, and the overlap of 50 prominent genera between all four metagenome-based classification strategies slightly exceeded 50%. Phylum ranking analysis indicated the possible preferable classification of sequences as Firmicutes, Tenericutes, Fusobacteria, and Spirochaetes by DNA-to-DNA methods and as Ca. Marinimicrobia, Chloroflexi, Gemmatimonadetes, and Verrucomicrobia by DNA-to-protein-based methods. These discrepancies could be caused by different compositions (including the number of references to a specific taxon) and sizes of reference databases used in different methods [36,47].

At the genus level, the estimated proportions of bacterial community dominants in the biostimulation treatment were considerably higher according to metagenome-based methods compared to amplicon-based classifiers: higher proportions were estimated for *Pseudomonas* by all metagenome-based classifiers (especially DNA-to-DNA types) and for *Cycloclasticus* by DNA-to-protein methods. The quantification of their abundances with genera-specific primers verified that the metagenomic classification methods can severely overestimate the proportions of certain organisms in the bacterial community and concordantly affect conclusions based on these results, such as the selection of bioremediation strategy. Such overestimation is probably caused by a combination of several factors. These taxa are represented by disproportionally high numbers of closely related reference sequences in databases (especially *Pseudomonas* in Standard Kraken2 and MAR*_DB_* and *Cycloclasticus* in MAR*_DB_*), leading to oversampling and the decreased accuracy of classifiers [48]. The difference in *Pseudomonas* proportions was probably also affected by the tendency of Kraken2 and Kaiju to overestimate the proportions of microbes with larger genome sizes and higher polyploidy [44]. It is also highly probable that some sequences from the metagenomic data of this experiment were misclassified into these dominant genera; this has been previously noted to be a problem for *Pseudomonas* classifications in Kraken2 [47]. Moreover, the reclassification of species *Pseudomonas salina* and *Pseudomonas sabulinigri*, found to be abundant in this experiment, into a new genus, *Neopseudomonas*, has been recently suggested due to their high variation from the core of the *Pseudomonas* genus [49]. In this study, the metagenome-based classifiers also yielded substantially higher numbers of genera containing hydrocarbon degraders compared to the amplicon-based approach, which may be related to differences in sequencing depth between sequencing methods but also to the known tendency of Kaiju and Kraken2 to predict a large number of low-proportion false-positive taxa [12,43]. The comparison of different sequencing, classification, and quantitative estimations obtained by different analytical tools highlights that the choice of analytical method has a strong effect on practical decisions, such as the applicability of the oil bioremediation method in seawater, made based on microbial community estimations.

Regardless of the large variation in the estimates of several individual taxa proportions, the concordance between the predominant genera proportion datasets among all classifications was high (correlation value RV > 0.8 for all methods and RV ≥ 0.9 for metagenome-based methods). The integration of datasets using a multivariate analysis approach indicated the clear separation of samples according to their bacterial community structure in different treatments, regardless of the taxonomic assignment method used. The MCIA analysis also illustrated that the identification of major genera driving the shift in the bacterial community structure in response to different treatment conditions was similar among all utilized methods. Based on the obtained results, it can be inferred that the integrated analysis of microbial community data produced with different sequencing and taxonomy assignment methods is more informative than the analysis of individual datasets. In addition, the integrated analysis was more efficient in determining the main differences between treatments.

### 4.2. Bacterial Community Potential for Oil Hydrocarbons Degradation in Arctic Seawater

The microbial community in Svalbard seawater used to set up the biostimulation microcosm experiment was rich in Proteobacteria and showed diverse hydrocarbon degradation-related gene profiles and relatively high proportions of the genus *Colwellia*, which suggests the possibility of previous exposure to oil hydrocarbons [50]. However, Arctic seawater is often characterized by low availability of nutrients, such as nitrogen and phosphorus, which limits microbial growth and oil degradation, even if an abundant carbon source is available [3].

Svalbard seawater also seemed to be nutrient deficient, as the oil-contaminated microcosms showed an even lower abundance of prokaryotes than uncontaminated seawater. In addition, no substantial biodegradation of either total oil hydrocarbons, aliphatic hydrocarbons, or PAHs during an eight-month-long incubation was detected in oil-contaminated seawater. However, a clear shift in bacterial community structure in response to oil contamination was identified: by the eighth month, the bloom of *Hyphomonas*, a well-known alkane degradation-related genus [51], that was observed after four months was succeeded by increased proportions of several types of hydrocarbons degrading *Colwellia* [52,53] and especially aromatic hydrocarbons degrading *Marinomonas* [54]. These compound degradation preferences were also well corroborated by the hydrocarbon degradation gene profiles of MAGs classified as the aforementioned genera. In addition, MAGs with diverse HDG profiles attributed to other known oil hydrocarbon-degrading genera such as *Neptunomonas*, *Pseudomonas*, *Paraglaciecola*, *Sulfitobacter*, and *Ulvibacter* and species such as *Maribacter antarcticus* [13,55] were detected from SWO treatment microcosms. It seems that even though the microbial community in Arctic seawater responds to oil contamination and has versatile potential for oil compounds degradation, its abundance in nutrient-deficient conditions is just too low for notable biodegradation of the pollutant.

The oil biodegradation rate in the biostimulation treatment was considerably lower than that reported for dispersant-amended oil-contaminated cold seawater [10,56]. This is probably partly caused by the type of oil spilled. The Troll B type (North Sea naphthenic) crude oil used in this experiment contains a very low concentration of rapidly degradable *n*-alkanes and a high fraction of PAHs, often very recalcitrant in seawater [57,58], as well as an unresolved complex mixture [59], leading to a generally low biodegradation rate [10]. In addition, the oil formed a slick in this experiment, which provides a reduced surface area for microbial colonization and degrades more slowly than dispersed droplets [10,60]. However, the addition of nutrients resulted in substantially elevated prokaryotic community abundance and distinctly different bacterial community structure, as well as biodegradation that contributed to oil components depletion. The bacterial community structure at the phylum level, characterized by markedly increased abundances of Gammaproteobacteria, was similar to the pattern previously reported for dispersed oil degradation in Arctic seawater [9]. Blooms of the Gammaproteobacterial genus *Pseudomonas* and alkane-degrading species *Oleispira antarctica* [13,61] at four months were succeeded after eight months by high proportions of *Cycloclasticus* and *Paraperlucidibaca baekdonensis*, which can degrade both aliphatic and aromatic hydrocarbons via various pathways [11,62]. The hydrocarbon degradation gene profiles of MAGs attributed to these genera also supported this metabolic succession pattern. Notably, these MAGs (among several others) also possessed the *almA* gene related to long-chain alkanes degradation, which is absent from KEGG gene models but seems to play an important role in aliphatic compounds degradation in marine environments. The profiles of the metabolic potential of organisms simultaneously found in high proportions (e.g., *Cycloclasticus* and *P. baekdonensis*) also complemented each other, filling the gaps in degradation pathways found in the genome of each organism. This effect in oil-degrading communities has been noted before [11], albeit not for the exact same taxa. The succession of *Oleispira* and *Cycloclasticus* during oil degradation in Arctic seawater has been reported before, but in a considerably shorter timeframe [10], while *P. baekdonensis* blooms seem to be specific to microbial communities of biostimulated oil-contaminated Arctic environments [62]. The hydrocarbon degradation potential of dominant bacterial genera was supported by smaller proportions of other known hydrocarbon-degrading genera, such as *Neptunomonas*, *Paraglaciecola*, and *Ulvibacter*, and species such as *Marinobacter antarcticus* [13,55], which highlights the various distinctive HDG profiles in the biostimulated microbial community of this study. Similar to previous reports [63], archaea formed a tiny segment of the prokaryotic community in oil-contaminated seawater, and their proportions were especially low in biostimulated oil-contaminated seawater, where essentially only two *Methanosarcina* species survived, suggesting that archaea do not contribute to oil hydrocarbon biodegradation in Arctic seawater.

The results of the current microcosm study show that nutrient addition has a positive effect on the efficiency of crude oil biodegradation in cold conditions, indicating that under field conditions, nutrient delivery via microencapsulation in slow-release particles, combined with dispersants or biosurfactants, may improve biostimulation efficiency [64] and provide a higher oil removal rate in cold seawater.

## 5. Conclusions

The variations detected in the estimates of bacterial community structure and taxa proportions when comparing several different taxonomic classification methods indicate that for Arctic marine microbial communities, direct comparisons between amplicon-based and metagenome-based methods should be avoided or used with extreme care. The metagenome-based classification methods yielded higher numbers of identified genera including oil hydrocarbon degraders, possibly due to the false-positive classification of taxa. However, as the estimated proportions of these genera are very small, they do not notably interfere with the estimation of the dominant segment of the microbial community that potentially contributes the most to oil hydrocarbon degradation in bioremediation approaches. More crucial for the use of microbial community structure estimates for bioremediation planning is the apparent overestimation of proportions of certain dominant taxa involved in oil hydrocarbon degradation (e.g., *Pseudomonas* and *Cycloclasticus*) by metagenome-based classifiers. This suggests that amplicon and shotgun metagenomics-based estimates should be accompanied by other methods with different backgrounds (e.g., qPCR) to achieve ecologically truthful estimates of particular taxa. In addition, the joint analysis based on the integration of several estimates of microbial community structure is less prone to variations in individual taxa proportion estimates provided by different methods, and it can produce a consensus estimate of microbial community dynamics. Based on our results, biostimulation with nutrients promotes naphthenic oil degradation in Arctic seawater, but this strategy alone might not be sufficient to effectively achieve bioremediation goals within a reasonable timeframe. Coupling nutrient addition with the dispersion of oil into smaller droplets could enhance the oil degradation and thus the environmental clean-up rate. However, the effect of adding a combination of nutrients and dispersants on the microbial community structure and oil-degradation potential as well alterations on metabolism of nitrogen, phosphorous and sulphur in Arctic seawater requires further research.

## Figures and Tables

**Table 1:** The proportions (%) of genera *Colwellia*, *Cycloclasticus*, and *Pseudomonas* in the bacterial community of seawater (SW), oil-contaminated seawater (SWO), and biostimulated oil-contaminated seawater (SWOB) based on five taxonomic classification methods and quantification by qPCR (quantified abundances normalized against B16S). The numbers in sample codes denote experimental time in months. NA—not analyzed.

	SW0	SW4	SW8	SWO4	SWO8	SWOB4	SWOB8
*Colwellia*							
Kaiju	3.96	4.94	3.86	4.57	10.87	1.73	0.60
Kaiju/MAR	17.78	8.46	5.18	4.90	13.63	1.86	1.08
Kraken2	8.32	9.97	6.26	4.94	10.83	1.40	0.61
Bracken	6.73	8.16	5.34	4.33	10.05	1.33	0.54
Amplicon	6.45	4.63	3.53	2.75	9.91	1.07	1.80
Quantification	NA	NA	7.35	NA	13.83	0.85	0.34
*Cycloclasticus*							
Kaiju	0.13	10.05	8.81	1.10	2.12	1.82	13.36
Kaiju/MAR	0.25	23.20	16.69	1.75	3.99	2.66	25.33
Kraken2	0.18	1.90	1.48	0.14	0.23	0.21	4.88
Bracken	0.14	1.55	1.25	0.12	0.21	0.20	4.34
Amplicon	0.05	2.13	1.90	0.25	0.46	0.59	7.74
Quantification	NA	NA	0.25	NA	0.01	0.14	7.60
*Pseudomonas*							
Kaiju	1.96	1.23	1.97	9.64	6.42	38.41	25.39
Kaiju/MAR	0.48	0.94	2.24	8.59	9.44	39.03	13.88
Kraken2	3.02	5.68	5.37	13.64	13.06	50.54	34.33
Bracken	2.71	4.74	4.83	12.21	11.97	48.14	31.65
Amplicon	0.26	0.20	0.25	0.78	0.24	10.76	3.70
Quantification	NA	NA	2.59	NA	5.08	9.52	9.25

## Data Availability

The data presented in this study are openly available in https://ebi.ac.uk/ena, PRJEB48192.

## Supplementary Materials

The following are available online at https://www.mdpi.com/article/10.3390/microorganisms9122425/s1, Supplementary File S1: The literature-based list of prokaryotic genera containing oil hydrocarbon-degrading organisms; Supplementary File S2: Table S1: The means and standard deviations of physicochemical characteristics of seawater (SW), oil-contaminated seawater (SWO), and biostimulated oil-contaminated seawater (SWOB); Table S2: The characteristics of qPCR primer pairs and programs; Table S3: The characteristics of metagenomic data of SW, SWO, and SWOB; Table S4: The characteristics of amplicon-based sequencing data of SW, SWO, and SWOB; Table S5: The means and standard deviations of *n*-C17/Pristane and *n*-C18/Phytane ratios and concentrations of PAHs in sterilized oil-contaminated seawater (SWO_S_), SWO, and SWOB; Table S6: The mean abundances and standard deviations of 16S rRNA genes specific to bacteria, archaea, *Colwellia*, *Cycloclasticus*, and *Pseudomonas* in SW, SWO, and SWOB; Table S7: Major bacterial phyla proportions based on taxonomic classification using Kaiju with NCBI-nr database in SW, SWO, and SWOB; Table S8: Major bacterial phyla proportions based on taxonomic classification using Kaiju with MAR*_DB_* database in SW, SWO, and SWOB; Table S9: Major bacterial phyla proportions based on taxonomic classification using Kraken2 with Standard Kraken2 database in SW, SWO, and SWOB; Table S10: Major bacterial phyla proportions based on taxonomic classification using Bracken with Standard Kraken2 database in SW, SWO, and SWOB; Table S11: The mean proportions and standard deviations of major bacterial phyla based on taxonomic classification using amplicon-based sequencing with SILVA database in SW, SWO, and SWOB; Table S12: The proportions of the 50 predominant bacterial genera across all samples in SW, SWO, and SWOB according to Kaiju; Table S13: The proportions of the 50 predominant bacterial genera across all samples in SW, SWO, and SWOB according to Kaiju/MAR; Table S14: The proportions of the 50 predominant bacterial genera across all samples in SW, SWO, and SWOB according to Kraken2; Table S15: The proportions of the 50 predominant bacterial genera across all samples in SW, SWO, and SWOB according to Bracken; Table S16: The mean proportions and standard deviations of the 50 predominant bacterial genera across all samples in SW, SWO, and SWOB according to amplicon-sequencing and SILVA database; Table S17: The correlation between datasets of proportions of the 50 predominant bacterial genera and proportions of bacterial genera containing hydrocarbon degraders acquired with five different taxonomic classification methods; Table S18: The proportions of the most abundant bacterial species in SW, SWO, and SWOB; Table S19: The number of detected genera, proportions, and estimated abundances of bacterial genera containing petroleum hydrocarbon degraders in SW, SWO, and SWOB based on five taxonomic assignment methods; Table S20: Normalized hydrocarbon degradation-related gene abundances (RPKG) in SW, SWO, and SWOB; Table S21: Average nucleotide identity score-based clustering of good-quality (completeness >50%, contamination <10%) metagenome-assembled genomes and their oil degradation gene profiles; Table S22: Major operational taxonomic units of bacterial and archaeal genera found in SW, SWO, and SWOB after four and eight months of incubation according to amplicon-based sequencing; Figure S1: The abundances of bacterial (**A**) and archaeal (**B**) communities, as well as the relative abundance of the archaeal community (**C**) in SW, SWO, and SWOB; Figure S2: Venn diagram showing the overlap of detected genera containing oil hydrocarbon degraders between different taxonomic classification methods; Figure S3: The clustering of SW, SWO, and SWOB microcosms based on the crl-transformed proportions of the 50 most predominant genera of Kaiju (**A**), Kaiju/MAR (**B**), Kraken2 (**C**), Bracken (**D**), and Amplicon (**E**) taxonomic classifications; Figure S4: Multiple co-inertia analysis results based on datasets of the top 50 bacterial genera proportions based on five taxonomic classification methods; Figure S5: Multiple co-inertia analysis results based on datasets of proportions of bacterial genera containing oil hydrocarbon degraders based on five taxonomic classification methods; Figure S6: The bacterial community structure at phylum, genus (>3%), and species (>3%) level in SW, SWO, and SWOB; Figure S7: The proportions of archaeal phyla as well as the most dominant archaeal genera and species in the archaeal community in SW, SWO, and SWOB; Section S1: Prokaryotic community structure in different treatments according to Kaiju.
