# Supplementary material for: Microbial Community Dynamics during Biodegradation of Crude Oil and Its Response to Biostimulation in Svalbard Seawater at Low Temperature
Source: Microorganisms. 2021 Nov 24;9(12):2425. doi: 10.3390/microorganisms9122425 (PMC8707851; doi:10.3390/microorganisms9122425)
Supplement: Supplementary file 1 [file microorganisms-09-02425-s001.zip › Supplementary 1.pdf]

# Microbial Community Dynamics During Biodegradation of Crude Oil and Its Response to Biostimulation in Svalbard Seawater at Low Temperature

Hiie Nõlvak, Nga Phuong Dang, Marika Truu, Angela Peeb, Kertu Tiirik, Megan O'Sadnick, and Jaak Truu

**Supplementary** 1

The list of bacterial genera (A) and archaeal genera (B) containing oil hydrocarbon-degrading organisms (in alphabetical order) based on literature (at the date of 20.10.2021).

| Genus | Class | Phylum | Reference |
|---|---|---|---|
| **A** | | | |
| Abyssivirga/Vallitalea[1] | Clostridia | Firmicutes | [1] |
| Achromobacter | Betaproteobacteria | Proteobacteria | [2] |
| Acidocella | Alphaproteobacteria | Proteobacteria | [3] |
| Acidovorax | Betaproteobacteria | Proteobacteria | [4] |
| Acinetobacter | Gammaproteobacteria | Proteobacteria | [5; 6; 7] |
| Actinomyces | Actinomycetia | Actinobacteria | [8] |
| Actinoplanes | Actinomycetia | Actinobacteria | [8] |
| Actinopolyspora | Actinomycetia | Actinobacteria | [9] |
| Advenella | Betaproteobacteria | Proteobacteria | [10] |
| Aequorivita | Flavobacteriia | Bacteroidetes | [11] |
| Aeribacillus | Bacilli | Firmicutes | [2] |
| Aeromicrobium | Actinomycetia | Actinobacteria | [7] |
| Aeromonas | Gammaproteobacteria | Proteobacteria | [12] |
| Aestuariibacter | Gammaproteobacteria | Proteobacteria | [13] |
| Aestuariicella | Gammaproteobacteria | Proteobacteria | [14] |
| Afipia | Alphaproteobacteria | Proteobacteria | [15] |
| Agrobacterium | Alphaproteobacteria | Proteobacteria | [16] |
| Agrococcus | Actinomycetia | Actinobacteria | [7] |
| Alcaligenes | Betaproteobacteria | Proteobacteria | [17] |
| Alcanivorax | Gammaproteobacteria | Proteobacteria | [2; 13; 18; 19; 20; 21] |
| Algiphilus | Gammaproteobacteria | Proteobacteria | [21] |
| Algoriphagus | Cytophagia | Bacteroidetes | [13] |
| Alicycliphilus | Betaproteobacteria | Proteobacteria | [22] |
| Alicyclobacillus | Bacilli | Firmicutes | [23] |
| Aliiglaciecola | Gammaproteobacteria | Proteobacteria | [24] |
| Alkanibacter | Gammaproteobacteria | Proteobacteria | [25] |
| Alkanindiges | Gammaproteobacteria | Proteobacteria | [7] |
| Altererythrobacter | Alphaproteobacteria | Proteobacteria | [26] |
| Alteromonas | Gammaproteobacteria | Proteobacteria | [2] |
| Amphritea | Gammaproteobacteria | Proteobacteria | [27] |
| Amycolatopsis | Actinomycetia | Actinobacteria | [28] |

| Genus | Class | Phylum | Reference |
|---|---|---|---|
| Anabaena | | Cyanobacteria | [29] |
| Aneurinibacillus | Bacilli | Firmicutes | [30] |
| Anoxybacillus | Bacilli | Firmicutes | [31] |
| Aphanocapsa | | Cyanobacteria | [32] |
| Aphanothece/Coccochloris | | Cyanobacteria | [33] |
| Aquamicrobium | Alphaproteobacteria | Proteobacteria | [34] |
| Arenibacter | Flavobacteriia | Bacteroidetes | [35] |
| Aromatoleum | Betaproteobacteria | Proteobacteria | [36] |
| Arthrobacter | Actinomycetia | Actinobacteria | [7; 37; 38] |
| Azoarcus | Betaproteobacteria | Proteobacteria | [36] |
| Azospirillum | Alphaproteobacteria | Proteobacteria | [7] |
| Azotobacter | Gammaproteobacteria | Proteobacteria | [39] |
| Bacillus | Bacilli | Firmicutes | [2; 40; 41; 42] |
| Beijerinckia | Alphaproteobacteria | Proteobacteria | [43] |
| Bergeyella | Flavobacteriia | Bacteroidetes | [35] |
| Bermanella | Gammaproteobacteria | Proteobacteria | [44; 45] |
| Bizionia | Flavobacteriia | Bacteroidetes | [46] |
| Blastochloris | Alphaproteobacteria | Proteobacteria | [47] |
| Blastococcus | Actinomycetia | Actinobacteria | [7] |
| Bordetella | Betaproteobacteria | Proteobacteria | [48] |
| Bosea | Alphaproteobacteria | Proteobacteria | [38] |
| Brachybacterium | Actinomycetia | Actinobacteria | [49] |
| Brachymonas | Betaproteobacteria | Proteobacteria | [50] |
| Bradyrhizobium | Alphaproteobacteria | Proteobacteria | [38] |
| Brevibacillus | Bacilli | Firmicutes | [51] |
| Brevibacterium | Actinomycetia | Actinobacteria | [52] |
| Brevundimonas | Alphaproteobacteria | Proteobacteria | [53] |
| Burkholderia | Betaproteobacteria | Proteobacteria | [6; 38] |
| Campylobacter | Epsilonproteobacteria | Proteobacteria | [42] |
| Carboxylicivirga | Bacteroidia | Bacteroidetes | [35] |
| Castellaniella | Betaproteobacteria | Proteobacteria | [54] |
| Caulobacter | Alphaproteobacteria | Proteobacteria | [23] |
| Celeribacter | Alphaproteobacteria | Proteobacteria | [13] |
| Cellulomonas | Actinomycetia | Actinobacteria | [7] |
| Cellulosimicrobium | Actinomycetia | Actinobacteria | [16] |
| Chelatococcus | Alphaproteobacteria | Proteobacteria | [55] |
| Chromobacterium | Betaproteobacteria | Proteobacteria | [17] |
| Chromohalobacter | Gammaproteobacteria | Proteobacteria | [56] |
| Chryseobacterium | Flavobacteriia | Bacteroidetes | [35; 57] |
| Citreicella | Alphaproteobacteria | Proteobacteria | [13] |
| Citricoccus | Actinomycetia | Actinobacteria | [7] |
| Citrobacter | Gammaproteobacteria | Proteobacteria | [2] |
| Clavibacter | Actinomycetia | Actinobacteria | [3] |

| Genus | Class | Phylum | Reference |
|---|---|---|---|
| Cloacibacterium | Flavobacteriia | Bacteroidetes | [58] |
| Cobetia | Gammaproteobacteria | Proteobacteria | [59] |
| Colwellia | Gammaproteobacteria | Proteobacteria | [60] |
| Comamonas | Betaproteobacteria | Proteobacteria | [4] |
| Confluentimicrobium | Alphaproteobacteria | Proteobacteria | [61] |
| Corynebacterium | Actinomycetia | Actinobacteria | [6; 52] |
| Croceibacter | Flavobacteriia | Bacteroidetes | [62] |
| Croceicoccus | Alphaproteobacteria | Proteobacteria | [63] |
| Cryobacterium | Actinomycetia | Actinobacteria | [64] |
| Cupriavidus | Betaproteobacteria | Proteobacteria | [65] |
| Cycloclasticus | Gammaproteobacteria | Proteobacteria | [2; 17; 21; 66] |
| Cytophaga | Cytophagia | Bacteroidetes | [67] |
| Dactylococcopsis | | Cyanobacteria | [33] |
| Dechloromonas | Betaproteobacteria | Proteobacteria | [68] |
| Defluviimonas | Alphaproteobacteria | Proteobacteria | [69] |
| Delftia | Betaproteobacteria | Proteobacteria | [70] |
| Desulfatibacillum/Desulfobacillum | Deltaproteobacteria | Proteobacteria | [36] |
| Desulfatiferula | Deltaproteobacteria | Proteobacteria | [71] |
| Desulfatitalea | Deltaproteobacteria | Proteobacteria | [72] |
| Desulfitobacterium | Clostridia | Firmicutes | [73] |
| Desulfobacterium | Deltaproteobacteria | Proteobacteria | [36] |
| Desulfobacula | Deltaproteobacteria | Proteobacteria | [36] |
| Desulfococcus | Deltaproteobacteria | Proteobacteria | [17] |
| Desulfoglaeba | Deltaproteobacteria | Proteobacteria | [36] |
| Desulfosarcina | Deltaproteobacteria | Proteobacteria | [36] |
| Desulfosporosinus | Clostridia | Firmicutes | [36] |
| Desulfothermus | Deltaproteobacteria | Proteobacteria | [74] |
| Desulfotignum | Deltaproteobacteria | Proteobacteria | [36] |
| Desulfotomaculum | Clostridia | Firmicutes | [36] |
| Desulfovibrio | Deltaproteobacteria | Proteobacteria | [75] |
| Devosia | Alphaproteobacteria | Proteobacteria | [38] |
| Diaphorobacter | Betaproteobacteria | Proteobacteria | [76] |
| Dietzia | Actinomycetia | Actinobacteria | [2; 7; 8] |
| Dokdonella | Gammaproteobacteria | Proteobacteria | [77] |
| Dyadobacter | Cytophagia | Bacteroidetes | [42] |
| Dyella | Gammaproteobacteria | Proteobacteria | [78] |
| Echinicola | Cytophagia | Bacteroidetes | [35] |
| Edwardsiella | Gammaproteobacteria | Proteobacteria | [79] |
| Ensifer | Alphaproteobacteria | Proteobacteria | [80] |
| Enterobacter | Gammaproteobacteria | Proteobacteria | [2; 37] |
| Enterococcus | Bacilli | Firmicutes | [41; 81] |
| Eremococcus | Bacilli | Firmicutes | [37] |
| Erwinia | Gammaproteobacteria | Proteobacteria | [82] |

| Genus | Class | Phylum | Reference |
|---|---|---|---|
| Erythrobacter/Lutibacterium | Alphaproteobacteria | Proteobacteria | [83] |
| Escherichia | Gammaproteobacteria | Proteobacteria | [57] |
| Exiguobacterium | Bacilli | Firmicutes | [84] |
| Fabibacter | Cytophagia | Bacteroidetes | [13] |
| Fictibacillus | Bacilli | Firmicutes | [85] |
| Flavobacterium | Flavobacteriia | Bacteroidetes | [41] |
| Franconibacter | Gammaproteobacteria | Proteobacteria | [86] |
| Frankia | Actinomycetia | Actinobacteria | [38] |
| Fusobacterium | Fusobacteriia | Fusobacteria | [87] |
| Gallaecimonas | Gammaproteobacteria | Proteobacteria | [88] |
| Garciella | Clostridia | Firmicutes | [89] |
| Gemmobacter/Catellibacterium | Alphaproteobacteria | Proteobacteria | [26] |
| Geobacillus | Bacilli | Firmicutes | [2] |
| Geobacter | Deltaproteobacteria | Proteobacteria | [68] |
| Georgenia | Actinomycetia | Actinobacteria | [7] |
| Georgfuchsia | Betaproteobacteria | Proteobacteria | [90] |
| Gillisia | Flavobacteriia | Bacteroidetes | [46] |
| Glaciecola | Gammaproteobacteria | Proteobacteria | [91] |
| Gordonia | Actinomycetia | Actinobacteria | [2; 7; 8] |
| Granulosicoccus | Gammaproteobacteria | Proteobacteria | [66] |
| Haemophilus | Gammaproteobacteria | Proteobacteria | [92] |
| Halioxenophilus | Gammaproteobacteria | Proteobacteria | [93] |
| Halomonas | Gammaproteobacteria | Proteobacteria | [59] |
| Halothece | | Cyanobacteria | [33] |
| Herbaspirillum | Betaproteobacteria | Proteobacteria | [94] |
| Herbiconiux | Actinomycetia | Actinobacteria | [95] |
| Herminiimonas | Betaproteobacteria | Proteobacteria | [96] |
| Hoeflea | Alphaproteobacteria | Proteobacteria | [97] |
| Hoyosella/Amycolicicoccus | Actinomycetia | Actinobacteria | [98] |
| Hydrocarboniphaga | Gammaproteobacteria | Proteobacteria | [99] |
| Hydrogenophaga | Betaproteobacteria | Proteobacteria | [100] |
| Hyphomicrobium | Alphaproteobacteria | Proteobacteria | [101] |
| Hyphomonas | Alphaproteobacteria | Proteobacteria | [102] |
| Idiomarina | Gammaproteobacteria | Proteobacteria | [103] |
| Immundisolibacter | Gammaproteobacteria | Proteobacteria | [104] |
| Isoptericola | Actinomycetia | Actinobacteria | [105] |
| Janibacter | Actinomycetia | Actinobacteria | [106] |
| Jannaschia | Alphaproteobacteria | Proteobacteria | [26] |
| Janthinobacterium | Betaproteobacteria | Proteobacteria | [15] |
| Kaistia | Alphaproteobacteria | Proteobacteria | [107] |
| Ketobacter | Gammaproteobacteria | Proteobacteria | [108] |
| Klebsiella | Gammaproteobacteria | Proteobacteria | [12] |
| Kocuria | Actinomycetia | Actinobacteria | [7] |

| Genus | Class | Phylum | Reference |
|---|---|---|---|
| Kordia | Flavobacteriia | Bacteroidetes | [109] |
| Kordiimonas | Alphaproteobacteria | Proteobacteria | [110] |
| Krasilnikoviella | Actinomycetia | Actinobacteria | [7] |
| Kurthia | Bacilli | Firmicutes | [111] |
| Kytococcus | Actinomycetia | Actinobacteria | [112] |
| Labrenzia | Alphaproteobacteria | Proteobacteria | [103] |
| Lactobacillus | Bacilli | Firmicutes | [113] |
| Leclercia | Gammaproteobacteria | Proteobacteria | [114] |
| Legionella | Gammaproteobacteria | Proteobacteria | [115] |
| Leifsonia | Actinomycetia | Actinobacteria | [51] |
| Leptothrix | Betaproteobacteria | Proteobacteria | [15] |
| Leucothrix | Gammaproteobacteria | Proteobacteria | [113] |
| Loktanella | Alphaproteobacteria | Proteobacteria | [26] |
| Luteibacter | Gammaproteobacteria | Proteobacteria | [116] |
| Luteimonas | Gammaproteobacteria | Proteobacteria | [117] |
| Lysinibacillus | Bacilli | Firmicutes | [2] |
| Lysobacter | Gammaproteobacteria | Proteobacteria | [118] |
| Magnetospirillum | Alphaproteobacteria | Proteobacteria | [36] |
| Maribacter | Flavobacteriia | Bacteroidetes | [62] |
| Maribius | Alphaproteobacteria | Proteobacteria | [46] |
| Maricaulis | Alphaproteobacteria | Proteobacteria | [119; 120] |
| Marinobacter | Gammaproteobacteria | Proteobacteria | [13; 21; 66] |
| Marinobacterium | Gammaproteobacteria | Proteobacteria | [121] |
| Marinomonas | Gammaproteobacteria | Proteobacteria | [17] |
| Marinosulfonomonas | Alphaproteobacteria | Proteobacteria | [122] |
| Maritimibacter | Alphaproteobacteria | Proteobacteria | [123] |
| Martelella | Alphaproteobacteria | Proteobacteria | [124] |
| Massilia | Betaproteobacteria | Proteobacteria | [15] |
| Merismopedia/Agmenellum | | Cyanobacteria | [125] |
| Mesoflavibacter | Flavobacteriia | Bacteroidetes | [35] |
| Mesorhizobium | Alphaproteobacteria | Proteobacteria | [38] |
| Methylibium | Betaproteobacteria | Proteobacteria | [126] |
| Methylobacterium | Alphaproteobacteria | Proteobacteria | [38] |
| Methyloversatilis | Betaproteobacteria | Proteobacteria | [127] |
| Microbacterium | Actinomycetia | Actinobacteria | [6; 7] |
| Microbulbifer | Gammaproteobacteria | Proteobacteria | [103] |
| Micrococcus | Actinomycetia | Actinobacteria | [7; 40; 42] |
| Microcoleus | | Cyanobacteria | [8] |
| Micromonospora | Actinomycetia | Actinobacteria | [7; 8] |
| Microvirga | Alphaproteobacteria | Proteobacteria | [128] |
| Moraxella | Gammaproteobacteria | Proteobacteria | [129] |
| Mycobacterium | Actinomycetia | Actinobacteria | [2; 17] |
| Myroides | Flavobacteriia | Bacteroidetes | [35] |

| Genus | Class | Phylum | Reference |
|---|---|---|---|
| N47 | Deltaproteobacteria | Proteobacteria | [130] |
| Neptuniibacter | Gammaproteobacteria | Proteobacteria | [131] |
| Neptunomonas | Gammaproteobacteria | Proteobacteria | [21] |
| Nesiotobacter | Alphaproteobacteria | Proteobacteria | [16] |
| Nevskia | Gammaproteobacteria | Proteobacteria | [132] |
| Nitratireductor | Alphaproteobacteria | Proteobacteria | [16] |
| Nitrobacter | Alphaproteobacteria | Proteobacteria | [38] |
| Nocardia | Actinomycetia | Actinobacteria | [8] |
| Nocardioides | Actinomycetia | Actinobacteria | [7] |
| Nocardiopsis | Actinomycetia | Actinobacteria | [133] |
| Nostoc | | Cyanobacteria | [134] |
| Novosphingobium | Alphaproteobacteria | Proteobacteria | [2; 13; 38] |
| Oceanibaculum | Alphaproteobacteria | Proteobacteria | [135] |
| Oceanicola | Alphaproteobacteria | Proteobacteria | [136; 137] |
| Oceanisphaera | Gammaproteobacteria | Proteobacteria | [138] |
| Oceanobacillus | Bacilli | Firmicutes | [105] |
| Ochrobactrum | Alphaproteobacteria | Proteobacteria | [2] |
| Oleiagrimonas | Gammaproteobacteria | Proteobacteria | [139] |
| Oleibacter | Gammaproteobacteria | Proteobacteria | [140] |
| Oleiphilus | Gammaproteobacteria | Proteobacteria | [21] |
| Oleispira | Gammaproteobacteria | Proteobacteria | [2; 21; 46; 66; 141] |
| Oleomonas | Alphaproteobacteria | Proteobacteria | [142] |
| Oligotropha | Alphaproteobacteria | Proteobacteria | [143] |
| Olivibacter | Sphingobacteriia | Bacteroidetes | [35] |
| Olleya | Flavobacteriia | Bacteroidetes | [35] |
| Oscillatoria | | Cyanobacteria | [29] |
| Paenibacillus | Bacilli | Firmicutes | [4] |
| Pandoraea | Betaproteobacteria | Proteobacteria | [2] |
| Pantoea | Gammaproteobacteria | Proteobacteria | [144] |
| Paraburkholderia | Betaproteobacteria | Proteobacteria | [145] |
| Paracoccus | Alphaproteobacteria | Proteobacteria | [146] |
| Paraglaciecola | Gammaproteobacteria | Proteobacteria | [147] |
| Parapedobacter | Sphingobacteriia | Bacteroidetes | [35] |
| Paraperlucidibaca | Gammaproteobacteria | Proteobacteria | [27] |
| Parvibaculum | Alphaproteobacteria | Proteobacteria | [148] |
| Pasteurella | Gammaproteobacteria | Proteobacteria | [149] |
| Pedobacter | Sphingobacteriia | Bacteroidetes | [35; 150] |
| Pelagibaca | Alphaproteobacteria | Proteobacteria | [13] |
| Pelobacter | Deltaproteobacteria | Proteobacteria | [151] |
| Peptococcus | Clostridia | Firmicutes | [113] |
| Perlucidibaca | Gammaproteobacteria | Proteobacteria | [152] |
| Phenylobacterium | Alphaproteobacteria | Proteobacteria | [38] |
| Phormidium | | Cyanobacteria | [153] |

| Genus | Class | Phylum | Reference |
|---|---|---|---|
| Planococcus | Bacilli | Firmicutes | [154] |
| Planomicrobium | Bacilli | Firmicutes | [155] |
| Plantibacter | Actinomycetia | Actinobacteria | [64] |
| Plectonema | | Cyanobacteria | [32] |
| Polaribacter | Flavobacteriia | Bacteroidetes | [13] |
| Polaromonas | Betaproteobacteria | Proteobacteria | [156] |
| Polycyclovorans | Gammaproteobacteria | Proteobacteria | [21] |
| Polymorphum | Alphaproteobacteria | Proteobacteria | [157] |
| Porphyrobacter | Alphaproteobacteria | Proteobacteria | [158] |
| Porticoccus | Gammaproteobacteria | Proteobacteria | [21] |
| Prauserella | Actinomycetia | Actinobacteria | [159] |
| Profundimonas | Gammaproteobacteria | Proteobacteria | [160] |
| Proteus | Gammaproteobacteria | Proteobacteria | [12] |
| Pseudidiomarina | Gammaproteobacteria | Proteobacteria | [103] |
| Pseudoalteromonas | Gammaproteobacteria | Proteobacteria | [13; 17; 59; 66] |
| Pseudomonas | Gammaproteobacteria | Proteobacteria | [2; 7; 37; 42; 66] |
| Pseudonocardia | Actinomycetia | Actinobacteria | [7; 38] |
| Pseudoxanthomonas | Gammaproteobacteria | Proteobacteria | [161] |
| Psychrobacter | Gammaproteobacteria | Proteobacteria | [66; 162] |
| Pusillimonas | Betaproteobacteria | Proteobacteria | [163] |
| Rahnella | Gammaproteobacteria | Proteobacteria | [164] |
| Ralstonia | Betaproteobacteria | Proteobacteria | [165] |
| Ramlibacter | Betaproteobacteria | Proteobacteria | [166] |
| Rhizobium | Alphaproteobacteria | Proteobacteria | [15] |
| Rhodanobacter | Gammaproteobacteria | Proteobacteria | [167] |
| Rhodobacter | Alphaproteobacteria | Proteobacteria | [168] |
| Rhodococcus | Actinomycetia | Actinobacteria | [2; 8; 13; 66] |
| Rhodoferax | Betaproteobacteria | Proteobacteria | [169] |
| Rhodovulum | Alphaproteobacteria | Proteobacteria | [170] |
| Robiginitomaculum | Alphaproteobacteria | Proteobacteria | [46] |
| Roseivirga | Cytophagia | Bacteroidetes | [13] |
| Roseivivax | Alphaproteobacteria | Proteobacteria | [26] |
| Roseobacter | Alphaproteobacteria | Proteobacteria | [171] |
| Roseomonas | Alphaproteobacteria | Proteobacteria | [7] |
| Roseovarius | Alphaproteobacteria | Proteobacteria | [172] |
| Ruegeria | Alphaproteobacteria | Proteobacteria | [40] |
| Rugosibacter | Betaproteobacteria | Proteobacteria | [173] |
| Saccharothrix | Actinomycetia | Actinobacteria | [174] |
| Salinicola | Gammaproteobacteria | Proteobacteria | [175] |
| Salinisphaera | Gammaproteobacteria | Proteobacteria | [176] |
| Salinivibrio | Gammaproteobacteria | Proteobacteria | [56] |
| Salipiger | Alphaproteobacteria | Proteobacteria | [26] |
| Sanguibacter | Actinomycetia | Actinobacteria | [64] |

| Genus | Class | Phylum | Reference |
|---|---|---|---|
| Sarcina | Clostridia | Firmicutes | [17] |
| Serratia | Gammaproteobacteria | Proteobacteria | [17] |
| Shewanella | Gammaproteobacteria | Proteobacteria | [66] |
| Shinella | Alphaproteobacteria | Proteobacteria | [143] |
| Sinorhizobium | Alphaproteobacteria | Proteobacteria | [15] |
| Smaragdicoccus | Actinomycetia | Actinobacteria | [177] |
| Smithella | Deltaproteobacteria | Proteobacteria | [36] |
| Sneathiella | Alphaproteobacteria | Proteobacteria | [91] |
| Solimonas/Singularimonas | Gammaproteobacteria | Proteobacteria | [176] |
| Sphaerotilus | Betaproteobacteria | Proteobacteria | [178] |
| Sphingobacterium | Sphingobacteriia | Bacteroidetes | [41] |
| Sphingobium | Alphaproteobacteria | Proteobacteria | [2] |
| Sphingomonas | Alphaproteobacteria | Proteobacteria | [2; 37; 38] |
| Sphingopyxis | Alphaproteobacteria | Proteobacteria | [179] |
| Sphingorhabdus | Alphaproteobacteria | Proteobacteria | [180] |
| Spirillum | Betaproteobacteria | Proteobacteria | [67] |
| Spongiibacter | Gammaproteobacteria | Proteobacteria | [46] |
| Staphylococcus | Bacilli | Firmicutes | [2] |
| Stappia | Alphaproteobacteria | Proteobacteria | [105] |
| Stenotrophomonas | Gammaproteobacteria | Proteobacteria | [52] |
| Streptobacillus | Fusobacteriia | Fusobacteria | [2] |
| Streptococcus | Bacilli | Firmicutes | [40] |
| Streptomonospora | Actinomycetia | Actinobacteria | [181] |
| Streptomyces | Actinomycetia | Actinobacteria | [7; 8] |
| Streptosporangium | Actinomycetia | Actinobacteria | [8] |
| Sulfitobacter | Alphaproteobacteria | Proteobacteria | [40] |
| Tenacibaculum | Flavobacteriia | Bacteroidetes | [13] |
| Terrabacter | Actinomycetia | Actinobacteria | [182] |
| Terrimonas | Chitinophagia | Bacteroidetes | [183] |
| Thalassobius | Alphaproteobacteria | Proteobacteria | [184] |
| Thalassolituus | Gammaproteobacteria | Proteobacteria | [21; 185] |
| Thalassospira | Alphaproteobacteria | Proteobacteria | [21; 46] |
| Thauera | Betaproteobacteria | Proteobacteria | [36] |
| Thermoleophilum | Thermoleuphilia | Actinobacteria | [186] |
| Thermus | Deinococci | Deinococcus-Thermus | [187] |
| Thiobacillus | Betaproteobacteria | Proteobacteria | [15] |
| Thioclava | Alphaproteobacteria | Proteobacteria | [13] |
| Tistrella | Alphaproteobacteria | Proteobacteria | [176] |
| Trabulsiella | Gammaproteobacteria | Proteobacteria | [188] |
| Tranquillimonas | Alphaproteobacteria | Proteobacteria | [83] |
| Tropicibacter | Alphaproteobacteria | Proteobacteria | [17] |
| Tropicimonas | Alphaproteobacteria | Proteobacteria | [189] |
| Tsukamurella | Actinomycetia | Actinobacteria | [190] |

| Genus | Class | Phylum | Reference |
|---|---|---|---|
| Ulvibacter | Flavobacteriia | Bacteroidetes | [191] |
| Vallitalea | Clostridia | Firmicutes | [36] |
| Variovorax | Betaproteobacteria | Proteobacteria | [192] |
| Vibrio | Gammaproteobacteria | Proteobacteria | [193] |
| Weeksella | Flavobacteriia | Bacteroidetes | [194] |
| Williamsia | Actinomycetia | Actinobacteria | [7] |
| Winogradskyella | Flavobacteriia | Bacteroidetes | [13] |
| Xanthobacter | Alphaproteobacteria | Proteobacteria | [195] |
| Xanthomonas | Gammaproteobacteria | Proteobacteria | [17] |
| Xylanimicrobium | Actinomycetia | Actinobacteria | [7] |
| Xylella | Gammaproteobacteria | Proteobacteria | [194] |
| Yeosuana | Flavobacteriia | Bacteroidetes | [35] |
| Zavarzinia | Alphaproteobacteria | Proteobacteria | [196] |
| Zhongshania | Gammaproteobacteria | Proteobacteria | [197] |
| Zobellella | Gammaproteobacteria | Proteobacteria | [198] |
| Zobellia | Flavobacteriia | Bacteroidetes | [13] |
| Zoogloea | Betaproteobacteria | Proteobacteria | [199] |
| Zunongwangia | Flavobacteriia | Bacteroidetes | [200] |
| **B** | | | |
| Archaeoglobus | Archaeoglobi | Euryarchaeota | [36] |
| Geoglobus | Archaeoglobi | Euryarchaeota | [36] |
| Haloarcula | Halobacteria | Euryarchaeota | [201] |
| Halobacterium | Halobacteria | Euryarchaeota | [201] |
| Halococcus | Halobacteria | Euryarchaeota | [201] |
| Haloferax | Halobacteria | Euryarchaeota | [201] |
| Halorientalis | Halobacteria | Euryarchaeota | [202] |
| Halorubrum | Halobacteria | Euryarchaeota | [201] |
| Haloterrigena | Halobacteria | Euryarchaeota | [201] |
| Halovivax | Halobacteria | Euryarchaeota | [203] |
| Natrialba | Halobacteria | Euryarchaeota | [201] |
| Natronococcus | Halobacteria | Euryarchaeota | [204] |
| Natronolimnobius | Halobacteria | Euryarchaeota | [204] |
| Natronomonas | Halobacteria | Euryarchaeota | [204] |

[1] According to Postec *et al* (2017) the genus *Abyssivirga* should be reassigned to the genus *Vallitalea* [205].


1. Schouw, A.; Eide, T.L.; Stokke, R.; Pedersen, R.B.; Steen, I.H.; Bødtker, G. Abyssivirga alkaniphila gen. nov.; sp. nov.; an alkane-degrading, anaerobic bacterium from a deep-sea hydrothermal vent system, and emended descriptions of Natranaerovirga pectinivora and Natranaerovirga hydrolytica. *Int. J. Syst. Evol. Microbiol.* **2016**, *66*(4), 1724–1734, doi:10.1099/ijsem.0.000934.
2. Xu, X.; Liu, W.; Tian, S.; Wang, W.; Qi, Q.; Jiang, P.; Gao, X.; Li, F.; Li, H.; Yu, H. Petroleum Hydrocarbon-Degrading Bacteria for the Remediation of Oil Pollution Under Aerobic Conditions: A Perspective Analysis. *Front. Microbiol.* **2018**, *9*, doi:10.3389/fmicb.2018.02885.

3. Dore, S.Y.; Clancy, Q.E.; Rylee, S.M.; Kulpa, C.F. Naphthalene-utilizing and mercury-resistant bacteria isolated from an acidic environment. *Appl. Microbiol. Biotechnol*. **2003**, *63*(2), 194–199, doi:10.1007/s00253-003-1378-4.
4. Meyer, S.; Moser, R.; Neef, A.; Stahl, U.; Kämpfer, P. Differential detection of key enzymes of polyaromatic-hydrocarbon-degrading bacteria using PCR and gene probes. *Microbiology*, **1999**, *145*(7), 1731–1741, doi:10.1099/13500872-145-7-1731.
5. Fatajeva, E.; Gailiūtė, I.; Paliulis, D.; Grigiškis, S. The use of Acinetobacter sp. for oil hydrocarbon degradation in saline waters. *Biologija*, **2014**, *60*(3), doi:10.6001/biologija.v60i3.2971.
6. Muthukamalam, S.; Sivagangavathi, S.; Dhrishya, D.; Sudha Rani, S. Characterization of dioxygenases and biosurfactants produced by crude oil degrading soil bacteria. *Braz. J. Microbiol.* **2017**, 48(4), 637–647, doi:10.1016/j.bjm.2017.02.007.
7. Ali, N.; Dashti, N.; Khanafer, M.; Al-Awadhi, H.; Radwan, S. Bioremediation of soils saturated with spilled crude oil. *Sci. Rep*. **2020**, *10*(1), doi:10.1038/s41598-019-57224-x.
8. Shakya, M.; Verma, P.; Kumar, S.; Sandhu, S.S. Microbes: A Novel Source of Bioremediation for Degradation of Hydrocarbons. In *Microbial Rejuvenation of Polluted Environment*.Springer, Singapore, **2021**, pp. 247–261, doi:10.1007/978-981-15-7447-4_10.
9. Hamedi, J.; Mohammadipanah, F.; Ventosa, A. Systematic and biotechnological aspects of halophilic and halotolerant actinomycetes. *Extremophiles*. **2013**, *17*, 1–13, doi:10.1007/s00792-012-0493-5.
10. Wang, X.; Jin, D.; Zhou, L.; Wu, L.; An, W.; Zhao, L. Draft Genome Sequence of Advenella kashmirensis Strain W13003, a Polycyclic Aromatic Hydrocarbon-Degrading Bacterium. *Genome Announc*. **2014**, *2*(1), doi:10.1128/genomea.00003-14.
11. Perez Calderon, L.J.; Gontikaki, E.; Potts, L.D.; Shaw, S.; Gallego, A.; Anderson, J.A.; Witte, U. Pressure and temperature effects on deep-sea hydrocarbon-degrading microbial communities in subarctic sediments. *MicrobiologyOpen*, **2019**, *8*(6), doi:10.1002/mbo3.768.
12. Odokuma, L.O.; Dickson, A.A. Bioremediation of a crude oil polluted tropical rain forest soil. *Glob. J. Environ. Sci*. **2004**, *2*(1), 29-40, doi:10.4314/gjes.v2i1.2403.
13. Wang, W.; Zhong, R.; Shan, D.; Shao, Z. Indigenous oil-degrading bacteria in crude oil-contaminated seawater of the Yellow sea, China. *Appl. Microbiol. Biotechnol*. **2014**, *98*(16), 7253–7269, doi:10.1007/s00253-014-5817-1.
14. Lo, N.; Kim, K.H.; Baek, K.; Jia, B.; Jeon, C.O. Aestuariicella hydrocarbonica gen. nov.; sp. Nov.; an aliphatic hydrocarbon-degrading bacterium isolated from a sea tidal flat. *Int. J. Syst. Evol. Microbiol.* **2015**, *65*(6), 1935–1940, doi:10.1099/ijs.0.000199.
15. Bodour, A.A.; Wang, J.M.; Brusseau, M.L.; Maier, R.M. Temporal change in culturable phenanthrene degraders in response to long-term exposure to phenanthrene in a soil column system. *Environ. Microbiol*. **2003**, *5*(10), 888–895, doi:10.1046/j.1462-2920.2003.00481.x.
16. Dashti, N.; Ali, N.; Eliyas, M.; Khanafer, M.; Sorkhoh, N.A.; Radwan, S.S. Most hydrocarbonoclastic bacteria in the total environment are diazotrophic, which highlights their value in the bioremediation of hydrocarbon contaminants. *Microbes Environ*. **2015**, *30*(1), 70–75, doi:10.1264/jsme2.ME14090.
17. Xue, J.; Yu, Y.; Bai, Y.; Wang, L.; Wu, Y. Marine Oil-Degrading Microorganisms and Biodegradation Process of Petroleum Hydrocarbon in Marine Environments: A Review. *Curr. Microbiol*. **2015**, *71*(2), 220–228, doi:10.1007/s00284-015-0825-7.
18. Yakimov, M.M.; Golyshin, P.N.; Lang, S.; Moore, E.R.B.; Abraham, W.R.; Lünsdorf, H.; Timmis, K. N. Alcanivorax borkumensis gen. nov.; sp. nov.; a new, hydrocarbon-

degrading and surfactant-producing marine bacterium. *Int. J. Syst. Bacteriol*. **1998**, *48*(2), 339–348, doi:10.1099/00207713-48-2-339.
19. Hara, A.; Syutsubo, K.; Harayama, S. Alcanivorax which prevails in oil-contaminated seawater exhibits broad substrate specificity for alkane degradation. *Environ. Microbiol*. **2003**, *5*(9), 746–753, doi:10.1046/j.1468-2920.2003.00468.x.
20. Hara, A.; Baik, S.H.; Syutsubo, K.; Misawa, N.; Smits, T.H.M.; Van Beilen, J.B.; Harayama, S. Cloning and functional analysis of alkB genes in Alcanivorax borkumensis SK2. *Environ. Microbiol*. **2004**, *6*(3), 191–197, doi:10.1046/j.1462-2920.2003.00550.x.
21. Berry, D.; Gutierrez, T. Evaluating the detection of hydrocarbon-degrading bacteria in 16S rRNA gene sequencing surveys. *Front. Microbiol*. **2017**, *8*, 896, doi:10.3389/fmicb.2017.00896.
22. Weelink, S.A.B.; Tan, N.C.G.; Ten Broeke, H.; Van Den Kieboom, C.; Van Doesburg, W.; Langenhoff, A.A.M.; Gerritse, J.; Junca, H.; Stams, A.J.M. Isolation and characterization of Alicycliphilus denitrificans strain BC, which grows on benzene with chlorate as the electron acceptor. *Appl. Environ. Microbiol*. **2008**, *74*, 6672–6681, doi:10.1128/AEM.00835-08.
23. Yang, S.; Wen, X.; Zhao, L.; Shi, Y.; Jin, H. Crude oil treatment leads to shift of bacterial communities in soils from the deep active layer and upper permafrost along the China-Russia Crude Oil Pipeline route. *PLoS ONE*, **2014**, *9*(5), doi:10.1371/journal.pone.0096552.
24. Jin, H. M.; Jeong, H.I.; Jeon, C.O. Aliiglaciecola aliphaticivorans sp. nov.; an aliphatic hydrocarbon-degrading bacterium, isolated from a sea-tidal flat and emended description of the genus Aliiglaciecola Jean et al. 2013. *Int. J. Syst. Evol. Microbiol*. **2015**, *65*(5), 1550–1555, doi:10.1099/ijs.0.000133.
25. Friedrich, M.M.; Lipski, A. Alkanibacter difficilis gen. nov.; sp. nov. and Singularimonas variicoloris gen. nov.; sp. nov.; hexane-degrading bacteria isolated from a hexane-treated biofilter. *Int. J. Syst. Evol. Microbiol*. **2008**, *58*(10), 2324–2329, doi:10.1099/ijs.0.65517-0.
26. Harwati, T.U.; Kasai, Y.; Kodama, Y.; Susilaningsih, D.; Watanabe, K. Characterization of Diverse Hydrocarbon-Degrading Bacteria Isolated from Indonesian Seawater. *Microbes Environ*. **2007**, *22*(4), 412–415, doi:10.1264/jsme2.22.412.
27. Murphy, S.M.C.; Bautista, M.A.; Cramm, M.A.; Hubert, C.R.J. Biodegradation of diesel and crude oil by Labrador Sea cold adapted microbial communities. *Appl. Environ. Microbiol*. **2021**, *87*(20), doi:10.1128/aem.00800-21.
28. Bourguignon, N.; Isaac, P.; Alvarez, H.; Amoroso, M.J.; Ferrero, M.A. Enhanced polyaromatic hydrocarbon degradation by adapted cultures of actinomycete strains. *J. Basic Microbiol*. **2014**, *54*(12), 1288–1294, doi:10.1002/jobm.201400262.
29. Gamila, H.A.; Ibrahim, M.B.M.; Abd El-Ghafar, H.H. The role of cyanobacterial isolated strains in the biodegradation of crude oil. *Int. J. Environ. Stud*. **2003**, *60*(5), 435–444, doi:10.1080/0020723032000050367.
30. Godheja, J.; Shekhar, S.K.; Satyanarayan, G.N.V.; Modi, D.R. Antibiotic and Heavy Metal Tolerance of Some Indigenous Bacteria Isolated From Petroleum Contaminated Soil Sediments with A Study of Their Aromatic Hydrocarbon Degradation Potential. *Int. J. Curr. Microbiol. Appl. Sci*. **2017**, *6*(3), 194–211, doi:10.20546/ijcmas.2017.603.021.
31. Yusoff, D.F.; Rahman, R.N.Z.R.A.; Masomian, M.; Ali, M.S.M.; Leow, T.C. Newly isolated alkane hydroxylase and lipase producing geobacillus and anoxybacillus species involved in crude oil degradation. *Catalysts*, **2020**, *10*(8), doi:10.3390/catal10080851.
32. Raghukumar, C.; Vipparty, V.; David, J.J.; Chandramohan, D. Degradation of crude oil by marine cyanobacteria. *Appl. Microbiol. Biotechnol*. **2001**, *57*(3), 433–436, doi:10.1007/s002530100784.

33. Abed, R.M.M.; Köster, J. The direct role of aerobic heterotrophic bacteria associated with cyanobacteria in the degradation of oil compounds. *Int. Biodeterior. Biodegradation*, **2005**, *55*(1), 29–37, doi:10.1016/j.ibiod.2004.07.001.
34. Tzintzun-Camacho, O.; Loera, O.; Ramírez-Saad, H.C.; Gutiérrez-Rojas, M. Comparison of mechanisms of hexadecane uptake among pure and mixed cultures derived from a bacterial consortium. *Int. Biodeterior. Biodegradation,* **2012**, *70,* 1–7, doi:10.1016/j.ibiod.2012.01.009.
35. Kwon, K.; Kwon, Y.M.; Kim, S.-J. Aerobic Hydrocarbon-Degrading Bacteroidetes. In *Taxonomy, Genomics and Ecophysiology of Hydrocarbon-Degrading Microbes.* Springer International Publishing, **2019**, pp. 1–19, doi:10.1007/978-3-319-60053-6_7-1.
36. Zhang, C.; Meckenstock, R.U.; Weng, S.; Wei,G.; Hubert, C.R.J.; Wang, J.H.; Dong, X. Marine sediments harbor diverse archaea and bacteria with the potential for anaerobic hydrocarbon degradation via fumarate addition. *FEMS Microbiol. Ecol.* **2021**, *97*(5), doi:10.1093/femsec/fiab045.
37. Bučková, M.; Puškarová, A.; Chovanová, K.; Kraková, L.; Ferianc, P.; Pangallo, D. A simple strategy for investigating the diversity and hydrocarbon degradation abilities of cultivable bacteria from contaminated soil. *World J. Microbiol. Biotechnol.* **2013**, *29*(6), 1085–1098, doi:10.1007/s11274-013-1277-5.
38. Yang, S.; Wen, X.; Shi, Y.; Liebner, S.; Jin, H.; Perfumo, A. Hydrocarbon degraders establish at the costs of microbial richness, abundance and keystone taxa after crude oil contamination in permafrost environments. *Sci. Rep.* **2016**, *6*, 37473, doi:10.1038/srep37473.
39. Gradova, N.B.; Gornova, I.B.; Eddaudi, R.; Salina, R.N. Use of Bacteria of the Genus Azotobacter for Bioremediation of Oil-Contaminated Soils. *Appl. Biochem. Microbiol.* **2003**, *39*(3), 279–281, doi:10.1023/A:1023579628148.
40. Ganesh Kumar, A.; Vijayakumar, L.; Joshi, G.; Magesh Peter, D.; Dharani, G.; Kirubagaran, R. Biodegradation of complex hydrocarbons in spent engine oil by novel bacterial consortium isolated from deep sea sediment. *Bioresour. Technol.* **2014**, *170,* 556–564, doi:10.1016/j.biortech.2014.08.008.
41. Albokari, M.; Mashhour, I.; Alshehri, M.; Boothman, C.; Al-Enezi, M. Characterization of microbial communities in heavy crude oil from Saudi Arabia. *Ann. Microbiol.* **2015**, *65*(1), 95–104, doi:10.1007/s13213-014-0840-0.
42. Olowomofe, T.O.; Oluyege, J.O.; Aderiye, B.I.; Oluwole, O.A. Research article degradation of poly aromatic fractions of crude oil and detection of catabolic genes in hydrocarbon-degrading bacteria isolated from agbabu bitumen sediments in Ondo State. *AIMS Microbiol.* **2019**, *5*(4), 308–323, doi:10.3934/microbiol.2019.4.308.
43. Surovtseva, E.G.; Ivoĭlov, V.S.; Beliaev, S.S. [Physiologo-biochemical properties of a strain of Beijerinckia mobilis 1phi Phn+--a degrader of polycyclic aromatic hydrocarbons]. *Mikrobiologiia*, **1999**, *68*(6), 845–84550.
44. Ribicic, D.; Netzer, R.; Hazen, T.C.; Techtmann, S.M.; Drabløs, F.; Brakstad, O.G. Microbial community and metagenome dynamics during biodegradation of dispersed oil reveals potential key-players in cold Norwegian seawater. *Mar. Pollut. Bull.* **2018**, *129*(1), 370–378, doi:10.1016/j.marpolbul.2018.02.034.
45. Hu, P.; Dubinsky, E.A.; Probst, A.J.; Wang, J.; Sieber, C.M.K.; Tom, L.M.; Gardinali, P.R.; Banfield, J.F.; Atlas, R.M.; Andersen, G.L. Simulation of Deepwater Horizon oil plume reveals substrate specialization within a complex community of hydrocarbon degraders. *Proc. Natl. Acad. Sci. U.S.A.* **2017**, *114*, 7432–7437, doi:10.1073/pnas.1703424114.
46. Guibert, L.M.; Loviso, C.L.; Marcos, M.S.; Commendatore, M.G.; Dionisi, H.M.; Lozada, M. Alkane Biodegradation Genes from Chronically Polluted Subantarctic Coastal

Sediments and Their Shifts in Response to Oil Exposure. *Microb. Ecol.* **2012**, *64*(3), 605–616, doi:10.1007/s00248-012-0051-9.
47. Zengler, K.; Heider, J.; Rosselló-Mora, R.; Widdel, F. Phototrophic utilization of toluene under anoxic conditions by a new strain of Blastochloris sulfoviridis. *Arch. Microbiol.* **1999**, *172*(4), 204–212, doi:10.1007/s002030050761.
48. Pinyakong, O.; Tiangda, K.; Iwata, K.; Omori, T. Isolation of novel phenanthrene-degrading bacteria from seawater and the influence of its physical factors on the degradation of phenanthrene. *ScienceA*sia, **2012**, *38*(1), 36–43, doi:10.2306/scienceasia1513-1874.2012.38.036.
49. Wang, Y.N.; Cai, H.; Chi, C.Q.; Lu, A.H.; Lin, X.G.; Jiang, Z.F.; Wu, X.L. Halomonas shengliensis sp. nov.; a moderately halophilic, denitrifying, crude-oil-utilizing bacterium. *Int. J. Syst. Evol. Microbiol.* **2007**, *57*(6), 1222–1226, doi:10.1099/ijs.0.64973-0.
50. Brzostowicz, P.C.; Walters, D.M.; Jackson, R.E.; Halsey, K.H.; Ni, H.; Rouvière, P.E. Proposed involvement of a soluble methane monooxygenase homologue in the cyclohexane-dependent growth of a new Brachymonas species. *Environ. Microbiol.* **2005**, *7*(2), 179–190, doi:10.1111/j.1462-2920.2004.00681.x.
51. Dashti, N.; Khanafer, M.; El-Nemr, I.; Sorkhoh, N.; Ali, N.; Radwan, S. The potential of oil-utilizing bacterial consortia associated with legume root nodules for cleaning oily soils. *Chemosphere*, **2009**, *74*(10), 1354–1359, doi:10.1016/j.chemosphere.2008.11.028.
52. Linda, A.; Bouziane, A. Petroleum-Oil biodegradation by Corynebacterium aquaticum and Pseudomonas aeruginosa strains isolated from the industrial rejection of the refinery of ARZEW-Algeria. *World Appl. Sci. J.* **2012**, *18*(8), 1119–1123, doi:10.5829/idosi.wasj.2012.18.08.73.
53. Chaîneau, C.H.; Morel, J.; Dupont, J.; Bury, E.; Oudot, J. Comparison of the fuel oil biodegradation potential of hydrocarbon-assimilating microorganisms isolated from a temperate agricultural soil. *Sci. Total Environ.* **1999**, *227*(2–3), 237–247, doi:10.1016/S0048-9697(99)00033-9.
54. Kämpfer, P.; Denger, K.; Cook, A.M.; Lee, S.T.; Jäckel, U.; Denner, E.B.M.; Busse, H.J. Castellaniella gen. nov.; to accommodate the phylogenetic lineage of Alcaligenes defragrans, and proposal of Castellaniella defragrans gen. nov.; comb. nov. and Castellaniella denitrificans sp. nov. *Int. J. Syst. Evol. Microbiol.* **2006**, *56*(4), 815–819, doi:10.1099/ijs.0.63989-0.
55. Salamanca, D.; Engesser, K.H. Isolation and characterization of two novel strains capable of using cyclohexane as carbon source. *Environ. Sci. Pollut. Res.* **2014**, *21*(22), 12757–12766, doi:10.1007/s11356-014-3206-z.
56. Al-Mailem, D.M.; Eliyas, M.; Radwan, S. Enhanced bioremediation of oil-polluted, hypersaline, coastal areas in Kuwait via vitamin-fertilization. *Environ. Sci. Pollut. Res.* **2014**, *21*(5), 3386–3394, doi:10.1007/s11356-013-2293-6.
57. Abbas, S.Z.; Whui, T.C.; Hossain, K.; Ahmad, A.; Rafatullah, M. Isolation and characterization of oil-degrading bacteria from marine sediment environment. *Desalin. Water Treat.* **2018**, *136*, 282–289, doi:10.5004/dwt.2018.23110.
58. Jurelevicius, D.; Alvarez, V.M.; Marques, J.M.; Lima, L.R.F. de S.; Dias, F. de A.; Seldin, L. Bacterial community response to petroleum hydrocarbon amendments in freshwater, marine, and hypersaline water-containing microcosms. *Appl. Environ. Microbiol.* **2013**, *79*(19), 5927–5935, doi:10.1128/AEM.02251-13.
59. Ferguson, R.M.W.; Gontikaki, E.; Anderson, J.A.; Witte, U. The variable influence of dispersant on degradation of oil hydrocarbons in subarctic deep-sea sediments at low temperatures (0-5 °C). *Sci. Rep.* **2017**, *7*(1), doi:10.1038/s41598-017-02475-9.
60. Bælum, J.; Borglin, S.; Chakraborty, R.; Fortney, J.L.; Lamendella, R.; Mason, O.U.; Auer, M.; Zemla, M.; Bill, M.; Conrad, M.E.; et al. Deep-sea bacteria enriched by oil and

dispersant from the Deepwater Horizon spill. *Environ. Microbiol.* **2012**, *14*(9), 2405–2416, doi:10.1111/j.1462-2920.2012.02780.x.
61. Jeong, H.I.; Jin, H.M.; Jeon, C.O. Confluentimicrobium naphthalenivorans sp. Nov.; A naphthalene-degrading bacterium isolated from sea-tidal-flat sediment, and emended description of the genus Confluentimicrobium Park et al. 2015. *Int. J. Syst. Evol. Microbiol.* **2015**, *65*(11), 4191–4195, doi:10.1099/ijsem.0.000561.
62. Catania, V.; Santisi, S.; Signa, G.; Vizzini, S.; Mazzola, A.; Cappello, S.; Yakimov, M.M.; Quatrini, P. Intrinsic bioremediation potential of a chronically polluted marine coastal area. *Mar. Pollut. Bull.* **2015**, *99,* 138–149, doi:10.1016/j.marpolbul.2015.07.042.
63. Huang, Y.; Zeng, Y.; Feng, H.; Wu, Y.; Xu, X. Croceicoccus naphthovorans sp. nov.; a polycyclic aromatic hydrocarbons-degrading and acylhomoserine-lactone-producing bacterium isolated from marine biofilm, and emended description of the genus croceicoccus. *Int. J. Syst. Evol. Microbiol.* **2015**, *65*(5), 1531–1536, doi:10.1099/ijs.0.000132.
64. Lo Giudice, A.; Casella, P.; Caruso, C.; Mangano, S.; Bruni, V.; de Domenico, M.; Michaud, L. Occurrence and characterization of psychrotolerant hydrocarbon-oxidizing bacteria from surface seawater along the Victoria Land coast (Antarctica). *Polar Biol.* **2010**, *33*(7), 929–943, doi:10.1007/s00300-010-0770-7.
65. Wittich, R.M.; Wolff, P. Growth of the genetically engineered strain Cupriavidus necator RW112 with chlorobenzoates and technical chlorobiphenyls. *Microbiology,* **2007**, *153*(1), 186–195, doi:10.1099/mic.0.29096-0.
66. Rizzo, C.; Malavenda, R.; Gerçe, B.; Papale, M.; Syldatk, C.; Hausmann, R.; Bruni, V.; Michaud, L.; Giudice, A.L.; and Amalfitano, S. Effects of a simulated acute oil spillage on bacterial communities from arctic and antarctic marine sediments. *Microorganisms*, **2019**, *7*(12), 632, doi:10.3390/microorganisms7120632.
67. Bossert, I.; Bartha, R. The fate of petroleum in soil ecosystems. In *Petroleum microbiology*. Macmillan, New York, **1984**, pp 435–473.
68. Coates, J.B.; Chakraborty, R.; Lack, J.G.; O'Connor, S.M.; Cole, K.A.; Bender, K.S.; Achenbach, L.A. Anaerobic benzene oxidation coupled to nitrate reduction in pure culture by two strains of Dechloromonas. *Nature*, **2001**, *411*(6841), 1039–1043, doi:10.1038/35082545.
69. Zhang, S.; Sun, C.; Xie, J.; Wei, H.; Hu, Z.; Wang, H. Defluviimonas pyrenivorans sp. nov.; a novel bacterium capable of degrading polycyclic aromatic hydrocarbons. *Int. J. Syst. Evol. Microbiol.* **2018**, *68*(3), 957–961, doi:10.1099/ijsem.0.002629.
70. Shetty, A.R.; de Gannes, V.; Obi, C.C.; Lucas, S.; Lapidus, A.; Cheng, J.F.; Goodwin, L.A.; Pitluck, S.; Peters, L.; Mikhailova, N.; et al. Complete genome sequence of the phenanthrene-degrading soil bacterium Delftia acidovorans Cs1-4. *Stand. Genomic Sci.* **2015**, *10*, 55, doi:10.1186/s40793-015-0041-x.
71. Cravo-Laureau, C.; Labat, C.; Joulian, C.; Matheron, R.; Hirschler-Réa, A. Desulfatiferula olefinivorans gen. nov.; sp. nov.; a long-chain n-alkene-degrading, sulfate-reducing bacterium. *Int. J. Syst. Evol. Microbiol.* **2007**, *57*(11), 2699–2702, doi:10.1099/ijs.0.65240-0.
72. Higashioka, Y.; Kojima, H.; Watanabe, M.; Fukui, M. Desulfatitalea tepidiphila gen. nov.; sp. nov.; a sulfate-reducing bacterium isolated from tidal flat sediment. *Int. J. Syst. Evol. Microbiol.* **2013**, *63*, 761–765, doi:10.1099/ijs.0.043356-0.
73. Kunapuli, U.; Jahn, M.K.; Lueders, T.; Geyer, R.; Heipieper, H.J.; Meckenstock, R.U. Desulfitobacterium aromaticivorans sp. nov. and Geobacter toluenoxydans sp. nov.; iron-reducing bacteria capable of anaerobic degradation of monoaromatic hydrocarbons. *Int. J. Syst. Evol. Microbiol.* **2010**, *60*(3), 686–695, doi:10.1099/ijs.0.003525-0.

74. Kuever, J.; Rainey, F.A.; Widdel, F. Class IV. Deltaproteobacteria class nov. In *Bergey's manual of systematic bacteriology*. Springer, Boston, **2005**, pp 948–955, doi:10.1007/0-387-29298-5_3.
75. Davis, J.B.; Yarbrough, H.F. Anaerobic oxidation of hydrocarbons by Desulfovibrio desulfuricans. *Chem. Geol.* **1966**, *1*, 137–144, doi:10.1016/0009-2541(66)90012-X.
76. Klankeo, P.; Nopcharoenkul, W.; Pinyakong, O. Two novel pyrene-degrading Diaphorobacter sp. and Pseudoxanthomonas sp. isolated from soil. *J. Biosci. Bioeng.* **2009**, *108*(6), 488–495, doi:10.1016/j.jbiosc.2009.05.016.
77. Bacosa, H.P.; Inoue, C. Polycyclic aromatic hydrocarbons (PAHs) biodegradation potential and diversity of microbial consortia enriched from tsunami sediments in Miyagi, Japan. *J. Hazard. Mater*. **2015**, *283*, 689–697, doi:10.1016/j.jhazmat.2014.09.068.
78. Li, A.; Qu, Y.; Zhou, J.; Gou, M. Isolation and characteristics of a novel biphenyl-degrading bacterial strain, Dyella ginsengisoli LA-4. *J. Environ. Sci*. **2009**, *21*(2), 211–217, doi:10.1016/S1001-0742(08)62253-6.
79. Okpokwasili, G.C.; Okorie, B.B. Biodeterioration potentials of microorganisms isolated from car engine lubricating oil. *Tribol. Int.* **1988**, *21*(4), 215–220, doi:10.1016/0301-679X(88)90020-5.
80. Muratova, A.; Pozdnyakova, N.; Makarov, O.; Baboshin, M.; Baskunov, B.; Myasoedova, N.; Golovleva, L.; Turkovskaya, O. Degradation of phenanthrene by the rhizobacterium Ensifer meliloti. *Biodegradation*, **2014**, *25*, 787–795, doi:10.1007/s10532-014-9699-9.
81. Boontawan, A.; Boontawan, P. Isolation and characterization of Jatropha oildegradation by Enterococcus faecalis and burkholderia cenocepacia W-1 under anaerobic condition. *Afr. J. Biotechnol*. **2011**, *10*(63), 13841–13851, doi:10.5897/ajb10.1720.
82. Saadoun, I.; Al-Akhras, M.A.; Abu-Ashour, J. Bacterial degradation of hydrocarbons as evidenced by respirometric analysis. *Microbio*s. **1999**, 1*00*, 19–25.
83. Harwati, T.U.; Kasai, Y.; Kodama, Y.; Susilaningsih, D.; Watanabe, K. Tranquillimonas alkanivorans gen. nov.; sp. nov.; an alkane-degrading bacterium isolated from Semarang Port in Indonesia. *Int. J. Syst. Evol. Microbiol*. **2008**, *58*(9), 2118–2121, doi:10.1099/ijs.0.65817-0.
84. Mohanty, G.; Mukherji, S. Biodegradation rate of diesel range n-alkanes by bacterial cultures Exiguobacterium aurantiacum and Burkholderia cepacia. *Int. Biodeterior. Biodegradation*, **2008**, *61*(3), 240–250, doi:10.1016/j.ibiod.2007.06.011.
85. Pandey, R.; Sharma, P.; Rathee, S.; Singh, H.P.; Batish, D.R.; Krishnamurthy, B.; Kohli, R.K. Isolation and characterization of a novel hydrocarbonoclastic and biosurfactant producing bacterial strain: Fictibacillus phosphorivorans RP3. *3 Biotech*, **2021**, *11*(2), doi:10.1007/s13205-021-02655-5.
86. Pal, S.; Kundu, A.; Banerjee, T.D.; Mohapatra, B.; Roy, A.; Manna, R.; Sar, P.; Kazy, S.K. Genome analysis of crude oil degrading Franconibacter pulveris strain DJ34 revealed its genetic basis for hydrocarbon degradation and survival in oil contaminated environment. *Genomic*s, **2017**, *109*, 374–382, doi:10.1016/j.ygeno.2017.06.002.
87. Brooijmans, R.J.W.; Pastink, M.I.; Siezen, R.J. Hydrocarbon-degrading bacteria: The oil-spill clean-up crew. *Microb. Biotechnol*. **2009**, *2*(6), 587–594, doi:10.1111/j.1751-7915.2009.00151.x.
88. Rodríguez-Blanco, A.; Vetion, G.; Escande, M.L.; Delille, D.; Ghiglione, J.F. Gallaecimonas pentaromativorans gen. nov.; sp. nov.; a bacterium carrying 16S rRNA gene heterogeneity and able to degrade high-molecular-mass polycyclic aromatic hydrocarbons. *Int. J. Syst. Evol. Microbiol*. **2010**, *60*(3), 504–509, doi:10.1099/ijs.0.013532-0.

89. Lavania, M.; Cheema, S.; Sarma, P.M.; Mandal, A.K.; Lal, B. Biodegradation of asphalt by Garciaella petrolearia TERIG02 for viscosity reduc.tion of heavy oil. *Biodegradation*, **2012**, *23*(1), 15–24, doi:10.1007/s10532-011-9482-0.
90. Weelink, S.A.B.; Van Doesburg, W.; Saia, F.T.; Rijpstra, W.I.C.; Röling, W.F.M.; Smidt, H.; Stams, A.J.M. A strictly anaerobic betaproteobacterium Georgfuchsia toluolica gen. nov.; sp. nov. degrades aromatic compounds with Fe(III), Mn(IV) or nitrate as an electron acceptor. *FEMS Microbiol. Ecol.* **2009**, *70*(3), 575–585, doi:10.1111/j.1574-6941.2009.00778.x.
91. Sauret, C.; Séverin, T.; Vétion, G.; Guigue, C.; Goutx, M.; Pujo-Pay, M.; Conan, P.; Fagervold, S.K.; Ghiglione, J.F. "Rare biosphere" bacteria as key phenanthrene degraders in coastal seawaters. *Environ. Pollut.* **2014**, *194*, 246–253, doi:10.1016/j.envpol.2014.07.024.
92. Tyagi, M.; da Fonseca, M.M.R.; de Carvalho, C.C.C.R. Bioaugmentation and biostimulation strategies to improve the effectiveness of bioremediation processes. *Biodegradation*, **2011**, *22*, 231–241, doi:10.1007/s10532-010-9394-4.
93. Iwaki, H.; Yamamoto, T.; Hasegawa, Y. Isolation of marine xylene-utilizing bacteria and characterization of Halioxenophilus aromaticivorans gen. nov.; sp. nov. and its xylene degradation gene cluster. *FEMS Microbiol. Lett.* **2018**, *365*(7), fny042, doi:10.1093/femsle/fny042.
94. Xu, H.X.; Wu, H.Y.; Qiu, Y.P.; Shi, X.Q.; He, G.H.; Zhang, J.F.; Wu, J.C. Degradation of fluoranthene by a newly isolated strain of Herbaspirillum chlorophenolicum from activated sludge. *Biodegradation,* **2011**, *22*(2), 335–345, doi:10.1007/s10532-010-9403-7.
95. Okai, M.; Kihara, I.; Yokoyama, Y.; Ishida, M.; Urano, N. Isolation and characterization of benzo[a]pyrene-degrading bacteria from the Tokyo Bay area and Tama River in Japan. *FEMS Microbiol. Lett.* **2015**, *362*(18), fnv143 doi:10.1093/femsle/fnv143.
96. Kim, S.J.; Park, S.J.; Jung, M.Y.; Kim, J.G.; Madsen, E.L.; Rhee, S.K. An uncultivated nitrate-reducing member of the genus herminiimonas degrades toluene. *Appl. Environ. Microbiol.* **2014**, *80*(10), 3233–3243, doi:10.1128/AEM.03975-13.
97. Rahul, K.; Azmatunnisa, M.; Sasikala, C.H.; Ramana, C.V. Hoeflea olei sp. Nov.; a diesel-oil-degrading, anoxygenic, phototrophic bacterium isolated from backwaters and emended description of the genus Hoeflea. *Int. J. Syst. Evol. Microbiol.* **2015**, *65*(8), 2403–2409, doi:10.1099/ijs.0.000277.
98. Nie, Y.; Fang, H.; Li, Y.; Chi, C.Q.; Tang, Y.Q.; Wu, X.L. The Genome of the Moderate Halophile Amycolicicoccus subflavus DQS3-9A1T Reveals Four Alkane Hydroxylation Systems and Provides Some Clues on the Genetic Basis for Its Adaptation to a Petroleum Environment. *PLoS ONE*, **2013**, *8*(8), e70986, doi:10.1371/journal.pone.0070986.
99. Palleroni, N.J.; Port, A.M.; Chang, H.K.; Zylstra, G.J. Hydrocarboniphaga effusa gen. nov.; sp. nov.; a novel member of the γ-Proteobacteria active in alkane and aromatic hydrocarbon degradation. *Int. J. Syst. Evol. Microbiol.* **2004**, *54*(4), 1203–1207, doi:10.1099/ijs.0.03016-0.
100. Fahy, A.; Ball, A.S.; Lethbridge, G.; Timmis, K.N.; McGenity, T.J. Isolation of alkali-tolerant benzene-degrading bacteria from a contaminated aquifer. Lett. *Appl. Microbiol.* **2008**, *47*(1), 60–66, doi:10.1111/j.1472-765X.2008.02386.x
101. Ozaki, S.; Kishimoto, N.; Fujita, T. Isolation and Phylogenetic Characterization of Microbial Consortia able to Degrade Aromatic Hydrocarbons at High Rates. *Microbes Environ.* **2006**, *21*(1), 44–52, doi:10.1264/jsme2.21.44.
102. Song, W.F.; Wang, J.W.; Yan, Y.C.; An, L.Y.; Zhang, F.; Wang, L.; Xu, Y.; Tian, M.Z.; Nie, Y.; Wu, X.L. Shifts of the indigenous microbial communities from reservoir

production water in crude oil- and asphaltene-degrading microcosms. *Int. Biodeterior. Biodegradation*, **2018**, *132*, 18–29, doi:10.1016/j.ibiod.2018.04.015.
103. Kostka, J.E.; Prakash, O.; Overholt, W.A.; Green, S.J.; Freyer, G.; Canion, A.; Delgardio, J.; Norton, N.; Hazen, T.C.; Huettel, M. Hydrocarbon-degrading bacteria and the bacterial community response in Gulf of Mexico beach sands impacted by the deepwater horizon oil spill. *Appl. Environ. Microbiol.* **2011**, *77*(22), 7962–7974, doi:10.1128/AEM.05402-11.
104. Corteselli, E.M.; Aitken, M.D.; Singleton, D.R. Description of Imm.undisolibacter cernigliae gen. Nov.; sp. nov.; a high-molecular-weight polycyclic aromatic hydrocarbondegrading bacterium within the class Gammaproteobacteria, and proposal of Immundisolibacterales ord. nov. and Immundisolibacteraceae fam. nov. *Int. J. Syst. Evol. Microbiol.* **2017**, *67*, 925–931, doi:10.1099/ijsem.0.001714.
105. Al-Awadhi, H.; Sulaiman, R.H.D.; Mahmoud, H.M.; Radwan, S.S. Alkaliphilic and halophilic hydrocarbon-utilizing bacteria from Kuwaiti coasts of the Arabian Gulf. *Appl. Microbiol. Biotechnol.* **2007**, *77*(1), 183–186, doi:10.1007/s00253-007-1127-1.
106. Yamazoe, A.; Yagi, O.; Oyaizu, H. Degradation of polycyclic aromatic hydrocarbons by a newly isolated dibenzofuran-utilizing Janibacter sp. strain YY-1. *Appl. Microbiol. Biotechnol.* **2004**, *65*(2), 211–218, doi:10.1007/s00253-003-1541-y
107. Al-Mailem, D.M.; Kansour, M.K.; Radwan, S.S. Hydrocarbonoclastic biofilms based on sewage microorganisms and their application in hydrocarbon removal in liquid wastes. *Can. J. Microbiol.* **2014**, *60*(7), 477–486, doi:10.1139/cjm-2014-0214.
108. Kim, S.H.; Kim, J.G.; Jung, M.Y.; Kim, S.J.; Gwak, J.H.; Yu, W.J.; Roh, S.W.; Kim, Y.H.; Rhee, S.K. Ketobacter alkanivorans gen. Nov.; sp. nov.; an n-alkane-degrading bacterium isolated from seawater. *Int. J. Syst. Evol. Microbiol.* **2018**, *68*(7), 2258–2264, doi:10.1099/ijsem.0.002823.
109. Peña-Montenegro, T.D.; Kleindienst, S.; Allen, A.E.; Eren, A.M.; McCrow, J.P.; Sánchez-Calderón, J.D.; Arnold, J.; Joye, S.B. Colwellia and Marinobacter metapangenomes reveal species-specific responses to oil and dispersant exposure in deepsea microbial communities. *BioRxiv*, **2020**, doi:10.1101/2020.09.28.317438.
110. Kwon, K.K.; Lee, H.S.; Yang, S.H.; Kim, S.J. Kordiimonas gwangyangenis gen. nov.; sp. non.; a marine bacterium isolated from marine sediments that forms a distinct lieage (Kordiimonadales ord. nov.) in the "Alphaproteobacteria." *Int. J. Syst. Evol. Microbiol.* **2005**, *55*(5), 2033–2037, doi:10.1099/ijs.0.63684-0.
111. Bisht, S.; Pandey, P.; Sood, A.; Sharma, S.; Bisht, N.S. Biodegradation of naphthalene and anthracene by chemo-tactically active rhizobacteria of Populus deltoides. *Braz. J. Microbiol.* **2010**, *41*(4), 922–930, doi:10.1590/S1517-83822010000400011.
112. Al-Saleh, E.; Drobiova, H.; Obuekwe, C. Predominant culturable crude oil-degrading bacteria in the coast of Kuwait. *Int. Biodeterior. Biodegradation*, **2009**, *63*(4), 400–406, doi:10.1016/j.ibiod.2008.11.004.
113. Floodgate, G.D. The fate of petroleum in marine ecosystems. In *Petroleum microbiology*. Macmillan, New York, **1984**, pp 355–397.
114. Sarma, P.M.; Bhattacharya, D.; Krishnan, S.; Lal, B. Degradation of polycyclic aromatic hydrocarbons by a newly discovered enteric bacterium, Leclercia adecarboxylata. *Appl. Environ. Microbiol.* **2004**, *70*(5), 3163–3166, doi:10.1128/AEM.70.5.3163-3166.2004.
115. Zhang, S.; Wang, Q.; Xie, S. Stable isotope probing identifies anthracene degraders under methanogenic conditions. *Biodegradatio*n, **2012**, *23*(2), 221–230, doi:10.1007/s10532-011-9501-1.
116. Muangchinda, C.; Pansri, R.; Wongwongsee, W.; Pinyakong, O. Assessment of polycyclic aromatic hydrocarbon biodegradation potential in mangrove sediment from Don Hoi Lot,

Samut Songkram Province, Thailand. *J. Appl. Microbiol*. **2013**, *114*(5), 1311–1324, doi:10.1111/jam.12128.
117. Ke, C.Y.; Sun, W.J.; Li, Y.B.; Lu, G.M.; Zhang, Q.Z.; Zhang, X.L. Microbial enhanced oil recovery in Baolige Oilfield using an indigenous facultative anaerobic strain Luteimonas huabeiensis sp. nov. *J. Pet. Sci. Eng*. **2018**, *167*, 160–167, doi:10.1016/j.petrol.2018.04.015.
118. Maeda, R.; Nagashima, H.; Zulkharnain, A.B.; Iwata, K.; Omori, T. Isolation and characterization of a car gene cluster from the naphthalene, phenanthrene, and carbazole-degrading marine isolate Lysobacter sp. strain OC7. *Curr. Microbiol*. **2009**, *59*(2), 154–159, doi:10.1007/s00284-009-9414-y.
119. Yuan, J.; Lai, Q.; Sun, F.; Zheng, T.; Shao, Z. The diversity of PAH-degrading bacteria in a deep-sea water column above the southwest Indian ridge. *Front. Microbiol*. **2015**, *6*, doi:10.3389/fmicb.2015.00853.
120. Seth-Smith, H. “Slick” operation. *Nat. Rev. Microbiol*. **2010**, *8*, 538, doi:10.1038/nrmicro2407.
121. Pham, V.D.; Hnatow, L.L.; Zhang, S.; Fallon, R.D.; Jackson, S.C.; Tomb, J.F.; DeLong, E.F.; Keeler, S.J. Characterizing microbial diversity in production water from an Alaskan mesothermic petroleum reservoir with two independent molecular methods. *Environ. Microbiol*. **2009**, *11*(1), 176–187, doi:10.1111/j.1462-2920.2008.01751.x.
122. Schreiber, L.; Fortin, N.; Tremblay, J.; Wasserscheid, J.; Sanschagrin, S.; Mason, J.; Wright, C.A.; Spear, D.; Johannessen, S.C.; Robinson, B.; et al. In situ microcosms deployed at the coast of British Columbia (Canada) to study dilbit weathering and associated microbial communities under marine conditions. *FEMS Microbiol. Ecol*. **2021**, *97*(7), doi:10.1093/femsec/fiab082.
123. Green, D.H.; Echavarri-Bravo, V.; Brennan, D.; Hart, M.C. Bacterial diversity associated with the coccolithophorid algae emiliania huxleyi and coccolithus pelagicus f. braarudii. *BioMed Res. Int*. **2015**, *2015*, 194540, doi:10.1155/2015/194540.
124. Feng, T.C.; Cui, C.Z.; Dong, F.; Feng, Y.Y.; Liu, Y.D.; Yang, X.M. Phenanthrene biodegradation by halophilic Martelella sp. AD-3. *J. Appl. Microbiol*. **2012**, *113*(4), 779–789, doi:10.1111/j.1365-2672.2012.05386.x.
125. Narro, M.L.; Cerniglia, C.E.; Van Baalen, C.; Gibson, D.T. Metabolism of phenanthrene by the marine cyanobacterium Agmenellum quadruplicatum PR-6. *Appl. Environ. Microbiol*. **1992**, *58*(4), 1351–1359, doi:10.1128/aem.58.4.1351-1359.1992.
126. Nakatsu, C.H.; Hristova, K.; Hanada, S.; Meng, X.Y.; Hanson, J.R.; Scow, K.M.; Kamagata, Y. Methylibium petroleiphilum gen. nov.; sp. nov.; a novel methyl tert-butyl ether-degrading methylotroph of the Betaproteobacteria. *Int. J. Syst. Evol. Microbiol*. **2006**, *56*(5), 983–989, doi:10.1099/ijs.0.63524-0.
127. Kalyuzhnaya, M.G.; De Marco, P.; Bowerman, S.; Pacheco, C.C.; Lara, J.C.; Lidstrom, M.E.; Chistoserdova, L. Methyloversatilis universalis gen. nov.; sp. nov.; a novel taxon within the Betaproteobacteria represented by three methylotrophic isolates. *Int. J. Syst. Evol. Microbiol.* **2006**, *56*(11), 2517–2522, doi:10.1099/ijs.0.64422-0.
128. Simarro, R.; González, N.; Bautista, L.F.; Molina, M.C. Assessment of the efficiency of in situ bioremediation techniques in a creosote polluted soil: Change in bacterial community. *J. Hazard. Mater*. **2013**, *262*, 158–167, doi:10.1016/j.jhazmat.2013.08.025.
129. Stucki, G.; Alexander, M. Role of dissolution rate and solubility in biodegradation of aromatic compounds. *Appl. Environ. Microbiol*. **1987**, *53*(2), 292–297, doi:10.1128/aem.53.2.292-297.1987.
130. Bergmann, F.; Selesi, D.; Weinmaier, T.; Tischler, P.; Rattei, T.; Meckenstock, R.U. Genomic insights into the metabolic potential of the polycyclic aromatic hydrocarbon

degrading sulfate-reducing Deltaproteobacterium N47. *Environ. Microbiol.* **2011**, *13*(5), 1125–1137, doi:10.1111/j.1462-2920.2010.02391.x.

131. Nagashima, H.; Zulkharnain, A.B.; Maeda, R.; Fuse, H.; Iwata, K.; Omori, T. Cloning and Nucleotide Sequences of Carbazole Degradation Genes from Marine Bacterium Neptuniibacter sp. Strain CAR-SF. *Curr. Microbiol.* **2010**, *61*(1), 50–56, doi:10.1007/s00284-009-9575-8.
132. Juteau, P.; Larocque, R.; Rho, D.; LeDuy, A. Analysis of the relative abundance of different types of bacteria capable of toluene degradation in a compost biofilter. *Appl. Microbiol. Biotechnol.* **1999**, *52*(6), 863–868, doi:10.1007/s002530051604.
133. Dixit, V.S.; Pant, A. Hydrocarbon degradation and protease production by Nocardiopsis sp. NCIM 5124. *Lett. Appl. Micr*obiol. **2000**, *30*(1), 67–69, doi:10.1046/j.1472-765x.2000.00665.x.
134. Cerniglia, C.E. Biodegradation of polycyclic aromatic hydrocarbons. *Biodegradation*, **1992**, *3*(2–3), 351–368, doi:10.1007/BF00129093.
135. Lai, Q.; Yuan, J.; Wu, C.; Shao, Z. Oceanibaculum indicum gen. nov.; sp. nov.; isolated from deep seawater of the Indian Ocean. *Int. J. Syst. Evol. Microbiol.* **2009**, *59*(7), 1733–1737, doi:10.1099/ijs.0.004341-0.
136. Hassanshahian, M.; Boroujeni, N.A. Enrichment and identification of naphthalene-degrading bacteria from the Persian Gulf. *Mar. Pollut. Bull.* **2016**, *107*(1), 59–65, doi:10.1016/j.marpolbul.2016.04.020.
137. Gao, X.; Gao, W.; Cui, Z.; Han, B.; Yang, P.; Sun, C.; Zheng, L. Biodiversity and degradation potential of oil-degrading bacteria isolated from deep-sea sediments of South Mid-Atlantic Ridge. *Mar. Pollut. Bull.* **2015**, *97*(1–2), 373–380, doi:10.1016/j.marpolbul.2015.05.065.
138. Buzoleva, L.S.; Bogatyrenko, E.A.; Repina, M.A.; Belkova, N.L. Oil-oxidizing activity of bacteria isolated from south Sakhalin coastal waters. *Microbiology (Russian Federation)*, **2017**, *86*(3), 338–345, doi:10.1134/S0026261717030043.
139. Fang, T.; Wang, H.; Huang, Y.; Zhou, H.; Dong, P. Oleiagrimonas soli gen. nov.; sp. nov.; a genome-sequenced gammaproteobacterium isolated from an oilfield. *Int. J. Syst. Evol. Microbiol.* **2015**, *65*(5), 1666–1671, doi:10.1099/ijs.0.000158.
140. Teramoto, M.; Ohuchi, M.; Hatmanti, A.; Darmayati, Y.; Widyastuti, Y.; Harayama, S.; Fukunaga, Y. Oleibacter marinus gen. nov.; sp. nov.; a bacterium that degrades petroleum aliphatic hydrocarbons in a tropical marine environment. *Int. J. Syst. Evol. Microbiol.* **2011**, *61*(2), 375–380, doi:10.1099/ijs.0.018671-0.
141. Yakimov, M.M.; Giuliano, L.; Gentile, G.; Crisafi, E.; Chernikova, T.N.; Abraham, W.R.; Lünsdorf, H.; Timmis, K.N.; Golyshin, P.N. Oleispira antarctica gen. nov.; sp. nov.; a novel hydrocarbonoclastic marine bacterium isolated from Antarctic coastal sea water. Int. *J. Syst. Evol. Microbiol.* **2003**, *53*(3), 779–785, doi:10.1099/ijs.0.02366-0.
142. Kanamori, T.; Rashid, N.; Morikawa, M.; Atomi, H.; Imanaka, T. Oleomonas sagaranensis gen. nov.; sp. nov.; represents a novel genus in the α-Proteobacteria. *FEMS Microbiol. Lett.* **2002**, *217*(2), 255–261, doi:10.1016/S0378-1097(02)01089-3.
143. Ntougias, S.; Melidis, P.; Navrozidou, E.; Tzegkas, F. Diversity and efficiency of anthracene-degrading bacteria isolated from a denitrifying activated sludge system treating municipal wastewater. *Int. Biodeterior. Biodegradation*, **2015**, *97*, 151–158, doi:10.1016/j.ibiod.2014.11.009.
144. Obuekwe, C.O.; Al-Jadi, Z.K.; Al-Saleh, E.S. Hydrocarbon degradation in relation to cell-surface hydrophobicity among bacterial hydrocarbon degraders from petroleum-contaminated Kuwait desert environment. *Int. Biodeterior. Biodegradation*, **2009**, *63*(3), 273–279, doi:10.1016/j.ibiod.2008.10.004.

145. Lee, Y.; Jeon, C.O. Paraburkholderia aromaticivorans sp. nov.; an aromatic hydrocarbon-degrading bacterium, isolated from gasoline-contaminated soil. *Int. J. Syst. Evol. Microbiol*. **2018**, *68*(4), 1251–1257, doi:10.1099/ijsem.0.002661.
146. Teng, Y.; Luo, Y.; Sun, M.; Liu, Z.; Li, Z.; Christie, P. Effect of bioaugmentation by Paracoccus sp. strain HPD-2 on the soil microbial community and removal of polycyclic aromatic hydrocarbons from an aged contaminated soil. *Bioresour. Technol*. **2010**, *101*(10), 3437–3443, doi:10.1016/j.biortech.2009.12.088.
147. Wang, C.; Huang, Y.; Zhang, Z.; Hao, H.; Wang, H. Absence of the nahG-like gene caused the syntrophic interaction between Marinobacter and other microbes in PAH-degrading process. *J. Hazard. Mater*. **2020**, *384*, doi:10.1016/j.jhazmat.2019.121387.
148. Schleheck, D.; Tindall, B.J.; Rosselló-Mora, R.; Cook, A.M. Parvibaculum lavamentivorans gen. nov.; sp. nov.; a novel heterotroph that initiates catabolism of linear alkylbenzenesulfonate. *Int. J. Syst. Evol. Microbiol*. **2004**, *54*(5), 1489–1497, doi:10.1099/ijs.0.03020-0.
149. Šepie, E.; Bricelj, M.; Leskovšek, H. Biodegradation studies of polyaromatic hydrocarbons in aqueous media. *J. Appl. Microbiol*. **1997**, *83*(5), 561–568, doi:10.1046/j.1365-2672.1997.00261.x.
150. Cai, P.; Cai, P.; Cai, P.; Ning, Z.; Ning, Z.; Liu, Y.; He, Z.; He, Z.; Shi, J.; Niu, M. Diagnosing bioremediation of crude oil-contaminated soil and related geochemical processes at the field scale through microbial community and functional genes. *Ann. Microbiol*. **2020**, *70*(1), doi:10.1186/s13213-020-01580-x.
151. Schink, B. Fermentation of acetylene by an obligate anaerobe, Pelobacter acetylenicus sp. nov. *Arch. Microbiol*. **1985**, *142*(3), 295–301, doi:10.1007/BF00693407.
152. Viggor, S.; Jõesaar, M.; Vedler, E.; Kiiker, R.; Pärnpuu, L.; Heinaru, A. Occurrence of diverse alkane hydroxylase alkB genes in indigenous oil-degrading bacteria of Baltic Sea surface water. *Mar. Pollut. Bull*. **2015**, *101*(2), 507–516, doi:10.1016/j.marpolbul.2015.10.064.
153. Al-Hasan, R.H.; Al-Bader, D.A.; Sorkhoh, N.A.; Radwan, S.S. Evidence for n-alkane consumption and oxidation by filamentous cyanobacteria from oil-contaminated coasts of the Arabian Gulf. *Mar. Biol*. **1998**, *130*(3), 521–527, doi:10.1007/s002270050272.
154. Li, H.; Liu, Y.H.; Luo, N.; Zhang, X.Y.; Luan, T.G.; Hu, J.M.; Wang, Z.Y.; Wu, P.C.; Chen, M.J.; Lu, J.Q. Biodegradation of benzene and its derivatives by a psychrotolerant and moderately haloalkaliphilic Planococcus sp. strain ZD22. *Res. Microbiol*. **2006**, *157*(7), 629–636, doi:10.1016/j.resmic.2006.01.002.
155. Radwan, S.S.; Khanafer, M.M.; Al-Awadhi, H.A. Ability of the so-called obligate hydrocarbonoclastic bacteria to utilize nonhydrocarbon substrates thus enhancing their activities despite their misleading name. *BMC Microbiol*. **2019**, *19*(1), doi:10.1186/s12866-019-1406-x.
156. Jeon, C.O.; Park, W.; Ghiorse, W.C.; Madsen, E.L. Polaromonas naphthalenivorans sp. nov.; a naphthalene-degrading bacterium from naphthalene-contaminated sediment. *Int. J. Syst. Evol. Microbiol*. **2004**, *54*(1), 93–97, doi:10.1099/ijs.0.02636-0.
157. Nie, Y.; Tang, Y.Q.; Li, Y.; Chi, C.Q.; Cai, M.; Wu, X.L. The genome sequence of polymorphum gilvum SL003B-26A1 T reveals its genetic basis for crude oil degradation and adaptation to the saline soil. *PLoS ONE*, **2012**, *7*(2), e31261, doi:10.1371/journal.pone.0031261.
158. Gauthier, E.; Déziel, E.; Villemur, R.; Juteau, P.; Lépine, F.; Beaudet, R. Initial characterization of new bacteria degrading high-molecular weight polycyclic aromatic hydrocarbons isolated from a 2-year enrichment in a two-liquid-phase culture system. *J. Appl. Microbiol*. **2003**, *94*(2), 301–311, doi:10.1046/j.1365-2672.2003.01835.x.

159. Almutairi, A. Prauserella soli sp. nov.; isolated from crude oil-contaminated soil. *Int. J. Syst. Evol. Microbiol.* **2015**, *65*(9), 3060–3065, doi:10.1099/ijs.0.000378.
160. Cao, Y.; Chastain, R.A.; Eloe, E.A.; Nogi, Y.; Kato, C.; Bartletta, D.H. Novel psychropiezophilic oceanospirillales species profundimonas piezophila gen. nov.; sp. nov.; isolated from the deep-sea environment of the puerto rico trench. *Appl. Environ. Microbiol*. **2014**, *80*(1), 54–60, doi:10.1128/AEM.02288-13.
161. Kim, J.M.; Le, N.T.; Chung, B.S.; Park, J.H.; Bae, J.W.; Madsen, E.L.; Jeon, C.O. Influence of soil components on the biodegradation of benzene, toluene, ethylbenzene, and o-, m-, and p-xylenes by the newly isolated bacterium Pseudoxanthomonas spadix BD-a59. *Appl. Environ. Microbiol*. **2008**, *74*(23), 7313–7320, doi:10.1128/AEM.01695-08.
162. Pham, V.H.T.; Jeong, S.W.; Kim, J. Psychrobacillus soli sp. nov.; capable of degrading oil, isolated from oil-contaminated soil. *Int. J. Syst. Evol. Microbiol*. **2015**, *65*(9), 3046–3052, doi:10.1099/ijs.0.000375.
163. Cao, B.; Ma, T.; Ren, Y.; Ren, Y.; Li, G.; Li, P.; Guo, X.; Ding, P.; Feng, L. Complete genome sequence of Pusillimonas sp. T7-7, a cold-tolerant diesel oil-degrading bacterium isolated from the Bohai sea in China. *J. Bacteriol*. **2011**, *193*(15), 4021–4022, doi:10.1128/JB.05242-11.
164. Ma, Y.; Wang, L.; Shao, Z. Pseudomonas, the dominant polycyclic aromatic hydrocarbon-degrading bacteria isolated from Antarctic soils and the role of large plasmids in horizontal gene transfer. *Environ. Microbiol.* **2006**, *8*(3), 455–465, doi:10.1111/j.1462-2920.2005.00911.x.
165. Parales, R.E.; Ditty, J.L.; Harwood, C.S. Toluene-degrading bacteria are chemotactic towards the environmental pollutants benzene, toluene, and trichloroethylene. *Appl. Environ. Microbiol.* **2000**, *66*(9), 4098–4104, doi:10.1128/AEM.66.9.4098-4104.2000.
166. Xie, S.; Sun, W.; Luo, C.; Cupples, A.M. Stable isotope probing identifies novel m-xylene degraders in soil microcosms from contaminated and uncontaminated sites. *Water, Air, Soil Pollut*. **2010**, *212*(1–4), 113–122, doi:10.1007/s11270-010-0326-z.
167. Kanaly, R.A.; Harayama, S.; Watanabe, K. Rhodanobacter sp. strain BPC1 in a benzo[a]pyrene-mineralizing bacterial consortium. *Appl. Environ. Microbiol*. **2002**, *68*(12), 5826–5833, doi:10.1128/AEM.68.12.5826-5833.2002.
168. Crisafi, F.; Giuliano, L.; Yakimov, M.M.; Azzaro, M.; Denaro, R. Isolation and degradation potential of a cold-adapted oil/PAH-degrading marine bacterial consortium from Kongsfjorden (Arctic region). *Rend. Lincei*. **2016**, *27*, 261–270, doi:10.1007/s12210-016-0550-6.
169. Eriksson, S.; Ankner, T.; Abrahamsson, K.; Hallbeck, L. Propylphenols are metabolites in the anaerobic biodegradation of propylbenzene under iron-reducing conditions. *Biodegradation*, **2005**, *16*(3), 253–263, doi:10.1007/s10532-004-1278-z.
170. Ruiz, O.N.; Brown, L.M.; Striebich, R.C.; Smart, C.E.; Bowen, L.L.; Lee, J.S.; Little, B.J.; Mueller, S.S.; Gunasekera, T.S. Effect of Conventional and Alternative Fuels on a Marine Bacterial Community and the Significance to Bioremediation. *Energy Fuels*, **2016**, *30*, 434–444, doi:10.1021/acs.energyfuels.5b02439.
171. Buchan, A.; González, J.M.; Chua, M.J. Aerobic Hydrocarbon-Degrading Alphaproteobacteria: Rhodobacteraceae (Roseobacter). In *Taxonomy, Genomics and Ecophysiology of Hydrocarbon-Degrading Microbes.* Springer International Publishing, **2019**, pp. 1–13, doi:10.1007/978-3-319-60053-6_8-1.
172. Chronopoulou, P. M.; Sanni, G. O.; Silas-Olu, D. I.; van der Meer, J. R.; Timmis, K. N.; Brussaard, C. P. D.; McGenity, T. J. Generalist hydrocarbon-degrading bacterial communities in the oil-polluted water column of the North Sea. *Microb. Biotechnol*. **2015**, *8*(3), 434–447, doi:10.1111/1751-7915.12176.

173. Corteselli, E.M.; Aitken, M.D.; Singleton, D.R. Rugosibacter aromaticivorans gen. Nov.; sp. nov.; a bacterium within the family Rhodocyclaceae, isolated from contaminated soil, capable of degrading aromatic compounds. *Int. J. Syst. Evol. Microbiol.* **2017**, *67*(2), 311–318, doi:10.1099/ijsem.0.001622.
174. Hu, Y.T.; Zou, P.J.; Zhou, Y.G.; Liu, Z.H.; Liu, S.J. Saccharothrix xinjiangensis sp. nov.; a pyrene-degrading actinomycete isolated from Tianchi Lake, Xinjiang, China. *Int. J. Syst. Evol. Microbiol.* **2004**, *54*(6), 2091–2094, doi:10.1099/ijs.0.63143-0.
175. Anan'Ina, L.N.; Plotnikova, E.G.; Gavrish, E.Y.; Demakov, V.A.; Evtushenko, L.I. Salinicola socius gen. nov.; sp. nov.; a moderately halophilic bacterium from a naphthalene-utilizing microbial association. *Microbiology*, **2007**, *76*(3), 324–330, doi:10.1134/S0026261707030095.
176. Wang, L.; Wang, W.; Lai, Q.; Shao, Z. Gene diversity of CYP153A and AlkB alkane hydroxylases in oil-degrading bacteria isolated from the Atlantic Ocean. *Environ. Microbiol.* **2010**, *12*(5), 1230–1242, doi:10.1111/j.1462-2920.2010.02165.x.
177. Adachi, K.; Katsuta, A.; Matsuda, S.; Peng, X.; Misawa, N.; Shizuri, Y.; Kroppenstedt, R.M.; Yokota, A.; Kasai, H. Smaragdicoccus niigatensis gen. nov.; sp. nov.; a novel member of the suborder Corynebacterineae. *Int. J. Syst. Evol. Microbiol.* **2007**, *57*(2), 297–301, doi:10.1099/ijs.0.64254-0.
178. Austin, B.; Calomiris, J.J.; Walker, J.D.; Colwell, R.R. Numerical taxonomy and ecology of petroleum degrading bacteria. *Appl. Environ. Microbiol.* **1977**, *34*(1), 60–68, doi:10.1128/aem.34.1.60-68.1977.
179. Laroe, S.L.; Wang, B.; Han, J.I. Isolation and characterization of a novel polycyclic aromatic hydrocarbon-degrading bacterium, sphingopyxis sp. strain M2R2, capable of passive spreading motility through soil. *Environ. Eng. Sci.* **2010**, *27*(6), 505–512, doi:10.1089/ees.2010.0054.
180. Jeong, H.I.; Jin, H.M.; Jeon, C.O. Complete genome sequence of Sphingorhabdus sp. M41, a versatile hydrocarbon degrader, isolated from crude oil-contaminated costal sediment. *J. Biotechnol.* **2016**, *227*, 41–42, doi:10.1016/j.jbiotec.2016.04.016.
181. Rathore, D.S.; Sheikh, M.; Singh, S.P. Marine Actinobacteria: New Horizons in Bioremediation. In *Recent Developments in Microbial Technologies*. Springer Nature Singapore, **2021**, pp. 425–449, doi:10.1007/978-981-15-4439-2_20.
182. Iida, T.; Mukouzaka, Y.; Nakamura, K.; Kudo, T. Plasmid-borne genes code for an angular dioxygenase involved in dibenzofuran degradation by Terrabacter sp. Strain YK3. *Appl. Environ. Microbiol.* **2002**, *68*(8), 3716–3723, doi:10.1128/AEM.68.8.3716-3723.2002.
183. Song, M.; Luo, C.; Jiang, L.; Zhang, D.; Wang, Y.; Zhang, G. Identification of Benzo[a]pyrene-metabolizing bacteria in forest soils by using DNA-based stable-isotope probing. *Appl. Environ. Microbiol.* **2015**, *81*(21), 7368–7376, doi:10.1128/AEM.01983-15.
184. Rodrigo-Torres, L.; Pujalte, M.J.; Arahal, D.R. Draft genome sequence of Thalassobius gelatinovorus CECT 4357T, a roseobacter with the potential ability to degrade polycyclic aromatic hydrocarbons. *Gene Rep.* **2017**, *9*, 32–36, doi:10.1016/j.genrep.2017.08.005.
185. Yakimov, M.M.; Giuliano, L.; Denaro, R.; Crisafi, E.; Chernikova, T.N.; Abraham, W.R.; Luensdorf, H.; Timmis, K.N.; Golyshin, P.N. Thalassolituus oleivorans gen. nov.; sp. nov.; a novel marine bacterium that obligately utilizes hydrocarbons. *Int. J. Syst. Evol. Microbiol.* **2004**, *54*, 141–148, doi:10.1099/ijs.0.02424-0.
186. Zarilla, K.A.; Perry, J.J. Thermoleophilum album gen. nov. and sp. nov.; a bacterium obligate for thermophily and n-alkane substrates. *Arch. Microbiol.* **1984**, *137*(4), 286–290, doi:10.1007/BF00410723.

187. Feitkenhauer, H.; Müller, R.; Märkl, H. Degradation of polycyclic aromatic hydrocarbons and long chain alkanes at 60-70 °C by Thermus and Bacillus spp. *Biodegradation*, **2003**, *14*(6), 367–372, doi:10.1023/A:1027357615649.
188. Kuppusamy, S.; Thavamani, P.; Megharaj, M.; Lee, Y.B.; Naidu, R. Kinetics of PAH degradation by a new acid-metal-tolerant Trabulsiella isolated from the MGP site soil and identification of its potential to fix nitrogen and solubilize phosphorous. *J. Hazard. Mater*. **2016**, *307*, 99–107, doi:10.1016/j.jhazmat.2015.12.068.
189. Harwati, T.U.; Kasai, Y.; Kodama, Y.; Susilaningsih, D.; Watanabe, K. Tropicimonas isoalkanivorans gen. nov.; sp. nov.; a branched-alkane-degrading bacterium isolated from Semarang Port in Indonesia. *Int. J. Syst. Evol. Microbiol*. **2009**, *59*(2), 388–391, doi:10.1099/ijs.0.65822-0.
190. Hassanshahian, M.; Ahmadinejad, M.; Tebyanian, H.; Kariminik, A. Isolation and characterization of alkane degrading bacteria from petroleum reservoir waste water in Iran (Kerman and Tehran provenances). *Mar. Pollut. Bull*. **2013**, *7*3(1), 300–305, doi:10.1016/j.marpolbul.2013.05.002.
191. Campeão, M.E.; Swings, J.; Silva, B.S.; Otsuki, K.; Thompson, F.L.; Thompson, C.C. "Candidatus Colwellia aromaticivorans" sp. nov.; "Candidatus Halocyntiibacter alkanivorans" sp. nov.; and "Candidatus Ulvibacter alkanivorans" sp. nov. Genome Sequences. *Microbiol. Resour. Announc*. **2019**, *8*(15), doi:10.1128/mra.00086-19.
192. Posman, K.M.; DeRito, C.M.; Madsen, E.L. Benzene degradation by a Variovorax species within a coal tar-contaminated groundwater microbial community. *Appl. Environ. Microbiol*. **2017**, *83*(4), e02658–e02616, doi:10.1128/AEM.02658-16.
193. Hedlund, B.P.; Staley, J.T. Vibrio cyclotrophicus sp. nov.; a polycyclic aromatic hydrocarbon (PAH)-degrading marine bacterium. *Int. J. Syst. Evol. Microbiol*. **2001**, *51*(1), 61–66, doi:10.1099/00207713-51-1-61.
194. Yuste, L.; Corbella, M.E.; Turiégano, M.J.; Karlson, U.; Puyet, A.; Rojo, F. Characterization of bacterial strains able to grow on high molecular mass residues from crude oil processing. *FEMS Microbiol. Ecol*. **2000**, *32*(1), 69–75, doi:10.1016/S0168-6496(00)00015-5.
195. Padden, A.N.; Rainey, F.A.; Kelly, D.P.; Wood, A.P. Xanthobacter tagetidis sp. nov.; an organism associated with Tagetes species and able to grow on substituted thiophenes. *Int. J. Syst. Bacteriol*. **1997**, *47*(2), 394–401, doi:10.1099/00207713-47-2-394.
196. Rochman, F.F.; Sheremet, A.; Tamas, I.; Saidi-Mehrabad, A.; Kim, J.J.; Dong, X.; Sensen, C.W.; Gieg, L.M.; Dunfield, P.F. Benzene and naphthalene degrading bacterial communities in an oil sands tailings pond. *Front. Microbiol*. **2017**, *8*, 1845, doi:10.3389/fmicb.2017.01845.
197. Naysim, L.O.; Kang, H.J.; Jeon, C.O. Zhongshania aliphaticivorans sp. nov.; an aliphatic hydrocarbon-degrading bacterium isolated from marine sediment, And transfer of Spongiibacter borealis Jang et al. 2011 to the genus Zhongshania as Zhongshania borealis comb. nov. *Int. J. Syst. Evol. Microbiol*. **2014**, *64*, 3768–3774, doi:10.1099/ijs.0.068593-0.
198. Lee, D.W.; Lee, H.; Kwon, B.O.; Khim, J.S.; Yim, U.H.; Park, H.; Park, B.; Choi, I.G.; Kim, B.S.; Kim, J.J. Zobellella maritima sp. Nov.; a polycyclic aromatic hydrocarbon-degrading bacterium, isolated from beach sediment. *Int. J. Syst. Evol. Microbiol*. **2018**, *68*(7), 2279–2284, doi:10.1099/ijsem.0.002825.
199. Farkas, M.; Táncsics, A.; Kriszt, B.; Benedek, T.; Tóth, E.M.; Kéki, Z.; Veres, P.G.; Szoboszlay, S. Zoogloea oleivorans sp. nov.; a floc-forming, petroleum hydrocarbon-degrading bacterium isolated from biofilm. *Int. J. Syst. Evol. Microbiol.* **2015**, *65*(1), 274–279, doi:10.1099/ijs.0.068486-0.

200. Shao, R.; Lai, Q.; Liu, X.; Sun, F.; Du, Y.; Li, G.; Shao, Z. Zunongwangia atlantica sp. nov.; isolated from deep-sea water. *Int. J. Syst. Evol. Microbiol*. **2014**, *64*, 16–20, doi:10.1099/ijs.0.054007-0.
201. Krzmarzick, M.J.; Taylor, D.K.; Fu,X.; McCutchan, A.L. Diversity and niche of archaea in bioremediation. *Archaea*, **2018**, *3*, 3194108, doi:10.1155/2018/3194108.
202. Zhao, D.; Kumar, S.; Zhou, J.; Wang, R.; Li, M.; Xiang, H. Isolation and complete genome sequence of Halorientalis hydrocarbonoclasticus sp. nov.; a hydrocarbon-degrading haloarchaeon. *Extremophiles*, **2017**, *21*(6), 1081–1090, doi:10.1007/s00792-017-0968-5.
203. Kebbouche-Gana, S.; Gana, M.L.; Khemili, S.; Fazouane-Naimi, F.; Bouanane, N.A.; Penninckx, M.; Hacene, H. Isolation and characterization of halophilic Archaea able to produce biosurfactants. *J. Ind. Microbiol. Biotechnol*. **2009**, *36*(5), 727–738, doi:10.1007/s10295-009-0545-8.
204. Selim, S.A.; El-Alfy, S.M.; Hagagy, N.I.; Hassanin, A.A.I.; Khattab, R.M.; El-Meleigy, E.S.A.; Abdel Aziz, M.H.; Maugeri, T.L. Oil-biodegradation and biosurfactant production by haloalkaliphilic Archaea isolated from Soda lakes of the Wadi An Natrun, Egypt. *J. Pure Appl. Microbiol*. **2012**, *6*(3), 1011–1020.
205. Postec, A.; Ollivier, B.; Fardeau, M.L. Objection to the proposition of the new genus Abyssivirga. *Int. J. of Syst. Evol. Microbiol*. **2017**, *67*, 174–174, doi:10.1099/ijsem.0.001601.
