# Supplementary material for: Microbial Community Dynamics during Biodegradation of Crude Oil and Its Response to Biostimulation in Svalbard Seawater at Low Temperature
Source: Microorganisms. 2021 Nov 24;9(12):2425. doi: 10.3390/microorganisms9122425 (PMC8707851; doi:10.3390/microorganisms9122425)
Supplement: Supplementary file 1 [file microorganisms-09-02425-s001.zip › Supplementary2.pdf]

Microbial community dynamics during biodegradation of crude oil and its response to biostimulation in Svalbard seawater at low temperature

Hiie Nõlvak, Nga Phuong Dang, Marika Truu, Angela Peeb, Kertu Tiirik, Megan O'Sadnick and Jaak Truu

Supplementary File S2

Supplementary tables

Table S1. The means and standard deviations of physicochemical characteristics of seawater (SW), oil-contaminated seawater (SWO), and biostimulated oil-contaminated seawater (SWOB).

Table S2. The characteristics of qPCR primer pairs and programs.

Table S3. The characteristics of metagenomic data of seawater (SW), oil-contaminated seawater (SWO), and biostimulated oil-contaminated seawater (SWOB).

Table S4. The characteristics of amplicon-based sequencing data of seawater (SW), oil-contaminated seawater (SWO), and biostimulated oil-contaminated seawater (SWOB).

Table S5. The means and standard deviations of *n*-C17/Pristane and *n*-C18/Phytane ratios and concentrations of PAHs in sterilized oil contaminated seawater (SWO_S), oil contaminated seawater (SWO) and biostimulated oil contaminated seawater (SWOB).

Table S6. The mean abundances and standard deviations of 16S rRNA genes specific to bacteria, archaea, *Colwellia*, *Cycloclasticus*, and *Pseudomonas* in the seawater (SW), oil contaminated seawater (SWO) and biostimulated oil contaminated seawater (SWOB).

Table S7. Major bacterial phyla proportions based on taxonomic classification using Kaiju with NCBI-nr database in the seawater (SW), oil contaminated seawater (SWO) and biostimulated oil contaminated seawater (SWOB).

Table S8. Major bacterial phyla proportions based on taxonomic classification using Kaiju with MAR_{DB} database in the seawater (SW), oil contaminated seawater (SWO) and biostimulated oil contaminated seawater (SWOB).

Table S9. Major bacterial phyla proportions based on taxonomic classification using Kraken2 with Standard Kraken2 database in the seawater (SW), oil contaminated seawater (SWO) and biostimulated oil contaminated seawater (SWOB).

Table S10. Major bacterial phyla proportions based on taxonomic classification using Bracken with Standard Kraken2 database in the seawater (SW), oil contaminated seawater (SWO) and biostimulated oil contaminated seawater (SWOB).

Table S11. The mean proportions and standard deviations of major bacterial phyla based on taxonomic classification using amplicon-based sequencing with SILVA database in the seawater (SW), oil contaminated seawater (SWO) and biostimulated oil contaminated seawater (SWOB).

Table S12. The proportions of the 50 predominant bacterial genera across all samples in seawater (SW), oil contaminated seawater (SWO), and biostimulated oil contaminated seawater (SWOB) according to taxonomic classification using Kaiju with the NCBI-nr database.

Table S13. The proportions of the 50 predominant bacterial genera across all samples in seawater (SW), oil contaminated seawater (SWO), and biostimulated oil contaminated seawater (SWOB) according to taxonomic classification using Kaiju with the MAR_{DB} database.

Table S14. The proportions of the 50 predominant bacterial genera across all samples in seawater (SW), oil contaminated seawater (SWO), and biostimulated oil contaminated seawater (SWOB) according to taxonomic classification using Kraken2 with the Standard Kraken2 database.

Table S15. The proportions of the 50 predominant bacterial genera across all samples in seawater (SW), oil contaminated seawater (SWO), and biostimulated oil contaminated seawater (SWOB) according to taxonomic classification using Bracken with the Standard Kraken2 database.

Table S16. The mean proportions and standard deviations of the 50 predominant bacterial genera across all samples in seawater (SW), oil contaminated seawater (SWO), and biostimulated oil contaminated seawater (SWOB) according to taxonomic classification based on amplicon-sequencing and the SILVA database.

Table S17. The correlation between datasets of proportions of the 50 predominant bacterial genera and proportions of bacterial genera containing hydrocarbon degraders acquired with five different taxonomic classification methods.

Table S18. The proportions of the most abundant bacterial species (>1% of bacterial community in at least one treatment) in seawater (SW), oil contaminated seawater (SWO), and biostimulated oil contaminated seawater (SWOB).

Table S19. The number of detected genera, proportions and estimated abundances of bacterial genera involving petroleum hydrocarbon degraders in seawater (SW), oil contaminated seawater (SWO), and biostimulated oil contaminated seawater (SWOB) based on five taxonomic assignment methods.

Table S20. Normalized hydrocarbon degradation related gene abundances (RPKG) in seawater (SW), oil-contaminated seawater (SWO), and biostimulated oil-contaminated seawater (SWOB).

Table S21. Average nucleotide identity (ANI) score-based clustering of good quality (completeness >50%, contamination <10%) metagenome assembled genomes (MAGs) and their oil degradation gene profiles.

Table S22. Major operational taxonomic units (OTUs) of bacterial and archaeal genera found in seawater (SW), oil contaminated seawater (SWO), and biostimulated oil contaminated seawater (SWOB) after four and eight months of incubation according to amplicon-based sequencing.

Supplementary figures

Fig. S1. The abundances of bacterial (A) and archaeal (B) communities, as well as the relative abundance of the archaeal community (C) in seawater (SW), oil contaminated seawater (SWO) and biostimulated oil contaminated seawater (SWOB).

Fig. S2. Venn diagram showing the overlap of detected genera containing oil hydrocarbon degraders between different taxonomic classification methods.

Fig. S3. The clustering of seawater (SW), oil contaminated seawater (SWO) and biostimulated oil contaminated seawater (SWOB) microcosms based on the crl-transformed proportions of the 50 predominant genera of Kaiju (A), Kaiju/MAR (B), Kraken2 (C), Bracken (D), and Amplicon (E) taxonomic classifications.

Fig. S4. Multiple co-inertia analysis results based on datasets of the top 50 bacterial genera proportions based on five taxonomic classification methods.

Fig. S5. Multiple co-inertia analysis results based on datasets of proportions of bacterial genera containing oil hydrocarbon degraders based on five taxonomic classification methods.

Fig. S6. The bacterial community structure at phylum, genus (>3%), and species (>3%) level in seawater (SW), oil-contaminated seawater (SWO), and biostimulated oil-contaminated seawater (SWOB).

Fig. S7. The proportions of archaeal phyla as well as the most dominant archaeal genera and species in the archaeal community according to Kaiju in seawater (SW), oil contaminated seawater (SWO) and biostimulated oil contaminated seawater (SWOB).

Supplementary texts

Section S1: Prokaryotic Community Structure in Different Treatments According to Kaiju.

Table S1. The means and standard deviations (in parenthesis, $n = 2$) of physicochemical characteristics of the seawater (SW), oil contaminated seawater (SWO) and biostimulated oil contaminated seawater (SWOB). The numbers in sample codes denote time in months. TOC – total organic carbon, N_{tot} – total nitrogen, P_{tot} – total phosphorous, NA – not analysed.

Sample	T (°C)	Salinity (ppt)	pH	O_2 (mg/L)	TOC (mg/L)	N_{tot} (mg/L)	NH_4-N (mg/L)	NO_3-N (mg/L)	P_{tot} (mg/L)
SW0	2.0 (0.2)	35.2 (0.3)	7.91 (0.04)	NA	2.20 (0.17)	<0.5	0.064 (0.014)	0.40 (0.10)	0.14 (0.02)
SW4	4.0 (0.1)	34.6 (1.5)	7.96 (0.20)	10.5 (0.5)	NA	<1.0	0.036 (0.002)	0.67 (0.03)	<0.1
SWO4	4.0 (0.1)	33.8 (1.4)	8.00 (0.20)	10.5 (0.5)	NA	<1.0	0.018 (0.001)	0.66 (0.03)	<0.1
SWOB4	4.0 (0.1)	35.2 (1.7)	6.82 (0.02)	8.3 (0.4)	18.3 (1.0)	445 (2.0)	255 (1.00)	42.8 (2.00)	152 (5.0)
SW8	4.0 (0.1)	35.0 (1.5)	8.23 (0.20)	11.8 (0.6)	NA	<0.5	0.033 (0.002)	<0.3	<0.1
SWO8	4.0 (0.1)	34.6 (1.5)	8.16 (0.20)	NA	NA	<0.5	0.010 (0.001)	<0.3	<0.1
SWOB8	4.0 (0.1)	36.0 (1.7)	6.85 (0.02)	10.6 (0.5)	18.0 (0.9)	487 (3.0)	262 (2.00)	22.0 (1.00)	105 (3.0)

Table S2. The characteristics of primer pairs and programs used in qPCR. LOQ – limit of quantification.

Target gene	Primers	Primer sequence 5´-3´	Amplicon size (bp)	Primer concentration (µM)	Amplification program	LOQ (copies/mL water)	Primer reference
Bacterial 16S rRNA	Bact517F	GCCAGCAGCCGCGGTAA	530	0.6	95°C 10 min; 35 cycles: 95°C 30 s; 60°C 45 s; 72°C 45 s[M]	50	[1]
	Bact1028R	CGACARCCATGCASCACCT					[2]
Archaeal 16S rRNA	Arc519F	CAGYCGCCRCGGTAA	393	0.6	95°C 10 min; 35 cycles: 95°C 15 s; 56°C 30 s; 72°C 30 s[M]	50	[3]
	Arch910R	GCYCCCCCGCCWATTC					
Colwellia 16S rRNA	Col134F	CCTTATGGTGGGGGACAACA	96	0.6	95°C 10 min; 35 cycles: 95°C 15 s; 56°C 30 s; 72°C 30 s[M]	25	Modified from [4]
	Col209Rm	AATCAAATGGCGARAGGTCC					
Cycloclasticus 16S rRNA	Cyc467F	AACCTTAGGCCCTGACGT	128	0.6	95°C 10 min; 40 cycles: 95°C 15 s; 54°C 30 s; 72°C 30 s[M]	25	[5]
	Cyc577R	TGTTTAACCGCCTACGCG					
Pseudomonas 16S rRNA	Ps-for	GGTCTGAGAGGATGATCAGT	990	0.6	95°C 10 min; 40 cycles: 95°C 30 s; 55°C 45 s; 72°C 45 s[M]	25	[6]
	Ps-rev	TTAGCTCCACCTCGCGGC					

[M] Immediately after the qPCR assay melting curve analysis was performed by increasing the temperature from 70 °C to 90 °C (0.35 °C/3 s) with continuous fluorescence recording.

Table S3. The number of total reads, reads after quality trimming, coverage and diversity metrics, the proportions of classified reads according to different taxonomic assignment methods (%), and the numbers of contigs in the metagenomes of seawater (SW), oil contaminated seawater (SWO) and biostimulated oil contaminated seawater (SWOB). The numbers in sample codes denote time in months.

Sample	Number of total reads	Number of reads after quality trimming	Coverage	Diversity	Classified reads (%)				Contigs
					Kaiju	Kaiju/MAR	Kraken2	Bracken	
SW0	61252004	60751670	0.774	18.85	67.6	74.5	18.0	18.0	215103 (453660249 bp)
SW4	58800661	58762069	0.968	17.55	72.7	71.3	25.1	25.0	78017 (232006212 bp)
SWO4	62447105	62411054	0.990	17.87	88.3	85.3	46.6	42.6	66796 (217880730 bp)
SWOB4	55743647	54954391	0.998	17.26	92.2	87.2	62.1	62.1	23475 (113304343 bp)
SW8	61429147	60960320	0.968	18.29	78.2	72.1	27.0	27.0	104532 (377477043 bp)
SWO8	57527324	57467303	0.992	17.50	77.6	79.3	37.6	37.6	67393 (205560027 bp)
SWOB8	60566394	60529898	1.000	16.37	91.0	84.4	39.1	39.1	19588 (97474974 bp)

Table S4. The number of obtained good quality total, bacterial (Bact) and archaeal (Arch) 16S rRNA gene sequences of amplicon-based sequencing, as well as the coverage estimates, number of operational taxonomic units (OTUs) and diversity indices (1/S - Inverse Simpson index, SH – Shannon diversity index) of bacterial community in the seawater (SW), oil contaminated seawater (SWO) and biostimulated oil contaminated seawater (SWOB). The first number in sample codes denotes time in months and the second number denotes treatment parallel.

Sample	No of sequences			Coverage	No of OTUs	Diversity indices	
	Total	Bact	Arch	Bact	Bact	1/S Bact	SH Bact
SW0.1	42827	41033	1794	0.981	1365	12.38	4.27
SW0.2	43248	41105	2143	0.982	1312	12.52	4.27
SW4.1	45895	45837	58	0.979	1280	19.48	4.21
SW4.2	25449	25375	74	0.976	1603	30.93	4.52

Sample	No of sequences			Coverage	No of OTUs	Diversity indices	
	Total	Bact	Arch	Bact	Bact	1/S Bact	SH Bact
SWO4.1	30636	30527	109	0.974	1654	9.97	4.01
SWO4.2	74275	74156	119	0.980	1078	2.86	2.53
SWOB4.1	43632	43549	83	0.976	1520	10.38	3.81
SWOB4.2	37753	37691	62	0.977	1391	11.62	3.78
SW8.1	53601	53551	50	0.977	1501	3.69	3.20
SW8.2	54580	54539	41	0.977	1596	17.23	4.32
SWO8.1	80806	80680	126	0.977	1468	8.67	3.58
SWO8.2	106585	106443	142	0.978	1345	4.57	2.96
SWOB8.1	101981	101889	92	0.976	1701	15.37	4.16
SWOB8.2	54652	54617	35	0.977	1656	8.78	3.77

Table S5. The mean and standard deviations (in parenthesis, *n = 2*) of *n*-C17/Pristane and *n*-C18/Phytane ratios and concentrations (µg/L) of polyaromatic hydrocarbons (PAH) in sterilized oil contaminated seawater (SWO_S), oil contaminated seawater (SWO) and biostimulated oil contaminated seawater (SWOB). The numbers in sample codes denote time in months.

		SWO_S/SWO/ SWOB 0	SWO_S4	SWO_S8	SWO4	SWO8	SWOB4	SWOB8
	**Ratios**							
	n-C17/Pristane	0.25 (0.07)	0.25 (0.07)	0.20 (0.06)	0.25 (0.07)	0.20 (0.02)	0.20 (0.04)	0.10 (0.01)
	n-C18/Phytane	0.40 (0.14)	0.40 (0.14)	0.30 (0.10)	0.40 (0.14)	0.30 (0.10)	0.10 (0.02)	0 (0)
	**PAH concentrations**							
2-ring	Naphthalene	6465 (582)	2346.92 (211)	1092.98 (98)	1845.32 (166)	927.14 (83)	26.21 (2.4)	19.80 (2)
3-ring	Acenaphtylene	4.55 (0.4)	3.42 (0.3)	5.60 (0.5)	3.53 (0.3)	1.49 (0.1)	3.13 (0.3)	3.94 (0.4)
	Acenaphtene	355 (32)	132.53 (12)	163.85 (15)	137.96 (12)	174.47 (16)	150.72 (14)	96.78 (9)
	Fluorene	1169 (105)	528.19 (47.5)	525.85 (47)	544.00 (49)	602.74 54)	584.28 (53)	276.08 (25)
	Phenanthrene	1745 (105)	1383.49 (83)	1062.98 (64)	1333.38 (80)	1284.70 (77)	1644.95 (99)	675.81 (41)
	Anthracene	19.2 (1.2)	34.15 (2.0)	16.59 (1)	52.61 (3)	23.34 (1.4)	37.61 (2)	9.23 (0.6)
≥4-ring	Fluoranthene	152 (9.1)	135.87 (8.2)	103.86 (6)	150.62 (9)	110.05 (7)	143.58 (9)	106.17 (6)
	Pyrene	161 (6.4)	132.53 (5.3)	112.52 (4.5)	150.37 (6)	122.08 (5)	134.48 (5.4)	110.81 (4)
	Benzo(a)anthracene	30 (1.2)	14.46 (0.6)	14.29 (0.5)	11.82 (0.5)	24.39 (1)	28.23 (1)	24.87 (1)
	Chrysene	174 (7.0)	83.15 (3.3)	109.14 (4.4)	87.65 (3.5)	174.17 (7)	128.65 (5)	138.16 (5.5)

	SWO_S/SWO/ SWOB 0	SWO_S4	SWO_S8	SWO4	SWO8	SWOB4	SWOB8
Benzo(b)fluoranthene	50.9 (2.0)	34.14 (1.4)	30.83 (1)	31.39 (1.3)	45.85 (2)	43.31 (2)	48.05 (2)
Benzo(k)fluoranthene	8.35 (0.3)	6.06 (0.2)	6.96 (0.3)	5.40 (0.2)	10.04 (0.4)	6.96 (0.3)	6.35 (0.3)
Benzo(a)pyrene	30.2 (1.2)	20.29 (0.8)	17.88 (0.7)	19.75 (0.8)	21.23 (0.8)	26.10 (1)	19.55 (0.8)
Indeno(1,2,3-cd)pyrene	8.01 (0.3)	6.67 (0.3)	5.12 (0.2)	6.19 (0.2)	7.77 (0.3)	8.17 0.3)	8.48 (0.3)
Benzo(ghi)perylene	24.2 (1.0)	15.20 (0.6)	14.76 (0.6)	14.16 (0.6)	20.36 (0.8)	19.70 (0.8)	19.86 (0.8)
Dibenzo(a,h)antracene	5.99 (0.2)	4.26 (0.2)	4.02 (0.2)	3.03 (0.1)	6.31 (0.2)	4.39 (0.2)	6.01 (0.2)

Table S6. The mean abundances and standard deviations (in parenthesis, $n = 2$) of 16S rRNA genes specific to bacteria (B16S), archaea (A16S), *Colwellia*, *Cycloclasticus*, and *Pseudomonas* in the seawater (SW), oil contaminated seawater (SWO) and biostimulated oil contaminated seawater (SWOB). The numbers in sample codes denote time in months. NA – not analysed.

Target gene	SW0	SW4	SW8	SWO4	SWO8	SWOB4	SWOB8
B16S	1.02×10^6 (0.05×10^6)	3.72×10^6 (1.05×10^6)	5.23×10^6 (3.52×10^6)	2.90×10^6 (1.49×10^6)	4.59×10^6 (1.18×10^6)	8.28×10^7 (1.04×10^7)	1.24×10^8 (0.24×10^8)
A16S	1.83×10^5 (0.71×10^5)	1.89×10^4 (0.77×10^4)	3.37×10^4 (4.14×10^4)	5.46×10^4 (0.59×10^4)	1.01×10^5 (0.38×10^5)	2.63×10^4 (1.99×10^4)	3.35×10^4 (1.58×10^4)
Colwellia 16S rRNA	NA	NA	5.26×10^5 (6.79×10^5)	NA	6.01×10^5 (9.59×10^4)	6.68×10^5 (5.25×10^5)	4.53×10^5 (3.58×10^5)
Cycloclasticus 16S rRNA	NA	NA	9.51×10^3 (1.37×10^3)	NA	4.74×10^2 (1.07×10^2)	1.25×10^5 (1.75×10^5)	9.35×10^6 (1.07×10^6)
Pseudomonas 16S rRNA	NA	NA	1.64×10^5 (1.74×10^5)	NA	2.25×10^5 (3.29×10^3)	7.95×10^6 (2.10×10^6)	1.17×10^7 (4.27×10^6)

Table S7. Major (the 20 most abundant in each sample) bacterial phyla proportions (%) based on taxonomic classification using Kaiju with the NCBI-nr database in the seawater (SW), oil contaminated seawater (SWO) and biostimulated oil contaminated seawater (SWOB). The numbers in sample codes denote time in months.

Phylum	Class	SW0	SW4	SW8	SW04	SW08	SW0B4	SW0B8
Proteobacteria		82.33	79.08	69.84	73.33	76.82	90.30	94.52
	Alphaproteobacteria	19.82	11.15	14.20	36.06	18.57	6.75	16.72
	Betaproteobacteria	3.87	3.53	3.96	1.16	1.28	1.41	1.82

**Phylum**	**Class**	**SW0**	**SW4**	**SW8**	**SW04**	**SW08**	**SW0B4**	**SW0B8**
	Gammaproteobacteria	52.94	60.54	48.20	34.94	55.41	80.58	74.46
	Deltaproteobacteria	2.14	2.11	1.32	0.28	0.46	0.13	0.27
	Epsilonproteobacteria	0.19	0.22	0.19	0.08	0.26	0.06	0.11
Bacteroidetes		3.23	13.63	22.47	23.31	15.26	7.35	3.88
Planctomycetes		1.79	1.71	3.12	0.86	4.32	0.049	0.08
Firmicutes		1.98	2.38	1.22	1.20	0.97	1.76	0.71
Actinobacteria		2.90	1.35	1.06	0.56	0.99	0.31	0.40
Verrucomicrobia		2.13	0.18	0.49	0.10	0.21	0.02	0.03
Chloroflexi		1.75	0.19	0.19	0.07	0.13	0.02	0.05
Cyanobacteria		0.40	0.30	0.36	0.14	0.52	0.06	0.09
Acidobacteria		0.45	0.23	0.26	0.11	0.16	0.02	0.04
Gemmatimonadetes		0.51	0.06	0.06	0.02	0.05	<0.01	0.01
Nitrospinae		0.55	0.01	0.04	0.03	0.02	<0.01	0.01
Ca. Marinimicrobia		0.56	0.02	0.03	0.01	0.03	<0.01	<0.01
Spirochaetes		0.15	0.09	0.08	0.03	0.05	0.01	0.02
Nitrospirae		0.11	0.08	0.07	0.03	0.04	0.01	0.02
Balneolaeota		0.05	0.02	0.10	0.02	0.05	<0.01	0.01
Ca. Rokubacteria		0.08	0.04	0.04	0.01	0.01	<0.01	0.01
Ignavibacteriae		0.04	0.03	0.03	0.01	0.02	<0.01	0.01
Deinococcus-Thermus		0.04	0.03	0.03	0.01	0.02	<0.01	0.01
Calditrichaeota		0.06	0.03	0.03	0.01	0.02	<0.01	<0.01
Chlorobi		0.03	0.03	0.03	0.01	0.01	<0.01	0.01
Lentisphaerae		0.06	0.02	0.02	0.01	0.02	<0.01	<0.01
Chlamydiae		0.03	0.05	0.01	0.01	0.01	<0.01	<0.01
Ca. Omnitrophica		0.04	0.02	0.03	0.01	0.01	<0.01	<0.01
Ca. Tectomicrobia		0.06	0.01	0.02	0.01	0.01	<0.01	<0.01
Armatimonadetes		0.03	0.02	0.02	0.01	0.02	<0.01	<0.01
Tenericutes		0.02	0.06	0.02	<0.01	0.01	<0.01	<0.01
Ca. Dadabacteria		0.05	0.01	0.01	<0.01	0.01	<0.01	<0.01
Ca. Poribacteria		0.06	<0.01	<0.01	<0.01	<0.01	<0.01	<0.01
Aquificae		0.02	0.01	0.01	<0.01	0.01	<0.01	<0.01
Thermotogae		0.02	<0.01	<0.01	<0.01	0.01	<0.01	<0.01

Phylum	Class	SW0	SW4	SW8	SW04	SW08	SW0B4	SW0B8
Ca. Melainabacteria		0.01	0.01	0.01	<0.01	0.01	<0.01	<0.01
Deferribacteres		<0.01	<0.01	<0.01	<0.01	0.01	<0.01	<0.01

Table S8. Major (the 20 most abundant in each sample) bacterial phyla proportions (%) based on taxonomic classification using Kaiju with the MAR_{DB} database in the seawater (SW), oil contaminated seawater (SWO) and biostimulated oil contaminated seawater (SWOB). The numbers in sample codes denote time in months.

Phylum	Class	SW0	SW4	SW8	SW04	SW08	SW0B4	SW0B8
Proteobacteria		84.94	82.15	73.64	74.21	75.20	91.20	94.17
	Alphaproteobacteria	12.58	11.94	15.47	37.17	17.38	8.90	19.61
	Betaproteobacteria	0.53	1.31	2.45	0.31	0.30	0.70	1.30
	Gammaproteobacteria	67.20	64.65	51.17	35.19	55.77	79.54	70.35
	Deltaproteobacteria	2.01	2.16	1.11	0.30	0.34	0.29	0.54
	Epsilonproteobacteria	0.05	0.14	0.13	0.15	0.25	0.08	0.14
Bacteroidetes		2.51	13.30	19.95	22.67	14.46	7.90	4.27
Planctomycetes		1.96	1.74	3.77	1.58	8.70	0.09	0.18
Actinobacteria		1.59	0.96	0.50	0.42	0.36	0.33	0.51
Verrucomicrobia		2.26	0.36	0.62	0.11	0.21	0.05	0.09
Chloroflexi		2.22	0.19	0.17	0.07	0.10	0.04	0.08
Cyanobacteria		0.26	0.31	0.28	0.41	0.31	0.11	0.18
Ca. Marinimicrobia		1.42	0.09	0.11	0.04	0.10	0.02	0.04
Gemmatimonadetes		1.39	0.10	0.13	0.04	0.08	0.01	0.03
Firmicutes		0.15	0.28	0.22	0.10	0.13	0.12	0.20
Nitrospinae		0.69	0.03	0.05	0.04	0.03	0.01	0.01
Acidobacteria		0.20	0.07	0.07	0.13	0.06	0.01	0.02
Nitrospirae		0.04	0.06	0.06	0.02	0.02	0.01	0.04
Aquificae		0.04	0.02	0.04	0.03	0.05	0.03	0.03
Spirochaetes		0.06	0.05	0.05	0.02	0.02	0.01	0.02
Balneolaeota		0.01	0.02	0.07	0.01	0.04	0.01	0.01
Ca. Poribacteria		0.05	0.02	0.03	0.01	0.02	<0.01	<0.01
Ca. Tectomicrobia		0.02	0.02	0.02	0.01	0.01	0.01	0.01

Phylum	Class	SW0	SW4	SW8	SW04	SW08	SW0B4	SW0B8
Ignavibacteriae		0.01	0.02	0.03	0.01	0.01	<0.01	0.01
Lentisphaerae		0.02	0.02	0.02	0.01	0.01	<0.01	0.01
Kiritimatiellaeota		0.02	0.02	0.02	0.01	0.01	<0.01	0.01
Chlorobi		0.01	0.02	0.02	0.01	0.01	0.01	0.01
Thermodesulfobacteria		<0.01	0.01	0.01	0.01	0.02	0.01	0.01
Ca. Kaiserbacteria		0.01	0.01	0.01	0.01	0.01	<0.01	<0.01
Thermotogae		0.01	0.01	0.01	<0.01	0.01	<0.01	0.01

Table S9. Major (the 20 most abundant in each sample) bacterial phyla proportions (%) based on taxonomic classification using Kraken2 with the Standard Kraken2 database in the seawater (SW), oil contaminated seawater (SWO) and biostimulated oil contaminated seawater (SWOB). The numbers in sample codes denote time in months.

Phylum	Class	SW0	SW4	SW8	SW04	SW08	SW0B4	SW0B8
Proteobacteria		83.67	75.31	57.52	67.27	80.49	92.69	93.33
	Alphaproteobacteria	15.35	12.71	14.85	23.08	10.69	5.25	18.65
	Betaproteobacteria	3.39	4.21	3.21	2.14	4.69	1.21	3.89
	Gammaproteobacteria	61.06	54.69	36.42	39.85	63.18	85.12	67.87
	Deltaproteobacteria	0.46	0.62	0.44	0.24	0.16	0.08	0.28
	Epsilonproteobacteria	0.60	0.30	0.37	0.14	0.28	0.07	0.16
Bacteroidetes		4.31	15.64	33.66	28.10	15.91	4.43	3.87
Firmicutes		7.20	6.12	5.56	2.75	2.19	2.29	1.47
Actinobacteria		2.42	1.70	1.60	1.35	0.69	0.40	0.87
Cyanobacteria		0.92	0.50	0.40	0.18	0.24	0.09	0.19
Planctomycetes		0.50	0.23	0.76	0.14	0.26	0.02	0.07
Tenericutes		0.22	0.06	0.09	0.02	0.04	0.01	0.02
Spirochaetes		0.10	0.08	0.06	0.03	0.03	0.01	0.03
Verrucomicrobia		0.10	0.04	0.05	0.03	0.02	0.01	0.02
Fusobacteria		0.08	0.05	0.07	0.01	0.02	<0.01	0.01
Deinococcus-Thermus		0.04	0.06	0.04	0.03	0.01	0.01	0.02
Chloroflexi		0.07	0.05	0.03	0.01	0.02	0.01	0.02
Acidobacteria		0.07	0.03	0.03	0.02	0.01	0.01	0.02
Thermotogae		0.05	0.02	0.02	0.01	0.01	<0.01	0.01

Phylum	Class	SW0	SW4	SW8	SW04	SW08	SW0B4	SW0B8
Chlorobi		0.03	0.02	0.02	0.01	0.01	<0.01	0.02
Aquificae		0.05	0.01	0.01	<0.01	0.01	<0.01	<0.01
Chlamydiae		0.03	0.01	0.01	<0.01	0.01	<0.01	0.01
Gemmatimonadetes		0.02	0.01	0.01	0.01	<0.01	<0.01	<0.01
Thermodesulfobacteria		0.03	0.01	0.01	<0.01	0.01	<0.01	<0.01
Nitrospirae		0.01	0.01	0.01	0.01	0.01	<0.01	<0.01
Deferribacteres		0.01	0.01	0.01	<0.01	<0.01	<0.01	<0.01
Synergistetes		0.01	<0.01	<0.01	<0.01	<0.01	<0.01	<0.01
Chrysiogenetes		<0.01	<0.01	<0.01	<0.01	<0.01	<0.01	<0.01

Table S10. Major (the 20 most abundant in each sample) bacterial phyla proportions (%) based on taxonomic classification using Bracken with the Standard Kraken2 database in the seawater (SW), oil contaminated seawater (SWO) and biostimulated oil contaminated seawater (SWOB). The numbers in sample codes denote time in months.

Phylum	Class	SW0	SW4	SW8	SW04	SW08	SW0B4	SW0B8
Proteobacteria		81.90	75.88	57.04	67.21	79.90	92.40	93.18
	Alphaproteobacteria	14.44	12.08	14.41	22.53	10.55	5.16	18.41
	Betaproteobacteria	3.58	4.11	3.45	2.36	4.82	1.25	3.90
	Gammaproteobacteria	62.77	58.72	38.24	41.92	64.06	85.84	70.38
	Deltaproteobacteria	0.43	0.59	0.43	0.23	0.16	0.08	0.28
	Epsilonproteobacteria	0.57	0.29	0.37	0.14	0.28	0.07	0.16
Bacteroidetes		4.06	14.88	32.66	27.33	15.60	4.35	3.82
Firmicutes		9.36	6.41	7.08	3.60	3.10	2.66	1.70
Actinobacteria		2.34	1.64	1.57	1.32	0.69	0.40	0.87
Cyanobacteria		0.90	0.49	0.40	0.19	0.24	0.10	0.19
Planctomycetes		0.47	0.22	0.74	0.13	0.26	0.02	0.07
Tenericutes		0.21	0.06	0.08	0.02	0.04	0.01	0.02
Spirochaetes		0.09	0.07	0.06	0.03	0.03	0.01	0.02
Acidobacteria		0.13	0.03	0.03	0.02	0.02	0.01	0.02
Verrucomicrobia		0.09	0.03	0.05	0.03	0.02	0.01	0.02
Fusobacteria		0.08	0.05	0.06	0.01	0.02	<0.01	0.01
Deinococcus-Thermus		0.04	0.06	0.04	0.03	0.01	0.01	0.02

Phylum	Class	SW0	SW4	SW8	SW04	SW08	SW0B4	SW0B8
Chloroflexi		0.07	0.04	0.03	0.01	0.01	<0.01	0.02
Thermotogae		0.05	0.03	0.04	0.01	0.01	<0.01	0.01
Chlorobi		0.02	0.02	0.02	0.01	0.01	<0.01	0.02
Aquificae		0.05	0.01	0.01	<0.01	0.01	<0.01	<0.01
Chlamydiae		0.03	0.01	0.01	<0.01	0.01	<0.01	0.01
Gemmatimonadetes		0.02	0.01	0.01	0.01	<0.01	<0.01	<0.01
Thermodesulfobacteria		0.02	0.01	0.01	<0.01	0.01	<0.01	<0.01
Nitrospirae		0.01	0.01	0.01	0.01	0.01	<0.01	<0.01
Deferribacteres		0.01	0.01	0.01	<0.01	<0.01	<0.01	<0.01
Synergistetes		0.01	<0.01	<0.01	<0.01	<0.01	<0.01	<0.01
Chrysiogenetes		<0.01	<0.01	<0.01	<0.01	<0.01	<0.01	<0.01

Table S11. The mean proportions and standard deviations (in parenthesis, n = 2) of major (the 20 most abundant in each sample) bacterial phyla based on taxonomic classification using amplicon-based sequencing with the SILVA database in the seawater (SW), oil contaminated seawater (SWO) and biostimulated oil contaminated seawater (SWOB). The numbers in sample codes denote time in months.

Phylum	Class	SW0	SW4	SW8	SWO4	SWO8	SWOB4	SWOB8
Proteobacteria		61.02 (0.88)	53.27 (8.10)	43.64 (30.00)	29.71 (12.31)	32.43 (6.41)	59.89 (7.65)	62.16 (1.58)
	Alphaproteobacteria	13.48 (0.37)	24.10 (11.29)	20.24 (21.09)	15.33 (4.30)	7.56 (1.36)	13.38 (0.26)	15.88 (6.28)
	Gammaproteobacteria	45.97 (0.61)	23.00 (12.00)	20.13 (7.88)	13.82 (7.76)	24.25 (7.63)	45.68 (7.76)	45.02 (7.89)
	Deltaproteobacteria	1.27 (0.07)	5.85 (7.21)	2.82 (0.80)	0.34 (0.14)	0.38 0.11)	0.43 (0.03)	0.78 (0.02)
Bacteroidetes		13.30 (1.58)	35.02 (12.55)	38.63 (39.52)	62.72 (16.69)	53.84 (3.39)	33.48 (7.31)	27.13 (1.60)
Cyanobacteria		14.68 (2.51)	3.88 (1.14)	0.58 (0.23)	2.28 (1.41)	0.16 (0.11)	2.59 (0.25)	0.17 (0.03)
Chloroflexi		1.54 (0.12)	0.36 (0.22)	0.51 (0.08)	0.34 (0.23)	0.32 (0.05)	0.28 (0.03)	0.59 (0.01)
Marinimicrobia		1.62	0.11	0.02	0.08	0.04	0.09	0.02

Phylum	Class	SW0	SW4	SW8	SWO4	SWO8	SWOB4	SWOB8
		(0.02)	(0.05)	(0.02)	(0.05)	(0.02)	(<0.01)	(<0.01)
Verrucomicrobia		1.56<br>(0.02)	0.86<br>(0.36)	1.74<br>(1.06)	0.57<br>(0.33)	0.67<br>(0.15)	0.62<br>(0.07)	1.24<br>(0.01)
Planctomycetes		1.47<br>(0.12)	2.95<br>(0.73)	9.60<br>(7.78)	1.69<br>(0.67)	9.14<br>(10.89)	0.57<br>(0.06)	1.91<br>(<0.00)
Acidobacteria		1.10<br>(0.23)	1.32<br>(0.80)	2.88<br>(0.38)	1.07<br>(0.69)	1.85<br>(0.40)	1.21<br>(0.03)	2.92<br>(0.03)
Nitrospinae		1.01<br>(0.01)	0.09<br>(0.06)	0.02<br>(0.02)	0.08<br>(<0.01)	0.02<br>(<0.01)	0.05<br>(<0.01)	0.01<br>(<0.01)
Actinobacteria		0.61<br>(0.05)	0.67<br>(0.22)	0.83<br>(0.07)	0.47<br>(0.22)	0.52<br>(0.12)	0.46<br>(0.11)	1.15<br>(0.03)
Dadabacteria		0.42<br>(0.06)	0.02<br>(0.01)	<0.01<br>(<0.01)	0.02<br>(0.01)	0.01<br>(<0.01)	0.01<br>(<0.01)	<0.01<br>(<0.01)
Gemmatimonadetes		0.42<br>(<0.01)	0.28<br>(0.18)	0.32<br>(0.04)	0.20<br>(0.14)	0.21<br>(0.03)	0.18<br>(0.02)	0.46<br>(<0.01)
Firmicutes		0.66<br>(0.48)	0.70<br>(0.47)	0.41<br>(0.10)	0.43<br>(0.46)	0.24<br>(0.09)	0.23<br>(0.04)	1.34<br>(0.01)
PAUC34f		0.15<br>(0.04)	0.02<br>(<0.01)	0.00<br>(0.00)	0.01<br>(<0.01)	<0.01<br>(<0.01)	0.01<br>(<0.01)	<0.01<br>(<0.01)
Lentisphaerae		0.07<br>(<0.01)	0.02<br>(0.01)	<0.01<br>(<0.01)	0.02<br>(<0.01)	0.01<br>(0.01)	0.01<br>(0.01)	<0.01<br>(<0.01)
Margulisbacteria		0.05<br>(<0.01)	0.01<br>(<0.01)	0.00<br>(0.00)	<0.01<br>(<0.01)	<0.01<br>(<0.01)	0.00<br>(0.00)	<0.01<br>(<0.01)
AncK6		0.04<br>(<0.01)	0.00<br>(0.00)	0.00<br>(0.00)	<0.01<br>(<0.01)	0.00<br>(0.00)	<0.01<br>(<0.01)	0.00<br>(0.00)
Latescibacteria		0.03<br>(0.01)	0.07<br>(0.04)	0.14<br>(<0.01)	0.05<br>(0.01)	0.08<br>(0.03)	0.05<br>(0.01)	0.18<br>(0.01)
Rokubacteria		0.02<br>(0.01)	0.04<br>(0.02)	0.09<br>(0.02)	0.05<br>(0.03)	0.06<br>(<0.01)	0.04<br>(0.01)	0.13<br>(0.01)
Spirochaetes		0.04<br>(0.01)	0.02<br>(0.01)	0.06<br>(0.01)	0.02<br>(0.02)	0.04<br>(<0.01)	0.01<br>(0.01)	0.09<br>(0.01)
Armatimonadetes		0.02	0.03	0.06	0.01	0.03	0.04	0.07

Phylum	Class	SW0	SW4	SW8	SWO4	SWO8	SWOB4	SWOB8
		(0.01)	(0.01)	(0.01)	(0.01)	(0.01)	(0.01)	(<0.01)
Patescibacteria		0.01 (0.01)	0.02 (<0.01)	0.01 (<0.01)	0.02 (0.02)	0.01 (<0.01)	0.01 (0.01)	0.02 (<0.01)
Nitrospirae		0.02 (0.01)	0.04 (0.03)	0.08 (0.01)	0.02 (0.00)	0.04 (0.01)	0.03 (0.01)	0.08 (0.02)
Elusimicrobia		0.02 (0.01)	0.04 (0.03)	0.05 (0.01)	0.03 (0.02)	0.04 (0.02)	0.03 (0.01)	0.07 (<0.01)
Epsilonbacteraeota		0.01 (<0.01)	0.02 (<0.01)	0.01 (0.01)	0.03 (0.01)	0.11 (0.03)	0.03 (0.01)	0.03 (<0.01)
Omnitrophicaeota		0.02 (0.01)	0.02 (0.01)	0.03 (<0.01)	0.02 (0.01)	0.01 (0.01)	0.01 (0.01)	0.02 (<0.01)
Synergistetes		0.01 (<0.01)	0.01 (0.01)	0.02 (<0.01)	0.01 (0.01)	0.02 (0.01)	0.01 (0.01)	0.02 (<0.01)
Fibrobacteres		0.01 (<0.01)	<0.01 (0.01)	0.02 (<0.01)	0.01 (<0.01)	0.01 (0.01)	0.01 (0.01)	0.02 (<0.01)
Thermotogae		0.01 (0.01)	0.01 (<0.01)	0.02 (0.01)	0.01 (0.01)	0.02 (<0.01)	0.01 (<0.01)	0.02 (<0.01)
WPS-2		0.01 (<0.01)	<0.01 (<0.01)	0.12 (0.15)	<0.01 (<0.01)	0.01 (0.01)	0.01 (<0.01)	0.01 (<0.01)
Entotheonellaeota		0.00 (0.00)	0.01 (<0.01)	0.01 (<0.01)	0.01 (0.01)	0.01 (<0.01)	0.01 (<0.01)	0.02 (<0.01)

Table S12. The proportions (%) of the 50 predominant bacterial genera across all samples in seawater (SW), oil contaminated seawater (SWO), and biostimulated oil contaminated seawater (SWOB) according to taxonomic classification using Kaiju with the NCBI-nr database. The numbers in sample codes denote time in months.

Genus	SW0	SW4	SW8	SWO4	SWO8	SWOB4	SWOB8
Pseudomonas	1.96	1.23	1.97	9.64	6.42	38.41	25.39
Cycloclasticus	0.13	10.05	8.81	1.10	2.12	1.82	13.36
Marinomonas	1.10	1.71	0.25	5.77	11.68	12.82	4.29
Colwellia	3.96	4.94	3.86	4.57	10.87	1.73	0.60
Paraperlucidibaca	0.02	0.01	0.01	<0.01	<0.01	3.38	14.57

Genus	SW0	SW4	SW8	SWO4	SWO8	SWOB4	SWOB8
Hyphomonas	0.03	0.10	0.17	16.33	4.96	0.02	0.08
Sphingorhabdus	0.02	0.65	0.28	0.27	0.03	2.12	5.05
Polaribacter	0.26	1.38	4.55	1.51	1.30	0.68	0.06
Pacificibacter	0.03	0.87	0.83	4.51	2.32	0.06	0.19
Paraglaciecola	0.18	0.47	1.05	0.65	2.35	1.66	0.94
Ulvibacter	0.02	0.33	0.32	1.50	1.02	3.43	1.05
Methylophaga	0.14	1.31	2.81	0.89	0.62	0.14	0.17
Maribacter	0.03	1.16	0.71	3.21	1.13	0.06	0.05
Ca Thioglobus	4.14	0.05	0.09	0.03	0.33	0.01	0.02
Marinobacter	0.68	0.42	0.27	0.41	0.23	1.97	0.56
Clostridioides	0.53	1.32	0.35	0.36	0.26	1.16	0.31
Oleispira	0.17	0.09	0.18	0.02	0.10	4.35	0.03
Halomonas	0.82	0.32	0.19	0.17	1.67	0.24	0.25
Perlucidibaca	0.05	0.02	0.01	0.00	0.01	0.51	2.15
Neptunomonas	0.87	0.16	0.11	0.60	0.17	2.04	0.09
Ca Pelagibacter	1.70	0.10	1.22	0.01	0.16	0.01	<0.01
Tenacibaculum	0.04	0.60	2.26	0.28	0.14	0.08	0.02
Aequorivita	0.01	0.13	0.19	0.19	0.47	1.03	0.92
Winogradskyella	0.02	0.65	0.56	1.26	0.45	0.07	0.13
Nisaea	0.02	0.01	2.74	0.01	0.01	0.01	0.04
Amphritea	0.62	0.11	0.06	0.73	0.17	1.22	0.17
Lacinutrix	0.01	0.15	0.29	1.26	1.12	0.04	0.06
Sphingopyxis	0.03	0.13	0.07	0.19	0.04	0.48	1.11
Marinobacterium	1.09	0.21	0.12	0.06	0.18	0.08	0.06
Parvibaculum	0.01	0.01	0.01	1.35	0.84	<0.01	<0.01
Marinirhabdus	<0.01	0.03	0.10	0.09	1.84	0.01	0.01
Flavobacterium	0.09	0.38	0.88	0.56	0.37	0.14	0.08
Pseudoalteromonas	0.47	0.51	0.30	0.19	0.42	0.20	0.13
Shewanella	0.46	0.32	0.26	0.14	0.28	0.83	0.12
Hoeflea	0.04	0.11	0.19	0.64	0.28	0.04	0.31
Doktonia	<0.01	0.36	0.90	0.06	0.09	0.02	0.01
Lutibacter	0.02	0.24	0.82	0.18	0.08	0.08	0.03
Streptomyces	0.31	0.31	0.19	0.09	0.39	0.06	0.05

Genus	SW0	SW4	SW8	SWO4	SWO8	SWOB4	SWOB8
Neptuniibacter	0.34	0.14	0.10	0.04	0.08	0.07	0.37
Photobacterium	0.24	0.10	0.08	0.04	0.64	0.05	0.09
Ralstonia	0.20	0.34	0.06	0.19	0.04	0.47	0.03
Motiliproteus	0.75	0.07	0.04	0.03	0.11	0.04	0.02
Maricaulis	0.01	0.37	0.26	0.55	0.02	<0.01	0.01
Acinetobacter	0.27	0.17	0.10	0.05	0.09	0.09	0.25
Microbulbifer	0.22	0.44	0.19	0.04	0.07	0.03	0.06
Thalassotalea	0.35	0.12	0.15	0.09	0.26	0.04	0.02
Sphingomonas	0.13	0.10	0.09	0.13	0.09	0.09	0.27

Table S13. The proportions (%) of the 50 predominant bacterial genera across all samples in seawater (SW), oil contaminated seawater (SWO), and biostimulated oil contaminated seawater (SWOB) according to taxonomic classification using Kaiju with the MAR_{DB} database. The numbers in sample codes denote time in months.

Genus	SW0	SW4	SW8	SW04	SW08	SW0B4	SW0B8
Cycloclasticus	0.25	23.20	16.69	1.75	3.99	2.66	25.33
Pseudomonas	0.48	0.94	2.24	8.59	9.44	39.03	13.88
Marinomonas	0.91	3.37	0.40	7.90	18.88	16.11	8.05
Colwellia	17.78	8.46	5.18	4.90	13.63	1.86	1.08
Hyphomonas	0.33	0.22	0.29	21.67	8.46	0.05	0.19
Flavobacterium	0.36	5.92	18.51	0.50	0.28	0.13	0.13
Sphingorhabdus	0.05	1.01	0.36	0.30	0.04	2.04	6.98
Paraglaciecola	0.83	1.11	1.90	0.98	4.12	2.20	1.89
Pacificibacter	0.05	1.64	1.26	6.55	4.01	0.08	0.38
Methylophaga	0.34	3.41	6.01	1.62	1.33	0.28	0.53
Ulvibacter	0.04	0.42	0.29	2.05	0.62	4.55	2.07
Ca. Pelagibacter	7.95	0.19	2.05	0.02	0.19	0.01	0.01
Marinobacter	0.85	1.00	0.52	0.71	0.39	2.87	1.94
Maribacter	0.14	2.18	1.00	4.31	1.85	0.10	0.11
Vibrio	2.95	1.08	0.88	0.54	1.01	0.74	0.81
Polaribacter	1.67	0.83	1.31	1.92	1.96	0.84	0.12
Sphingopyxis	0.06	0.32	0.16	0.37	0.07	0.99	3.19

Genus	SW0	SW4	SW8	SW04	SW08	SW0B4	SW0B8
Oleispira	0.29	0.24	0.32	0.04	0.17	5.57	0.13
Halomonas	0.34	0.41	0.22	0.25	2.73	0.53	0.76
Neptunomonas	0.40	0.41	0.23	0.98	0.26	2.99	0.36
Pseudoalteromonas	1.51	1.24	0.66	0.38	0.85	0.39	0.47
Sneathiella	0.09	0.08	0.06	0.67	0.62	0.82	1.68
Nisaea	0.19	0.03	5.14	0.03	0.04	0.02	0.10
Lacinutrix	0.08	0.31	0.33	2.25	2.14	0.09	0.19
Alcanivorax	0.21	0.60	0.26	0.16	0.12	0.42	1.60
Stappia	0.09	0.08	0.12	0.78	0.09	0.81	1.49
Sulfitobacter	0.54	1.11	1.02	0.55	0.60	0.08	0.13
Nitrospina	4.43	0.04	0.06	0.05	0.04	0.01	0.02
Shewanella	0.86	0.65	0.47	0.24	0.45	1.11	0.40
Aequorivita	0.05	0.21	0.22	0.22	0.40	1.11	1.36
Zhongshania	0.03	0.21	3.21	0.02	0.02	0.03	0.11
Hoeflea	0.15	0.31	0.36	0.97	0.50	0.06	0.64
Winogradskyella	0.06	0.80	0.34	1.03	0.43	0.06	0.16
Amphritea	0.20	0.18	0.08	0.77	0.13	1.15	0.26
Donghicola	1.20	0.47	0.26	0.20	0.55	0.10	0.04
Parvibaculum	0.16	0.03	0.07	1.39	1.03	0.00	0.02
Microbulbifer	0.28	1.00	0.34	0.09	0.11	0.11	0.33
Marinobacterium	0.38	0.45	0.22	0.18	0.15	0.30	0.40
Porticoccus	1.11	0.76	0.45	0.02	0.03	0.02	0.05
Loktanella	1.35	0.22	0.14	0.09	0.11	0.06	0.25
Neptuniibacter	0.18	0.33	0.19	0.08	0.08	0.14	0.74
Maricaulis	0.18	0.80	0.48	0.85	0.07	0.01	0.03
Halobacteriovorax	0.08	0.10	2.22	0.01	0.01	0.00	0.01
Ca. Thioglobus	2.27	0.03	0.03	0.01	0.05	0.00	0.01
Thalassotalea	1.05	0.25	0.24	0.15	0.45	0.06	0.07
Psychromonas	0.65	0.40	0.28	0.16	0.35	0.15	0.15
Acinetobacter	0.12	0.06	0.04	0.02	0.03	0.20	0.88
Endozoicomonas	0.60	0.46	0.21	0.07	0.13	0.14	0.29
Alteromonas	0.53	0.37	0.23	0.10	0.20	0.11	0.30
Streptomyces	0.43	1.00	0.16	0.08	0.07	0.10	0.15

Table S14. The proportions (%) of the 50 predominant bacterial genera across all samples in seawater (SW), oil contaminated seawater (SWO), and biostimulated oil contaminated seawater (SWOB) according to taxonomic classification using Kraken2 with the Standard Kraken2 database. The numbers in sample codes denote time in months.

Genus	SW0	SW4	SW8	SW04	SW08	SW0B4	SW0B8
Pseudomonas	3.02	5.68	5.37	13.63	13.06	50.54	34.31
Marinomonas	2.24	5.49	0.62	13.10	25.14	19.14	10.36
Colwellia	8.32	9.97	6.26	4.95	10.82	1.39	0.61
Sphingorhabdus	0.04	2.68	0.96	0.76	0.09	3.53	12.40
Polaribacter	1.36	5.13	15.15	5.14	3.35	1.32	0.34
Staphylococcus	4.46	5.20	4.46	2.26	1.42	2.03	0.89
Cycloclasticus	0.18	1.90	1.47	0.14	0.23	0.21	4.96
Ca. Pelagibacter	7.83	0.23	4.15	0.02	0.20	0.01	0.01
Shewanella	3.14	1.80	1.45	0.59	1.06	1.56	0.93
Klebsiella	0.66	3.01	0.32	0.31	0.16	3.90	1.98
Pseudoalteromonas	3.36	1.94	1.42	0.70	1.47	0.47	0.67
Halomonas	1.19	1.14	0.62	0.93	3.80	0.71	1.40
Acinetobacter	2.96	1.10	1.06	0.31	0.51	0.35	1.88
Lacinutrix	0.06	0.56	1.22	3.67	3.09	0.14	0.21
Vibrio	3.15	1.48	1.24	0.57	0.92	0.41	0.97
Winogradskyella	0.09	1.52	1.59	2.53	1.03	0.15	0.26
Flavobacterium	0.38	1.09	2.07	1.99	0.88	0.37	0.33
Sulfitobacter	0.71	1.28	1.25	1.57	1.34	0.12	0.26
Paraglaciecola	0.61	0.64	1.17	0.53	1.63	0.60	0.60
Ca. Thioglobus	6.31	0.08	0.11	0.03	0.23	0.01	0.03
Marinobacter	0.55	0.87	0.44	0.40	0.16	1.58	0.88
Hyphomonas	0.02	0.06	0.09	4.26	1.11	0.01	0.05
Tenacibaculum	0.15	0.96	3.04	0.79	0.32	0.12	0.09
Neptunomonas	1.25	0.24	0.16	0.95	0.12	2.14	0.17
Maribacter	0.07	0.56	0.68	2.33	0.74	0.06	0.05
Ralstonia	0.06	0.06	0.05	0.07	3.89	0.03	0.09
Formosa	0.09	0.53	1.11	1.71	0.59	0.11	0.18
Alteromonas	1.09	0.50	0.44	0.18	0.36	0.14	0.34
Zhongshania	0.12	0.90	2.01	0.01	0.02	0.01	0.09

Genus	SW0	SW4	SW8	SW04	SW08	SW0B4	SW0B8
Cellulophaga	0.07	0.47	0.80	1.09	0.31	0.11	0.09
Aequorivita	0.02	0.11	0.19	0.22	0.35	0.47	0.70
Algibacter	0.04	0.27	0.48	1.22	0.40	0.11	0.13
Sphingomonas	0.13	0.22	0.21	0.58	0.22	0.14	0.61
Salmonella	0.63	0.74	0.21	0.33	0.15	0.23	0.22
Rhizobium	0.26	0.24	0.34	0.76	0.38	0.07	0.30
Bradyrhizobium	0.26	0.25	0.33	0.70	0.31	0.05	0.25
Microbulbifer	0.38	1.01	0.38	0.10	0.08	0.06	0.21
Legionella	0.78	0.45	0.37	0.08	0.14	0.04	0.22
Celeribacter	0.15	0.48	0.43	0.62	0.29	0.02	0.08
Mesorhizobium	0.21	0.19	0.29	0.62	0.30	0.04	0.23
Haemophilus	0.77	0.43	0.21	0.20	0.18	0.12	0.12
Streptomyces	0.56	0.37	0.38	0.31	0.11	0.09	0.14
Octadecabacter	0.74	0.41	0.32	0.33	0.17	0.01	0.07
Thalassotalea	0.60	0.28	0.29	0.19	0.43	0.05	0.11
Psychrobacter	0.67	0.28	0.19	0.05	0.09	0.07	0.34
Olleya	0.02	0.15	0.28	0.92	0.29	0.04	0.08
Xanthomonas	0.31	0.43	0.19	0.23	0.09	0.20	0.22
Cellvibrio	0.29	0.72	0.29	0.05	0.07	0.05	0.21
Aquimarina	0.06	0.33	0.70	0.37	0.18	0.08	0.06
Burkholderia	0.32	0.37	0.20	0.28	0.10	0.14	0.22

Table S15. The proportions (%) of the 50 predominant bacterial genera across all samples in seawater (SW), oil contaminated seawater (SWO), and biostimulated oil contaminated seawater (SWOB) according to taxonomic classification using Bracken with the Standard Kraken 2 database. The numbers in sample codes denote time in months.

Genus	SW0	SW4	SW8	SW04	SW08	SW0B4	SW0B8
Pseudomonas	2.71	4.74	4.83	12.21	11.97	48.14	31.65
Marinomonas	1.94	4.51	0.54	11.32	22.82	18.07	9.26
Colwellia	6.73	8.16	5.34	4.33	10.05	1.33	0.54
Klebsiella	0.80	11.87	0.51	0.38	0.24	5.58	6.71
Sphingorhabdus	0.03	2.19	0.81	0.65	0.08	3.35	11.23

Genus	SW0	SW4	SW8	SW04	SW08	SW0B4	SW0B8
Polaribacter	1.15	4.48	13.76	4.71	3.25	1.34	0.33
Salmonella	8.57	2.66	3.95	3.44	2.46	1.36	1.30
Staphylococcus	5.00	4.96	5.22	2.76	1.91	2.30	1.01
Pseudoalteromonas	4.81	2.29	2.05	1.27	1.97	0.67	0.85
Cycloclasticus	0.14	1.55	1.25	0.12	0.21	0.20	4.34
Halomonas	1.02	0.94	0.55	0.81	3.57	0.68	1.25
Ca. Pelagibacter	6.16	0.19	3.44	0.02	0.18	0.01	0.00
Shewanella	2.54	1.49	1.30	0.52	1.01	1.48	0.83
Acinetobacter	2.55	0.93	1.00	0.28	0.50	0.35	1.69
Lacinutrix	0.06	0.54	1.23	3.50	3.04	0.16	0.22
Vibrio	2.82	1.22	1.12	0.50	0.88	0.40	0.87
Winogradskyella	0.08	1.46	1.63	2.47	1.05	0.17	0.28
Flavobacterium	0.34	0.99	1.97	1.86	0.86	0.39	0.33
Sulfitobacter	0.66	1.20	1.18	1.47	1.30	0.12	0.26
Paraglaciecola	0.49	0.53	1.00	0.46	1.52	0.58	0.55
Ca. Thioglobus	4.98	0.07	0.15	0.03	0.21	0.01	0.03
Hyphomonas	0.02	0.05	0.08	4.08	1.11	0.01	0.05
Tenacibaculum	0.14	0.88	2.91	0.75	0.31	0.13	0.09
Marinobacter	0.44	0.71	0.38	0.35	0.14	1.49	0.78
Neptunomonas	1.07	0.20	0.14	0.82	0.11	2.02	0.14
Formosa	0.08	0.49	1.09	1.61	0.58	0.12	0.18
Maribacter	0.06	0.51	0.64	2.14	0.72	0.06	0.06
Burkholderia	0.77	0.70	0.61	0.62	0.25	0.27	0.42
Ralstonia	0.05	0.05	0.05	0.06	3.62	0.02	0.09
Aeromonas	0.87	0.39	0.55	0.29	0.27	0.16	0.36
Alteromonas	0.92	0.43	0.42	0.17	0.36	0.14	0.32
Paenibacillus	1.47	0.12	0.36	0.24	0.44	0.10	0.13
Rhizobium	0.26	0.25	0.38	0.89	0.45	0.07	0.33
Algibacter	0.04	0.27	0.51	1.24	0.43	0.13	0.14
Olleya	0.03	0.24	0.47	1.35	0.44	0.07	0.14
Cellulophaga	0.06	0.42	0.76	1.01	0.30	0.11	0.09
Sphingomonas	0.12	0.21	0.21	0.58	0.23	0.16	0.65
Xanthomonas	0.55	0.48	0.39	0.36	0.19	0.25	0.29

Genus	SW0	SW4	SW8	SW04	SW08	SW0B4	SW0B8
Zhongshania	0.10	0.76	1.74	0.01	0.02	0.01	0.08
Sphingobium	0.13	0.16	0.20	0.46	0.20	0.15	0.70
Aequorivita	0.02	0.10	0.16	0.19	0.32	0.46	0.64
Celeribacter	0.17	0.52	0.48	0.70	0.33	0.02	0.09
Bradyrhizobium	0.23	0.22	0.30	0.68	0.32	0.05	0.25
Streptomyces	0.59	0.40	0.38	0.32	0.12	0.10	0.14
Mesorhizobium	0.19	0.17	0.28	0.62	0.32	0.04	0.23
Microbulbifer	0.31	0.85	0.32	0.09	0.08	0.06	0.18
Parvibaculum	0.01	0.01	0.02	1.19	0.72	0.00	0.02
Legionella	0.63	0.38	0.32	0.07	0.13	0.04	0.20
Phaeobacter	0.22	0.71	0.49	0.22	0.12	0.01	0.08
Haemophilus	0.64	0.36	0.19	0.18	0.18	0.12	0.11

Table S16. The mean proportions (%) and standard deviations (in parenthesis, *n = 2*) of the 50 predominant bacterial genera across all samples in seawater (SW), oil contaminated seawater (SWO), and biostimulated oil contaminated seawater (SWOB) according to taxonomic classification based on amplicon-sequencing and the SILVA database. The numbers in sample codes denote time in months.

Genus	SW0	SW4	SW8	SWO4	SWO8	SWOB4	SWOB8
Colwellia	6.45 (0.20)	4.63 (5.15)	3.53 (0.72)	2.75 (0.19)	9.91 (4.16)	1.07 (0.57)	1.80 (0.03)
Marinomonas	0.51 (0.01)	2.19 (2.23)	0.36 (0.11)	2.87 (0.37)	5.28 (0.97)	14.70 (6.71)	4.59 (2.25)
SM1A02	0.20 (0.18)	1.43 (0.84)	6.83 (7.40)	0.70 (0.71)	8.36 (10.87)	0.05 (0.01)	0.56 (0.02)
Paraperlucidibaca	0.07 (0.00)	0.15 (0.00)	0.39 (0.03)	0.08 (0.06)	0.24 (0.05)	2.97 (3.94)	16.56 (13.59)
Jejudonia	0.04 (0.01)	0.06 (0.02)	0.49 (0.21)	0.21 (0.22)	13.85 (19.01)	0.02 (0.00)	0.41 (0.02)
Ulvibacter	0.63 (0.05)	3.72 (1.19)	0.52 (0.43)	3.21 (2.66)	0.26 (0.19)	14.16 (18.75)	2.54 (3.13)
Pacificibacter	0.25 (0.09)	1.90 (1.58)	8.05 (10.77)	4.48 (1.16)	2.23 (2.84)	0.58 (0.43)	0.91 (0.38)
Aequorivita	0.09 (0.04)	0.07 (0.04)	0.24 (0.02)	0.16 (0.17)	0.17 (0.04)	3.20 (0.08)	11.55 (8.54)
Sphingorhabdus	0.17 (0.07)	0.67 (0.35)	0.83 (0.68)	0.32 (0.18)	0.18 (0.00)	5.95 (2.70)	5.93 (7.40)
Pseudomonas	0.26 (0.05)	0.20 (0.08)	0.25 (0.09)	0.78 (0.93)	0.24 (0.10)	10.76 (10.51)	3.70 (3.53)
Cycloclasticus	0.05 (0.01)	2.13 (0.21)	1.90 (0.91)	0.25 (0.15)	0.46 (0.36)	0.59 (0.74)	7.74 (4.46)
Sneathiella	0.13 (0.01)	0.33 (0.15)	0.35 (0.23)	0.69 (0.30)	0.52 (0.44)	3.35 (3.00)	3.95 (4.53)
Aurantivirga	0.24 (0.01)	2.55 (2.14)	6.88 (9.09)	0.28 (0.22)	0.13 (0.02)	0.18 (0.05)	0.21 (0.00)
Lacinutrix	0.27 (0.30)	0.10 (0.05)	0.15 (0.09)	1.08 (1.37)	3.13 (4.31)	0.03 (0.00)	0.07 (0.01)

Genus	SW0	SW4	SW8	SWO4	SWO8	SWOB4	SWOB8
Hyphomonas	0.11 (0.01)	0.16 (0.01)	0.06 (0.00)	3.20 (2.25)	1.56 (1.83)	0.10 (0.03)	0.11 (0.03)
Sulfitobacter	0.59 (0.02)	5.36 (4.76)	0.90 (1.14)	0.48 (0.47)	0.03 (0.01)	0.25 (0.01)	0.08 (0.00)
OM43_clade genus	0.64 (0.09)	0.74 (0.90)	3.84 (5.19)	0.03 (0.02)	0.07 (0.02)	0.04 (0.02)	0.12 (0.01)
Maribacter	0.07 (0.01)	0.61 (0.66)	0.22 (0.26)	1.71 (0.15)	0.98 (1.03)	0.08 (0.01)	0.11 (0.01)
Polaribacter	2.29 (0.15)	0.19 (0.03)	0.13 (0.07)	0.23 (0.03)	0.95 (1.19)	0.13 (0.03)	0.07 (0.04)
C1-B045	0.05 (0.01)	4.31 (1.83)	0.76 (0.38)	0.07 (0.05)	0.04 (0.03)	0.04 (0.01)	0.02 (0.00)
Dokdonia	0.12 (0.02)	2.51 (2.20)	1.78 (0.32)	0.11 (0.07)	0.03 (0.00)	0.08 (0.00)	0.05 (0.00)
Clade_Ia genus	4.10 (0.02)	0.14 (0.06)	0.06 (0.03)	0.11 (0.05)	0.06 (0.03)	0.10 (0.03)	0.02 (0.02)
Tenacibaculum	0.18 (0.00)	2.56 (2.93)	0.36 (0.47)	0.32 (0.31)	0.01 (0.01)	0.15 (0.01)	0.08 (0.04)
Paraglaciecola	0.06 (0.02)	0.18 (0.06)	0.21 (0.02)	0.18 (0.12)	0.64 (0.55)	0.89 (1.09)	0.36 (0.08)
SUP05_cluster	3.08 (0.10)	0.10 (0.06)	0.02 (0.01)	0.09 (0.02)	0.08 (0.02)	0.08 (0.02)	0.02 (0.02)
Loktanella	0.41 (0.02)	1.72 (2.00)	0.03 (0.01)	0.23 (0.23)	0.03 (0.01)	0.20 (0.03)	0.42 (0.42)
Parvibaculum	0.05 (0.01)	0.04 (0.03)	0.03 (0.01)	2.13 (2.88)	0.59 (0.81)	0.04 (0.01)	0.03 (0.00)
Oleispira	0.08 (0.02)	0.11 (0.07)	0.10 (0.08)	0.16 (0.18)	0.04 (0.02)	2.21 (2.90)	0.05 (0.01)
Pseudophaeobacter	0.01 (0.00)	1.72 (1.99)	0.82 (1.11)	0.03 (0.02)	0.01 (0.01)	0.02 (0.01)	0.02 (0.00)
Arcticiflavibacter	0.13 (0.03)	0.11 (0.00)	0.08 (0.03)	0.81 (0.34)	0.51 (0.40)	0.09 (0.05)	0.30 (0.29)
Winogradskyella	0.07 (0.01)	1.44 (1.57)	0.25 (0.32)	0.34 (0.29)	0.05 (0.05)	0.03 (0.02)	0.01 (0.00)
Cocleimonas	0.07 (0.02)	1.11 (1.38)	0.01 (0.01)	0.12 (0.10)	0.01 (0.00)	0.07 (0.00)	0.58 (0.52)
Zhongshania	<0.01 (0.00)	0.05 (0.06)	1.79 (1.92)	0.01 (0.01)	0.02 (0.00)	<0.01 (0.00)	0.04 (0.02)
Porticoccus	0.38 (0.01)	0.79 (0.23)	0.63 (0.83)	0.02 (0.00)	0.02 (0.00)	0.05 (0.03)	0.07 (0.01)
Flavobacterium	0.12 (0.01)	0.22 (0.08)	0.22 (0.06)	0.10 (0.04)	0.16 (0.03)	0.45 (0.29)	0.28 (0.00)
Hoeflea	0.01 (0.01)	0.12 (0.05)	0.07 (0.07)	0.21(0.10)	0.10 (0.00)	0.04 (0.01)	0.84 (0.51)
Amphritea	0.02 (0.01)	0.03 (0.03)	<0.01 (0.00)	0.24 (0.01)	0.01 (0.01)	0.94 (0.75)	0.11 (0.07)
Pseudohongiella	0.27 (0.01)	0.22 (0.25)	0.07 (0.01)	0.48 (0.63)	0.24 (0.03)	0.08 (0.08)	0.02 (0.02)
Neptunomonas	0.03 (0.00)	0.05 (0.02)	<0.01 (0.00)	0.24 (0.19)	<0.01 (0.00)	0.88 (1.01)	0.02 (0.01)
Cellulophaga	0.04 (0.01)	0.04 (0.02)	0.01 (0.01)	1.01 (0.84)	0.05 (0.04)	0.04 (0.00)	<0.01 (0.00)
Algimonas	0.01 (0.01)	0.10 (0.10)	0.81 (0.54)	0.13 (0.02)	0.02 (0.01)	0.01 (0.00)	0.02 (0.01)
Sedimentitalea	0.01 (0.01)	0.62 (0.30)	0.39 (0.52)	0.05 (0.02)	0.01 (0.00)	0.01 (0.00)	0.01 (0.00)
Nisaea	<0.01 (0.00)	<0.01 (0.00)	0.87 (1.18)	<0.01 (0.00)	0.02 (0.00)	<0.01 (0.00)	0.01 (0.01)
Lentilitoribacter	0.02 (0.01)	0.67 (0.13)	0.12 (0.17)	0.05 (0.04)	<0.01 (0.00)	0.02 (0.00)	0.01 (0.01)
Bryobacter	0.12 (0.06)	0.08 (0.06)	0.15 (0.02)	0.07 (0.04)	0.13 (0.02)	0.06 (0.00)	0.19 (0.00)
LS-NOB	0.69 (0.02)	0.05 (0.02)	0.01 (0.01)	0.04 (0.00)	0.01 (0.00)	0.03 (0.00)	0.01 (0.00)
Sphingomonas	0.06 (0.00)	0.09 (0.06)	0.13 (0.02)	0.06 (0.03)	0.09 (0.03)	0.08 (0.00)	0.15 (0.02)

Genus	SW0	SW4	SW8	SWO4	SWO8	SWOB4	SWOB8
Ca. Solibacter	0.06 (0.00)	0.07 (0.04)	0.16 (0.03)	0.04 (0.02)	0.09 (0.03)	0.06 (0.02)	0.15 (0.00)
Roseobacter	0.02 (0.00)	0.05 (0.00)	0.04 (0.02)	0.19 (0.09)	0.27 (0.22)	0.09 (0.11)	0.02 (0.02)
RB41	0.04 (0.00)	0.07 (0.05)	0.16 (0.02)	0.06 (0.05)	0.08 (0.01)	0.05 (0.00)	0.15 (0.03)

Table S17. The correlation (multivariate generalization of the Pearson correlation coefficient (RV)) between datasets of proportions of the 50 predominant bacterial genera (white background in table) and proportions of bacterial genera containing hydrocarbon degraders (yellow background in table) acquired with five different taxonomic classification methods (Kaiju with the NCBI-nr database, Kaiju with the MAR_{DB} database, Kraken2 with the Standard Kraken 2 database, Bracken with the Standard Kraken 2 database, and amplicon based sequencing with the SILVA database).

Classification method	Kaiju	Kaiju/MAR	Kraken2	Bracken	Amplicon
**Kaiju**		0.95	0.92	0.92	0.85
**Kaiju/MAR**	0.92		0.91	0.90	0.85
**Kraken2**	0.89	0.90		0.99	0.83
**Bracken**	0.88	0.89	0.99		0.81
**Amplicon**	0.58	0.61	0.74	0.76	

Table S18. The proportions (%) of the most abundant bacterial species (>1% of bacterial community in at least one treatment) in seawater (SW), oil contaminated seawater (SWO), and biostimulated oil contaminated seawater (SWOB) according to classification using Kaiju with the NCBI-nr database. The numbers in sample codes denote time in months.

Species	SW0	SW4	SW8	SWO4	SWO8	SWOB4	SWOB8
Clostridioides difficile	0.53	1.32	0.35	0.36	0.26	1.16	0.31
Ca Colwellia aromaticivorans	1.15	0.99	1.13	1.63	4.07	0.43	0.07
Cycloclasticus sp 44_32_T64	0.01	1.52	1.33	0.16	0.32	0.24	1.36
Cycloclasticus sp symbiont of Bathymodiolus heckerae	0.05	1.10	1.02	0.13	0.25	0.18	0.79
Cycloclasticus sp Phe_18	0.01	1.03	0.84	0.10	0.20	0.35	5.43
Hyphomonas sp BRH_c22	<0.01	0.02	0.02	3.25	0.23	<0.01	<0.01
Hyphomonas chukchiensis	<0.01	0.01	0.03	2.88	1.44	<0.01	0.02
Hyphomonas johnsonii	<0.01	0.01	0.01	2.15	0.15	<0.01	0.01
Hyphomonas oceanitis	<0.01	0.01	0.03	2.83	1.38	<0.01	0.02
Marinirhabdus gelatinilytica	<0.01	0.03	0.10	0.09	1.84	0.01	0.01

Species	SW0	SW4	SW8	SWO4	SWO8	SWOB4	SWOB8
Marinomonas primoryensis	0.01	0.81	0.05	3.08	6.22	6.79	2.27
Neptunomonas japonica	0.11	0.02	0.01	0.43	0.02	1.56	0.02
Nisaea denitrificans	0.02	0.01	2.74	0.01	0.01	0.01	0.04
Oleispira antarctica	0.17	0.09	0.18	0.02	0.10	4.35	0.03
Pacificibacter marinus	0.02	0.59	0.53	4.27	2.22	0.05	0.16
Paraglaciecola polaris	0.01	0.07	0.19	0.13	0.09	1.45	0.77
Paraperlucidibaca beakdonensis	0.02	0.01	0.01	<0.01	<0.01	3.38	14.57
Ca Pelagibacter sp IMCC9063	0.05	0.05	1.08	<0.01	<0.01	<0.01	<0.01
Ca Pelagibacter ubique	1.09	<0.01	0.07	<0.01	0.06	<0.01	<0.01
Pseudomonas fluorescens	0.04	0.02	0.02	0.02	0.02	1.42	0.12
Pseudomonas pelagia	0.01	0.01	0.31	0.61	1.49	0.72	0.70
Pseudomonas sabulinigri	0.01	0.01	0.01	0.12	0.07	0.75	5.61
Pseudomonas salina	0.02	0.02	0.32	5.17	0.03	5.49	3.65
Pseudomonas sp Y22	<0.01	<0.01	0.07	0.14	1.14	0.16	0.17
Sphingorhabdus sp M41	<0.01	0.21	0.09	0.09	0.01	0.72	1.70
Sphingorhabdus sp YGSMI21	<0.01	0.19	0.07	0.06	<0.01	0.53	1.17
Ca Thioglobus singularis	1.44	<0.01	0.02	0.01	0.12	<0.01	<0.01
Thioglobus sp NP1	1.33	<0.01	0.02	0.01	0.11	<0.01	<0.01
Ulvibacter litoralis	<0.01	0.09	0.06	1.23	0.24	3.05	0.92

Table S19. The number of detected genera, proportions (%) and estimated abundances (copies/mL) of bacterial genera containing petroleum hydrocarbon degraders (*n = 350*) in seawater (SW), oil contaminated seawater (SWO), and biostimulated oil contaminated seawater (SWOB) based on five taxonomic assignment methods (Kaiju with the NCBI-nr database, Kaiju with the MAR_{DB} database, Kraken2 with the Standard Kraken 2 database, Bracken with the Standard Kraken 2 database, and amplicon-based sequencing with the SILVA database). The numbers in sample codes denote time in months.

	SW0	SW4	SW8	SWO4	SWO8	SWOB4	SWOB8
				Genera			
Kaiju	338	338	338	338	338	338	338
Kaiju/MAR	226	226	226	226	226	226	226
Kraken2	279	279	279	279	279	279	279
Bracken	279	279	279	279	279	279	279

	SW0	SW4	SW8	SWO4	SWO8	SWOB4	SWOB8
Amplicon	105	111	126	120	127	117	134
				Proportions			
Kaiju	26.56	36.69	36.46	58.94	54.75	83.92	78.13
Kaiju/MAR	49.19	71.52	67.16	73.22	79.54	94.33	87.61
Kraken2	55.84	72.03	67.84	76.79	83.21	95.44	87.65
Bracken	54.81	73.33	66.49	74.36	80.93	94.22	87.17
Amplicon	13.60	29.39	14.49	21.98	24.15	65.11	62.77
				Estimated abundances			
Kaiju	2.71×10^5	1.37×10^6	1.91×10^6	1.71×10^6	2.51×10^6	6.95×10^7	9.69×10^7
Kaiju/MAR	5.02×10^5	2.66×10^6	3.51×10^6	2.13×10^6	3.65×10^6	7.81×10^7	1.09×10^8
Kraken2	5.70×10^5	2.68×10^6	3.55×10^6	2.23×10^6	3.82×10^6	7.90×10^7	1.09×10^8
Bracken	5.59×10^5	2.73×10^6	3.48×10^6	2.16×10^6	3.71×10^6	7.80×10^7	1.08×10^8
Amplicon	1.39×10^5	1.09×10^6	7.58×10^5	6.38×10^5	1.11×10^6	5.39×10^7	7.78×10^7

Table S20. Normalized hydrocarbon degradation related gene abundances presented as gene-specific reads per kilobase per genome equivalent (RPKG) in seawater (SW), oil-contaminated seawater (SWO), and biostimulated oil-contaminated seawater (SWOB). Numbers in sample codes denote time in months. Genes related to aliphatic, monoaromatic, polyaromatic, and various types of hydrocarbon compounds degradation are presented on pink, blue, yellow, and green background, respectively. Genes with abbreviations given in this study are supplemented with their KEGG code.

	Gene	RPKG						
		SW0	SW4	SW8	SWO4	SWO8	SWOB4	SWOB8
Aliphatic compounds	*alkB1_2*	0.36	0.79	0.62	0.53	0.52	0.85	0.87
	alkT/rubB	4.12	7.39	6.62	7.92	8.15	9.37	8.98
	almA	2.33	1.68	1.55	1.77	1.63	1.99	2.08
	bmoB	0.00	0.00	0.00	0.00	0.00	0.00	0.00
	bmoC	0.74	1.40	1.57	1.61	1.52	2.91	2.28
	bmoD	0.00	0.00	0.00	0.00	0.00	0.00	0.00
	bmoX	0.04	0.16	0.08	0.06	0.05	0.21	0.22
	bmoY	0.00	0.00	0.01	0.00	0.00	0.00	0.00
	bmoZ	0.00	0.00	0.00	0.00	0.00	0.00	0.00
	ladA	1.81	0.17	0.25	0.18	0.24	0.16	0.35
	MAH1	0.27	0.54	0.46	0.43	0.28	0.23	0.40

Gene		RPKG						
		SW0	SW4	SW8	SWO4	SWO8	SWOB4	SWOB8
	pmoA-amoA	0.05	0.24	0.12	0.13	0.08	0.05	0.19
	pmoB-amoB	0.04	0.18	0.05	0.05	0.07	0.06	0.08
	pmoC-amoC	0.06	0.30	0.18	0.14	0.24	0.10	0.26
	prmA	0.04	0.16	0.08	0.07	0.05	0.21	0.23
	prmB	2.44	4.83	4.34	4.91	5.31	7.75	5.82
	prmC	0.08	0.34	0.18	0.14	0.14	0.46	0.43
	prmD	0.00	0.25	0.09	0.11	0.12	0.54	0.41
	**Sum**	**12.37**	**18.45**	**16.22**	**18.04**	**18.39**	**24.89**	**22.60**
Monoaromatic compounds	*a-adh* (K00055)	8.59	7.57	6.72	8.02	8.75	7.85	7.91
	ADH1 (K18857)	3.54	3.36	2.96	3.77	3.96	3.68	3.95
	bsdC1	1.24	0.61	0.72	0.50	0.62	0.56	0.52
	bsdC2	0.77	0.44	0.55	0.40	0.48	0.44	0.40
	bsdD	0.00	0.27	0.19	0.14	0.10	0.28	0.36
	cymAa	0.29	0.89	0.71	0.49	0.46	0.81	0.94
	cymAb	1.21	2.69	2.46	2.72	2.70	4.52	3.35
	dmpK	0.04	0.32	0.16	0.22	0.25	0.66	0.42
	dmpL	0.06	0.26	0.17	0.15	0.15	0.30	0.36
	dmpM	0.11	0.97	0.49	0.42	0.31	1.12	1.37
	dmpN	0.05	0.22	0.10	0.11	0.06	0.22	0.30
	dmpO	0.06	0.37	0.21	0.26	0.23	0.85	0.45
	dmpP	2.44	4.62	4.15	4.70	4.91	7.68	5.40
	etbAa	2.68	2.95	2.20	1.89	1.88	2.88	3.25
	etbAb	0.52	3.43	2.31	1.70	2.05	3.62	4.16
	etbAc	2.51	3.34	2.48	1.73	1.99	2.08	2.25
	hcaB	35.21	31.47	29.13	32.78	36.36	33.12	36.03
	hcaC	8.62	8.11	6.97	6.48	6.65	6.57	6.57
	hcaD	5.19	8.77	7.59	9.17	9.12	10.01	9.60
	hcaE	2.93	3.26	2.48	2.03	2.06	3.05	3.48
	hcaF	0.34	2.99	2.05	1.42	1.37	3.21	3.76
	pchC	0.07	0.07	0.10	0.11	0.25	0.11	0.19
	pchF	1.63	1.74	1.38	1.69	2.03	1.37	1.50

	Gene	RPKG						
		SW0	SW4	SW8	SWO4	SWO8	SWOB4	SWOB8
	ped (K14746)	35.03	31.15	28.20	32.01	35.41	32.57	36.23
	phe (K03380)	0.89	1.15	0.84	0.99	1.23	1.33	1.35
	tmoA	0.05	0.22	0.10	0.10	0.06	0.23	0.30
	tmoB	0.08	0.38	0.11	0.13	0.07	0.13	0.49
	tmoC	7.43	9.96	7.27	7.25	7.43	7.84	7.85
	tmoD	0.10	0.84	0.39	0.42	0.27	0.97	1.19
	tmoE	0.09	0.42	0.22	0.17	0.15	0.57	0.53
	tmoF	2.72	5.06	4.44	5.17	5.41	8.06	5.89
	todA	1.36	3.19	2.58	3.42	3.17	4.40	4.06
	todB	1.54	1.22	0.75	0.59	0.91	1.30	1.50
	todC1	2.31	2.76	2.02	1.82	1.71	2.72	3.10
	todC2	0.28	2.49	1.44	1.08	1.12	2.29	2.95
	xylA	1.84	3.62	3.27	3.73	3.98	6.34	4.55
	xylC	8.04	9.87	7.45	10.27	11.31	11.86	9.96
	xylM	0.33	1.03	0.76	0.55	0.51	0.89	0.99
	**Sum**	**140.20**	**162.06**	**136.13**	**148.63**	**159.47**	**176.51**	**177.46**
Polyaromatic compounds	*CYP1A1*	1.03	0.85	0.79	1.05	0.75	0.55	1.01
	CYP1A2	0.71	0.81	0.72	0.78	0.53	0.45	0.82
	CYP2A6	0.19	0.43	0.37	0.36	0.27	0.20	0.30
	CYP3A4	0.32	0.62	0.54	0.48	0.32	0.25	0.57
	gst (K00799)	7.10	9.61	8.40	9.61	10.72	10.33	10.47
	GSTK1 (K13299)	0.71	0.74	0.82	0.83	1.16	1.42	1.28
	HPGSD (K04097)	1.16	2.05	1.49	1.91	2.24	2.58	2.45
	nahB	28.44	25.95	23.78	27.40	30.24	29.27	30.65
	nahC	0.21	0.89	0.58	0.65	0.56	0.96	1.06
	nidA	2.22	2.62	1.91	1.76	1.64	2.66	3.06
	nidB	0.34	3.06	1.84	1.41	1.41	3.36	3.85
	phdK	7.33	9.02	6.81	9.59	10.18	11.10	9.32
	**Sum**	**49.77**	**56.66**	**48.05**	**55.83**	**60.02**	**63.12**	**64.85**
V	*adh* (K00001)	10.12	9.44	8.27	10.33	11.11	10.75	10.22

Gene	RPKG						
	**SW0**	**SW4**	**SW8**	**SWO4**	**SWO8**	**SWOB4**	**SWOB8**
adhE (K04072)	2.81	3.15	2.65	4.24	4.35	5.78	4.45
adhP (K13953)	8.88	7.83	6.91	8.51	9.16	8.61	8.48
ALDH (K00128)	4.92	5.94	4.50	6.19	6.82	6.93	5.96
chnB	2.30	2.00	1.66	1.90	2.04	2.18	2.24
dbfA1	2.47	2.97	2.19	1.96	1.82	2.87	3.25
dbfA2	0.56	3.66	2.47	1.85	2.22	3.85	4.36
frmA	4.37	3.91	3.43	4.21	4.69	4.11	4.23
ligA	0.46	0.28	0.28	0.24	0.37	0.48	0.51
ligB	0.47	0.29	0.27	0.25	0.33	0.60	0.48
nagG	2.76	3.03	2.33	2.01	1.85	3.15	3.41
nagH	0.69	2.36	1.47	1.16	1.11	2.17	2.92
nahAa	2.60	4.99	4.42	5.04	5.35	8.23	5.93
nahAb	3.78	6.24	4.10	3.54	3.73	3.96	3.15
nahAc	2.28	2.75	2.08	1.84	1.70	2.70	3.06
nahAd	0.14	1.91	1.04	0.78	0.86	1.89	2.50
nmsA	0.00	0.00	0.00	0.01	0.00	0.04	0.00
nmsB	0.00	0.00	0.00	0.00	0.00	0.00	0.00
nmsC	0.00	0.00	0.00	0.00	0.00	0.00	0.00
pcaG	0.76	1.09	0.64	0.68	1.36	0.74	0.76
pcaH	0.36	0.60	0.37	0.30	0.59	0.32	0.37
phdE	18.38	16.31	14.81	17.43	19.41	18.44	20.79
phdI	0.33	0.06	0.23	0.02	0.06	0.10	0.12
phdJ	1.11	1.47	1.20	1.21	1.30	1.52	1.43
sdh (K00480)	2.90	4.13	2.96	4.07	4.20	4.36	3.97
yiaY	1.20	1.35	1.24	2.20	1.90	2.76	1.83
**Sum**	**74.67**	**85.75**	**69.53**	**79.97**	**86.33**	**96.56**	**94.40**

Table S21. The clustering of good quality (completeness >50%, contamination <10%) metagenome assembled genomes (MAGs) based on their average nucleotide identity (ANI) score along with their oil hydrocarbon degradation gene profiles. MAG affiliations are based on Kaiju with the NCBI-nr database.

Genes related to aliphatic, monoaromatic, polyaromatic, and various types of hydrocarbon compounds degradation are presented on pink, blue, yellow, and green background, respectively.

MASH clustering

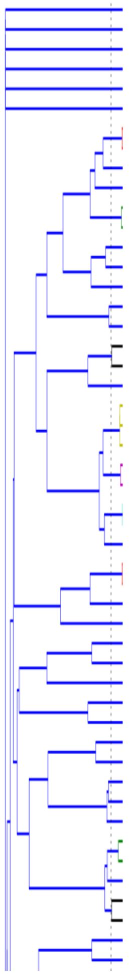

MAG code	MAG affiliation (Kaiju)	Completeness	Contamination	Genome size (bp)	*almA*	*ladA*	*MAH1*	*alkB1_2*	*bmoBCDXYZ*	*pmoABC*	*prmABCD*	*rubB*
N1_39	*Gemmatimonas*	73,61	4,50	3213185	+	-	+	-	-	-	-	+
N1_33	*Actinobacteria*	78,63	2,50	1716634	+	+	-	-	-	-	-	+
N1_40	*Gammaproteobacteria*	58,9	1,30	905332	-	-	-	+	-	-	-	+
N1_5	*SAR324 cluster bacterium*	65,84	1,78	1482109	+	+	-	-	-	-	-	+
N1_84	*Alphaproteobacteria*	75,6	2,72	2753296	+	+	-	-	-	-	-	+
N5_55	*Planctomycetes*	68,9	0,07	5060744	-	-	-	-	-	-	-	+
N8_28	*Loktanella salsilacus*	96,26	0,89	3998484	-	+	+	+	-	-	-	+
N7_19	*Loktanella salsilacus*	86,22	2,59	3745336	-	+	+	+	-	-	-	+
N3_17	*Sulfitobacter litoralis*	85,71	7,28	3240333	-	-	+	+	-	-	-	+
N5_42	*Sulfitobacter*	97,23	2,04	4532079	+	+	+	+	-	-	-	+
N5_77	*Rhodobacteraceae*	97,47	2,30	4501100	+	-	-	+	-	-	-	+
N2_20	*Rhodobacteraceae*	94,79	1,18	4570532	+	-	+	+	-	-	-	+
N5_62	*Rhodobacteraceae*	60,98	5,96	2641700	-	-	+	+	-	-	-	+
N2_33	*Rhodobacteraceae*	99,36	0,54	4179861	-	+	+	+	-	-	-	+
N5_81	*Alphaproteobacteria*	96,52	0,65	3714839	+	+	-	-	-	-	-	+
N6_37	*Hyphomonas oceanitis*	86,85	1,06	2966145	+	-	-	-	-	-	-	+
N3_10	*Hyphomonas*	86,14	7,53	4144464	+	+	+	+	-	-	-	+
N5_35	*Rhodobacteraceae*	96,53	3,32	4988138	+	-	+	+	-	-	-	+
N2_38	*Rhodobacteraceae*	91,36	2,20	3730622	+	-	+	+	-	-	-	+
N3_4	*Rhodobacteraceae*	88,87	0,69	3178656	-	-	+	+	-	-	-	+
N6_27	*Pacificibacter marinus*	87,34	3,98	3237602	-	+	+	+	-	-	-	+
N3_5	*Pacificibacter marinus*	63,26	1,72	2840962	-	-	+	+	-	-	-	+
N7_12	*Pacificibacter marinus*	97,36	1,56	4052540	-	+	+	+	-	-	-	+
N6_45	*Pacificibacter marinus*	96,17	2,16	3502443	+	-	+	+	-	-	-	+
N3_42	*Pacificibacter marinus*	71,18	0,00	2737578	+	-	+	+	-	-	-	+
N5_5	*Rhodobacteraceae*	98,94	0,30	3540597	-	+	+	+	-	-	-	+
N2_35	*Pacificibacter*	97,21	1,51	3464757	-	+	+	+	-	-	-	+
N2_25	*Pacificibacter*	62,74	2,59	2222289	-	-	-	+	-	-	-	+
N6_6	*Alphaproteobacteria*	97,83	0,00	2961614	+	+	-	+	-	-	-	+
N3_24	*Alphaproteobacteria*	94,35	0,00	2880468	+	+	-	+	-	-	-	+
N8_20	*Rhodobiaceae*	95	0,65	3261528	+	+	-	+	-	-	-	+
N1_78	*Euryarchaeota*	65,38	2,44	1598539	-	-	-	+	-	-	-	+
N5_20	*Nisaea denitrificans*	95,22	0,43	4410208	+	+	-	-	-	-	-	+
N1_67	*Rhodobacteraceae*	89,71	4,64	2953058	+	+	+	+	-	-	-	+
N7_6	*Alphaproteobacteria*	96,02	0,29	3899447	+	-	-	+	-	-	-	+
N5_27	*Alphaproteobacteria*	54,85	2,27	1386715	-	+	-	+	-	-	-	+
N3_28	*Albimonas donghaensis*	64,75	0,00	3554088	+	+	-	-	-	-	-	+
N8_13	*Hoeflea*	96,82	3,29	5231462	+	+	+	-	-	-	-	+
N3_16	*Hoeflea*	81,29	3,88	3404226	+	+	+	-	-	-	-	+
N3_43	*Maricaulis salignorans*	97,56	1,95	3093727	+	-	+	-	-	-	-	+
N2_46	*Maricaulis salignorans*	97,56	0,65	3116209	+	-	+	-	-	-	-	+
N5_39	*Maricaulis salignorans*	97,5	1,11	2973108	+	-	+	-	-	-	-	+
N4_13	*Sphingorhabdus sp M41*	74,41	0,00	2255826	+	-	+	-	-	-	-	+
N2_18	*Sphingomonadaceae*	98,86	0,90	3450886	+	-	+	+	-	-	+	+
N5_76	*Sphingorhabdus*	97,74	4,69	3670622	+	-	+	+	-	-	-	+
N8_27	*Sphingomonadales*	51,77	0,00	2177938	+	-	+	-	-	-	-	+
N7_28	*Sphingomonadales*	66,04	1,11	2876653	+	-	+	+	-	-	-	+
N8_8	*Alphaproteobacteria*	56,03	0,00	2546170	+	-	-	+	-	-	-	+
N7_14	*Pseudomonas salina*	99,13	0,43	4522719	+	-	-	-	-	-	-	+

MAG code	*a-adh* (K00055)	*ped* (K14746)	*PHE* (K03380)	*xylC*	*ADH1* (K18857)	*bsdCD*	*cymAab*	*dmpKLMNOP*	*etbAabc*	*hcaB*	*hcaCDEF*	*phcCF*	*tmoABCDEF*	*todABC1C2*	*xylAM*
N1_39	+	+	-	+	+	-	-	-	+	+	+	+	-	-	-
N1_33	+	+	+	+	+	-	-	-	-	+	+	-	-	-	-
N1_40	+	+	+	+	-	-	-	-	-	+	-	-	-	-	-
N1_5	+	+	-	+	+	-	-	-	-	+	+	-	-	-	-
N1_84	+	+	+	+	+	+	-	-	+	+	+	-	-	+	-
N5_55	+	+	+	+	+	+	-	-	+	+	+	-	-	+	-
N8_28	+	+	+	+	+	-	+	-	-	+	-	-	-	-	+
N7_19	+	+	+	+	+	-	+	-	-	+	-	-	-	-	+
N3_17	+	+	+	+	+	-	+	-	+	+	+	-	-	-	+
N5_42	+	+	+	+	+	-	+	-	+	+	+	-	-	-	+
N5_77	+	+	+	+	+	-	+	-	+	+	+	-	-	-	+
N2_20	+	+	+	+	+	-	+	-	+	+	+	-	-	-	+
N5_62	+	+	+	+	+	-	+	-	-	+	+	-	-	-	+
N2_33	+	+	+	+	+	-	+	-	-	+	-	-	-	-	+
N5_81	+	+	+	+	+	+	-	-	-	+	+	-	-	-	-
N6_37	+	+	+	+	+	-	-	-	+	+	+	-	-	+	-
N3_10	+	+	+	+	+	-	-	-	-	+	+	-	-	-	-
N5_35	+	+	+	+	+	-	+	-	+	+	+	-	-	-	+
N2_38	+	+	+	+	+	-	+	-	+	+	+	-	-	-	+
N3_4	+	+	+	+	+	-	+	-	-	+	+	-	-	-	+
N6_27	+	+	+	+	+	-	+	-	+	+	+	-	-	-	+
N3_5	+	+	+	+	+	-	+	-	+	+	+	-	-	+	+
N7_12	+	+	+	+	+	-	+	-	+	+	+	-	-	-	+
N6_45	+	+	+	+	+	-	-	-	+	+	+	-	-	-	-
N3_42	+	+	+	+	+	-	-	-	+	+	+	-	-	-	-
N5_5	+	+	+	+	+	-	+	-	+	+	+	-	-	+	+
N2_35	+	+	+	+	+	-	+	-	+	+	+	-	-	+	+
N2_25	+	+	+	+	+	-	+	-	+	+	+	-	-	+	+
N6_6	+	+	+	+	+	+	-	-	+	+	+	-	-	+	-
N3_24	+	+	+	+	+	+	-	-	-	+	+	-	-	-	-
N8_20	+	+	+	+	+	-	+	-	-	+	+	-	-	-	+
N1_78	+	+	-	+	+	+	-	-	-	+	-	-	-	-	-
N5_20	+	+	+	+	+	+	-	-	-	+	+	-	-	-	-
N1_67	+	+	+	+	+	-	-	-	-	+	+	-	-	-	+
N7_6	+	+	+	+	+	+	+	-	-	+	-	+	-	-	+
N5_27	+	+	+	+	+	+	-	-	+	+	+	-	-	-	-
N3_28	+	+	+	+	+	-	-	-	+	+	+	-	-	-	-
N8_13	+	+	+	+	+	+	-	-	+	+	+	-	-	-	-
N3_16	+	+	+	+	+	-	-	-	+	+	+	-	-	+	-
N3_43	+	+	+	+	+	+	-	-	-	+	+	-	-	-	-
N2_46	+	+	+	+	+	+	-	-	-	+	+	-	-	-	-
N5_39	+	+	+	+	+	+	-	-	-	+	+	-	-	-	-
N4_13	+	+	+	+	+	-	-	-	-	+	+	-	-	-	-
N2_18	+	+	+	+	+	-	+	+	+	+	+	-	+	-	+
N5_76	+	+	+	+	+	+	+	-	+	+	+	+	-	+	+
N8_27	+	+	+	+	+	-	-	-	+	+	+	-	-	-	-
N7_28	+	+	+	+	+	-	+	-	+	+	+	-	-	-	+
N8_8	+	+	+	+	+	+	+	-	-	+	-	-	-	-	+
N7_14	+	+	+	+	+	+	-	-	+	+	+	-	-	-	-

MAG code	*CYP1A1A2*	*CYP2A6*	*CYP3A4*	*gst* (K00799)	*GSTK1* (K13299)	*HPGDS*	*nahC*	*nidAB*	*nahB*	*phdK*
N1_39	+	+	+	-	-	-	-	-	+	+
N1_33	+	+	-	-	-	-	-	-	+	+
N1_40	-	-	-	+	-	-	-	-	+	+
N1_5	-	-	-	+	+	+	-	-	+	+
N1_84	-	-	-	+	+	+	-	+	+	+
N5_55	-	-	-	-	-	-	-	+	+	+
N8_28	+	+	+	+	+	+	-	-	+	+
N7_19	+	+	+	+	+	+	-	-	+	+
N3_17	+	+	+	+	+	+	-	-	+	+
N5_42	+	+	+	+	+	+	+	-	+	+
N5_77	+	-	+	+	+	+	+	-	+	+
N2_20	+	+	+	+	+	+	+	-	+	+
N5_62	+	+	+	+	+	+	-	-	+	+
N2_33	+	+	+	+	+	+	+	-	+	+
N5_81	-	-	-	+	+	+	+	-	+	+
N6_37	+	-	-	+	+	+	+	+	+	+
N3_10	+	-	+	+	+	+	-	-	+	+
N5_35	+	+	+	+	+	+	+	-	+	+
N2_38	+	+	+	+	+	+	+	-	+	+
N3_4	+	+	+	+	+	+	-	-	+	+
N6_27	+	+	+	+	-	+	-	-	+	+
N3_5	+	+	+	+	+	+	-	-	+	+
N7_12	+	+	+	+	+	+	+	-	+	+
N6_45	+	+	+	+	+	+	-	-	+	+
N3_42	+	+	+	+	+	+	-	-	+	+
N5_5	+	+	+	+	-	+	+	-	+	+
N2_35	+	+	+	+	-	+	+	-	+	+
N2_25	+	-	-	+	-	+	-	+	+	+
N6_6	+	-	+	+	+	+	+	+	+	+
N3_24	+	-	+	+	+	+	-	-	+	+
N8_20	+	-	+	+	+	+	-	-	+	+
N1_78	-	-	-	+	-	+	-	-	+	+
N5_20	-	-	-	+	+	+	-	-	+	+
N1_67	+	+	+	+	+	+	+	-	+	+
N7_6	-	-	-	+	+	+	-	-	+	+
N5_27	-	-	-	+	-	+	-	-	+	+
N3_28	-	-	-	+	+	+	-	-	+	+
N8_13	+	+	+	+	+	+	-	-	+	+
N3_16	+	+	+	+	+	+	-	-	+	+
N3_43	+	+	+	+	+	+	-	-	+	+
N2_46	+	+	+	+	+	+	-	-	+	+
N5_39	+	+	+	+	-	+	-	-	+	+
N4_13	+	+	+	+	+	+	+	-	+	+
N2_18	+	+	+	+	+	+	+	-	+	+
N5_76	+	+	+	+	+	+	+	+	+	+
N8_27	+	+	+	+	+	+	+	+	+	+
N7_28	+	+	+	+	+	+	-	-	+	+
N8_8	+	-	-	+	+	+	-	-	+	+
N7_14	+	-	-	+	+	+	-	-	+	+

MAG code	*adh* (K00001)	*adhE* (K04072)	*adhP* (K13953)	*ALDH* (K00128)	*chnB*	*frmA*	*phdE*	*sdh* (K00480)	*dbfA1A2*	*ligAB*	*nagGH*	*nahAabcd*	*pcaGH*	*phdIJ*	*yaiY*
N1_39	+	+	+	+	+	+	+	+	-	-	-	-	-	-	-
N1_33	+	+	+	+	+	+	+	+	-	-	+	-	-	-	-
N1_40	+	+	+	+	+	+	+	+	-	-	-	-	-	-	-
N1_5	+	+	+	+	+	+	+	+	-	-	-	-	-	-	+
N1_84	+	+	+	+	+	+	+	+	+	-	+	+	-	-	+
N5_55	+	+	+	+	+	+	+	+	+	-	-	+	+	-	+
N8_28	+	+	+	+	+	+	+	+	-	-	-	-	+	-	-
N7_19	+	+	+	+	+	+	+	+	-	-	-	-	+	-	-
N3_17	+	+	+	+	+	+	+	+	-	-	-	+	+	-	+
N5_42	+	+	+	+	+	+	+	+	-	-	-	+	+	-	+
N5_77	+	+	+	+	+	+	+	+	-	-	-	+	+	-	+
N2_20	+	+	+	+	+	+	+	+	-	-	-	+	+	-	+
N5_62	+	+	+	+	+	+	+	+	-	-	-	-	+	-	-
N2_33	+	+	+	+	+	+	+	+	-	-	-	-	+	-	+
N5_81	+	+	+	+	+	+	+	+	-	-	-	-	+	+	+
N6_37	+	+	+	+	+	+	+	+	+	-	+	+	-	-	+
N3_10	+	+	+	+	+	+	+	+	-	-	+	-	-	-	+
N5_35	+	+	+	+	+	+	+	+	-	-	-	+	+	-	+
N2_38	+	+	+	+	+	+	+	+	-	-	-	+	+	-	+
N3_4	+	+	+	+	+	+	+	+	-	+	-	-	-	-	+
N6_27	+	+	+	+	+	+	+	+	-	-	-	+	+	-	+
N3_5	+	+	+	+	+	+	+	+	-	-	-	+	+	-	+
N7_12	+	+	+	+	+	+	+	+	-	-	-	+	+	-	+
N6_45	+	+	+	+	+	+	+	+	-	-	-	-	+	-	+
N3_42	+	+	+	+	+	+	+	+	-	-	-	-	-	-	+
N5_5	+	+	+	+	+	+	+	+	-	-	-	+	+	+	+
N2_35	+	+	+	+	+	+	+	+	-	-	-	+	+	+	+
N2_25	+	+	+	+	-	+	+	+	+	-	+	+	-	-	-
N6_6	+	+	+	+	+	+	+	+	+	-	+	-	-	-	+
N3_24	+	+	+	+	+	+	+	+	-	-	-	-	-	-	+
N8_20	+	+	+	+	+	+	+	+	-	-	-	-	-	-	+
N1_78	+	+	+	+	+	+	+	+	-	-	-	-	-	-	-
N5_20	+	+	+	+	+	+	+	+	-	-	-	-	-	+	+
N1_67	+	+	+	+	+	+	+	+	-	-	-	-	+	-	+
N7_6	+	+	+	+	+	+	+	+	-	-	-	-	+	-	+
N5_27	+	+	+	+	+	+	+	+	-	-	-	-	-	+	-
N3_28	+	+	+	+	+	+	+	+	-	-	-	+	+	-	+
N8_13	+	+	+	+	+	+	+	+	-	-	-	+	+	-	+
N3_16	+	+	+	+	+	+	+	+	+	-	-	+	+	-	+
N3_43	+	+	+	+	+	+	+	+	-	-	-	-	-	-	+
N2_46	+	+	+	+	+	+	+	+	-	-	-	-	-	-	+
N5_39	+	+	+	+	+	+	+	+	-	-	-	-	-	-	+
N4_13	+	+	+	+	+	+	+	+	-	-	-	-	-	-	+
N2_18	+	+	+	+	+	+	+	+	-	-	-	-	-	-	+
N5_76	+	+	+	+	+	+	+	+	+	+	+	+	-	-	+
N8_27	+	+	+	+	+	+	+	+	+	-	+	-	-	-	+
N7_28	+	+	+	+	+	+	+	+	-	-	-	+	-	-	+
N8_8	+	+	+	+	+	+	+	+	-	-	-	-	+	-	+
N7_14	+	+	+	+	+	+	+	+	-	-	-	+	+	-	+

MASH clustering

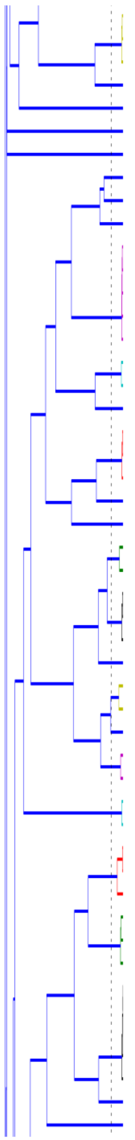

MAG code	MAG affiliation (Kaiju)	Completeness	Contamination	Genome size (bp)	almA	ladA	MAH1	alkB1_2	bmoBCDXYZ	pmoABC	prmABCD	rubB
N6_46	*Alphaproteobacteria*	97,83	0,65	3644458	+	-	-	-	-	-	-	+
N4_24	*Alphaproteobacteria*	94,25	1,96	3403453	+	-	-	-	-	-	-	+
N3_35	*Alphaproteobacteria*	97,39	0,65	3318039	+	-	-	-	-	-	-	+
N4_19	*Alphaproteobacteria*	96,77	0,54	3779820	+	+	-	+	-	-	-	+
N2_8	*OM182 clade*	66,43	7,93	2050206	+	-	-	+	-	-	-	+
N3_11	*Hyphomonadaceae*	62,23	1,84	1870271	+	-	+	+	-	-	-	+
N5_60	*Bacteria*	92,47	3,53	9819246	-	-	+	+	-	-	-	+
N8_25	*Cocleimonas flava*	82,53	7,65	4680764	-	-	+	+	-	-	+	+
N6_16	*Paraglaciecola*	95,17	4,55	5140532	+	+	-	-	-	-	-	+
N4_1	*Oceanospirillaceae*	98,41	5,89	4581457	+	-	-	+	-	-	+	+
N8_15	*Marinomonas primoryensis*	98,28	8,62	3668965	-	-	-	-	-	-	-	+
N4_7	*Marinomonas primoryensis*	94,17	2,15	3807449	-	-	-	-	-	-	-	+
N6_2	*Marinomonas primoryensis*	96,1	0,79	3892185	-	-	-	-	-	-	-	+
N2_4	*Marinomonas primoryensis*	98,14	1,79	3481648	-	-	-	-	-	-	-	+
N3_37	*Marinomonas primoryensis*	100	8,62	3814937	-	-	-	-	-	-	-	+
N8_4	*Cycloclasticus*	98,33	3,91	2778661	+	+	-	+	-	-	+	+
N4_17	*Gammaproteobacteria*	72,41	1,72	2713486	+	+	-	+	-	-	+	+
N6_34	*Gammaproteobacteria*	90,03	2,78	2959112	-	-	-	-	-	-	-	+
N7_20	*Alteromonadaceae*	98,09	0,34	5291344	-	-	-	-	-	-	+	+
N4_21	*Paraglaciecola polaris*	95,29	1,35	5009859	-	-	-	-	-	-	-	+
N8_18	*Paraglaciecola polaris*	92,14	0,82	4900148	+	-	-	-	-	-	-	+
N5_51	*Gammaproteobacteria*	67,38	1,72	2707270	-	-	+	-	-	-	-	+
N3_34	*Alphaproteobacteria*	65,67	3,45	3224391	-	-	-	-	-	-	-	+
N8_5	*Alteromonadales*	67,79	7,60	2456130	-	-	-	-	-	-	-	+
N7_10	*Alteromonadales*	85,35	3,64	3932559	-	-	-	-	-	+	+	+
N6_17	*Colwellia*	91,22	2,64	2837974	-	-	-	-	+	-	+	+
N3_15	*Colwellia*	90,62	1,17	2795101	-	-	-	-	+	-	+	+
N1_29	*Ca. Colwellia aromaticivorans*	83,4	2,15	2324013	-	-	-	-	-	-	-	+
N5_15	*Gammaproteobacteria*	63,79	1,72	2752241	+	-	-	-	-	-	-	+
N7_23	*Gammaproteobacteria*	52,6	2,12	1459825	-	-	-	-	-	-	-	+
N6_18	*Gammaproteobacteria*	92,22	7,00	2739396	-	-	-	+	-	-	-	+
N5_22	*Gammaproteobacteria*	61,83	5,17	3431796	-	-	-	+	-	-	-	+
N6_25	*Gammaproteobacteria*	87,33	4,41	2237402	-	-	-	+	-	-	-	+
N5_80	*Gammaproteobacteria*	90,59	3,78	2194902	-	-	-	+	-	-	-	+
N4_6	*Neptunomonas japonica*	99,14	2,86	4687687	-	-	-	-	-	-	-	+
N3_41	*Neptunomonas japonica*	97,29	3,79	4329920	-	-	+	-	-	-	-	+
N8_6	*Pseudomonas*	99,57	2,24	4104107	+	+	-	+	-	-	-	+
N7_27	*Pseudomonas*	93,53	2,67	3580099	+	+	-	+	-	-	-	+
N4_20	*Pseudomonas*	62,07	0,00	1399208	+	-	-	-	-	-	-	+
N4_2	*Amphritea sp, MCCC 1K03512*	97,96	1,43	3929493	+	-	-	-	-	-	-	+
N3_18	*Amphritea sp, MCCC 1K03512*	97,42	1,50	4043495	+	-	-	-	-	-	-	+
N8_22	*Amphritea*	62,07	0,00	3111222	+	-	-	-	-	-	-	+
N4_9	*Pseudomonas*	99,57	1,00	4245038	+	-	-	+	-	-	-	+
N3_23	*Pseudomonas*	99,57	1,00	4246181	+	-	-	+	-	-	-	+
N8_3	*Pseudomonas salina*	99,57	1,00	4160442	+	-	-	+	-	-	-	+
N7_9	*Pseudomonas salina*	78,02	0,92	3382028	+	-	-	+	-	-	-	+
N5_23	*Pseudomonas salina*	86,79	2,24	3144251	+	-	-	+	-	-	-	+
N6_7	*Pseudomonas*	99,14	0,73	4238583	+	+	-	+	-	-	-	+
N4_12	*Pseudomonas*	97,69	3,64	6291778	+	-	-	+	-	-	-	+

MAG code	a-adh (K00055)	ped (K14746)	PHE (K03380)	xylC	ADH1 (K18857)	bsdCD	cymAab	dmpKLMNOP	etbAabc	hcaB	hcaCDEF	phcCF	tmoABCDEF	todABC1C2	xylAM
N6_46	+	+	+	+	+	+	-	-	+	+	+	-	-	-	-
N4_24	+	+	+	+	+	+	-	-	+	+	+	-	-	+	-
N3_35	+	+	+	+	+	+	-	-	+	+	+	-	-	-	-
N4_19	+	+	+	+	+	+	+	-	-	+	-	-	-	-	+
N2_8	+	+	+	+	+	+	-	-	-	+	-	-	-	-	-
N3_11	+	+	+	+	+	-	-	-	+	+	+	-	-	+	-
N5_60	+	+	+	+	+	-	+	-	+	+	+	-	-	-	+
N8_25	+	+	+	+	+	-	-	+	+	+	+	-	+	-	-
N6_16	+	+	+	+	+	-	-	-	+	+	+	-	-	+	-
N4_1	+	+	+	+	+	+	+	+	+	+	+	-	+	+	+
N8_15	+	+	+	+	+	-	-	-	+	+	+	-	-	-	-
N4_7	+	+	+	+	+	-	-	-	+	+	+	-	-	+	-
N6_2	+	+	+	+	+	-	-	-	+	+	+	-	-	+	-
N2_4	+	+	+	+	+	-	-	-	-	+	+	-	-	-	-
N3_37	+	+	+	+	+	-	-	-	+	+	+	-	-	+	-
N8_4	+	+	+	+	+	+	+	+	+	+	+	+	+	+	+
N4_17	+	+	+	+	+	+	+	+	+	+	+	+	+	+	+
N6_34	+	+	+	+	+	+	-	-	-	+	-	-	-	-	-
N7_20	+	+	+	+	+	-	-	+	-	+	+	-	+	-	-
N4_21	+	+	+	+	+	-	-	-	-	+	+	-	-	-	-
N8_18	+	+	+	+	+	-	-	-	-	+	+	-	-	-	-
N5_51	+	+	+	+	+	+	-	-	-	+	+	-	-	-	-
N3_34	+	+	+	+	+	+	-	-	+	+	+	-	-	+	-
N8_5	+	+	+	+	+	+	-	-	-	+	-	-	-	-	-
N7_10	+	+	+	+	+	+	-	+	+	+	+	-	+	+	-
N6_17	+	+	+	+	+	+	-	+	-	+	-	-	+	-	-
N3_15	+	+	+	+	-	+	-	+	-	+	-	-	+	-	-
N1_29	-	+	+	+	-	+	-	-	-	+	-	-	-	-	-
N5_15	+	+	+	+	+	+	-	-	-	+	-	+	-	-	-
N7_23	+	+	+	+	-	-	-	-	-	+	-	-	-	-	-
N6_18	+	+	+	+	+	+	-	-	+	+	+	-	-	-	-
N5_22	+	+	+	+	+	+	+	-	+	+	+	-	-	+	+
N6_25	-	+	+	+	-	+	+	-	-	+	-	+	-	-	+
N5_80	+	+	+	+	-	+	+	-	-	+	-	-	-	-	+
N4_6	+	+	+	+	+	+	-	-	-	+	+	-	-	-	-
N3_41	+	+	+	+	+	+	-	-	-	+	+	-	-	-	-
N8_6	+	+	+	+	+	+	+	-	+	+	+	-	-	+	+
N7_27	+	+	+	+	+	+	+	-	-	+	+	-	-	-	+
N4_20	+	+	-	+	+	-	-	-	-	+	-	-	-	-	-
N4_2	+	+	+	+	+	+	-	-	-	+	+	-	-	-	-
N3_18	+	+	+	+	+	+	-	-	-	+	+	-	-	-	-
N8_22	+	+	+	+	+	+	-	-	-	+	+	-	-	-	-
N4_9	+	+	+	+	+	+	+	-	-	+	-	-	-	-	+
N3_23	+	+	+	+	+	+	+	-	+	+	+	-	-	-	+
N8_3	+	+	+	+	+	+	+	-	-	+	-	-	-	-	+
N7_9	+	+	+	+	+	+	+	-	-	+	-	-	-	-	+
N5_23	+	+	+	+	+	+	+	-	-	+	-	-	-	-	+
N6_7	+	+	+	+	+	+	+	-	+	+	+	-	-	+	+
N4_12	+	+	+	+	+	+	+	-	+	+	+	-	-	+	+

MAG code	CYP1A1A2	CYP2A6	CYP3A4	gst (K00799)	GSTK1 (K13299)	HPGDS	nahC	nidAB	nahB	phdK
N6_46	-	-	-	+	+	+	-	-	+	+
N4_24	-	-	-	+	+	+	+	-	+	+
N3_35	-	-	-	+	+	+	-	-	+	+
N4_19	+	-	-	+	+	+	-	-	+	+
N2_8	-	-	-	+	+	+	-	-	+	+
N3_11	+	+	+	+	+	+	-	+	+	+
N5_60	+	+	+	+	-	-	-	-	+	+
N8_25	+	+	+	+	-	+	+	-	+	+
N6_16	+	-	-	+	-	+	-	-	+	+
N4_1	+	-	-	+	-	+	+	+	+	+
N8_15	-	-	-	+	-	+	-	+	+	+
N4_7	-	-	-	+	-	+	-	+	+	+
N6_2	-	-	-	+	-	+	+	+	+	+
N2_4	-	-	-	+	-	+	-	-	+	+
N3_37	+	-	-	+	-	+	-	+	+	+
N8_4	-	-	-	+	+	+	+	+	+	+
N4_17	-	-	-	+	+	+	+	+	+	+
N6_34	-	-	-	+	-	+	-	-	+	+
N7_20	-	-	-	+	+	+	+	-	+	+
N4_21	-	-	-	+	+	+	-	-	+	+
N8_18	-	-	-	+	+	+	-	-	+	+
N5_51	+	+	+	+	-	+	-	-	+	+
N3_34	-	-	-	+	+	+	+	+	+	+
N8_5	-	-	-	+	-	+	-	-	+	+
N7_10	-	-	-	+	-	+	+	+	+	+
N6_17	-	-	-	+	+	+	+	-	+	+
N3_15	-	-	-	+	+	+	+	-	+	+
N1_29	-	-	-	+	+	+	-	-	+	+
N5_15	-	-	-	+	-	+	-	-	+	+
N7_23	-	-	-	+	-	+	-	-	+	+
N6_18	-	-	-	+	-	-	-	-	+	+
N5_22	-	-	-	+	-	-	-	+	+	+
N6_25	-	-	-	+	-	+	-	-	+	+
N5_80	-	-	-	+	-	-	-	-	+	+
N4_6	-	-	-	+	+	+	-	-	+	+
N3_41	+	+	+	+	+	+	-	-	+	+
N8_6	+	-	-	+	+	+	+	+	+	+
N7_27	-	-	-	+	+	+	-	-	+	+
N4_20	-	-	-	+	+	+	-	-	+	+
N4_2	-	-	-	+	+	+	-	-	+	+
N3_18	-	-	-	+	+	+	-	-	+	+
N8_22	-	-	-	+	+	+	-	-	+	+
N4_9	-	-	-	+	+	+	-	-	+	+
N3_23	-	-	-	+	+	+	+	+	+	+
N8_3	-	-	-	+	+	+	-	-	+	+
N7_9	-	-	-	+	+	+	-	-	+	+
N5_23	-	-	-	+	+	+	-	-	+	+
N6_7	-	-	-	+	+	+	+	+	+	+
N4_12	-	-	-	+	-	+	-	+	+	+

MAG code	adh (K00001)	adhE (K04072)	adhP (K13953)	ALDH (K00128)	chnB	frmA	phdE	sdh (K00480)	dbfA1A2	ligAB	nagGH	nahAabcd	pcaGH	phdIJ	yaiY
N6_46	+	+	+	+	+	+	+	+	-	-	-	+	+	-	+
N4_24	+	+	+	+	+	+	+	+	-	+	-	+	+	-	+
N3_35	+	+	+	+	+	+	+	+	-	-	-	+	+	-	+
N4_19	+	+	+	+	+	+	+	+	-	-	-	-	-	-	+
N2_8	+	+	+	+	+	+	+	+	-	-	-	-	-	-	-
N3_11	+	+	+	+	+	+	+	+	+	-	+	-	-	-	+
N5_60	+	+	+	+	-	+	+	+	-	-	-	+	+	-	-
N8_25	+	+	+	+	+	+	+	+	-	-	-	+	-	-	+
N6_16	+	+	+	+	+	+	+	+	+	-	+	+	-	+	+
N4_1	+	+	+	+	+	+	+	+	+	-	+	+	-	-	+
N8_15	+	+	+	+	+	+	+	+	+	+	-	+	+	-	+
N4_7	+	+	+	+	+	+	+	+	+	+	+	+	+	+	+
N6_2	+	+	+	+	+	+	+	+	+	+	+	+	+	-	+
N2_4	+	+	+	+	+	+	+	+	-	+	-	+	-	-	-
N3_37	+	+	+	+	+	+	+	+	+	+	+	+	+	+	-
N8_4	+	+	+	+	+	+	+	+	+	-	+	+	-	-	-
N4_17	+	+	+	+	+	+	+	+	+	-	+	+	-	-	-
N6_34	+	+	+	+	+	+	+	+	-	-	-	-	-	-	+
N7_20	+	+	+	+	+	+	+	+	-	+	-	+	-	-	+
N4_21	+	+	+	+	+	+	+	+	-	+	-	+	-	-	+
N8_18	+	+	+	+	+	+	+	+	-	+	-	+	-	-	+
N5_51	+	+	+	+	-	+	+	+	-	-	-	-	-	-	+
N3_34	+	+	+	+	-	+	+	+	+	-	-	+	-	-	+
N8_5	+	+	+	+	+	+	+	+	-	-	-	-	-	-	+
N7_10	+	+	+	+	+	+	+	+	+	-	-	+	-	-	+
N6_17	+	+	+	+	-	+	+	+	-	-	-	-	-	-	+
N3_15	+	+	+	+	+	+	+	+	-	-	-	-	-	-	+
N1_29	+	+	+	+	+	+	+	+	-	-	-	-	-	-	+
N5_15	+	+	+	+	+	+	+	+	-	-	-	-	-	-	+
N7_23	+	+	+	+	-	-	+	+	-	-	-	-	-	-	+
N6_18	+	+	+	+	+	+	+	+	+	-	-	+	-	-	+
N5_22	+	+	+	+	-	+	+	+	+	-	+	+	-	-	+
N6_25	+	+	-	+	+	-	+	+	-	-	-	-	-	-	+
N5_80	+	+	+	+	+	+	+	+	-	-	-	-	-	-	+
N4_6	+	+	+	+	+	+	+	+	-	-	-	+	+	-	+
N3_41	+	+	+	+	+	+	+	+	-	-	-	+	-	-	+
N8_6	+	+	+	+	+	+	+	+	+	-	-	+	+	-	+
N7_27	+	+	+	+	+	+	+	+	-	-	-	-	+	-	+
N4_20	+	+	+	+	+	+	+	+	-	-	-	-	-	-	-
N4_2	+	+	+	+	+	+	+	+	-	-	-	+	-	-	+
N3_18	+	+	+	+	+	+	+	+	-	-	-	+	-	-	+
N8_22	+	+	+	+	+	+	+	+	-	-	-	+	-	-	+
N4_9	+	+	+	+	+	+	+	+	-	-	-	-	-	-	+
N3_23	+	+	+	+	+	+	+	+	+	-	+	-	-	-	+
N8_3	+	+	+	+	+	+	+	+	-	-	-	-	-	-	+
N7_9	+	+	+	+	+	+	+	+	-	-	-	-	-	-	+
N5_23	+	+	+	+	+	+	+	+	-	-	-	-	-	-	+
N6_7	+	+	+	+	+	+	+	+	+	-	-	+	-	-	+
N4_12	+	+	+	+	+	+	+	+	+	+	-	+	+	-	+

MASH clustering

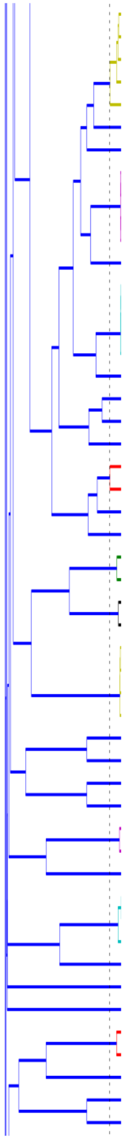

MAG code	MAG affiliation (Kaiju)	Completeness	Contamination	Genome size (bp)	almA	ladA	MAH1	alkB1_2	bmoBCDXYZ	pmoABC	prmABCD	rubB	a-adh (K00055)	ped (K14746)	PHE (K03380)	xylC	ADH1 (K18857)	bsdCD	cymAab	dmpKLMNOP	etbAabc	hcaB	hcaCDEF	phcCF	tmoABCDEF	todABC1C2	xylAM	CYP1A1A2	CYP2A6	CYP3A4	gst (K00799)	GSTK1 (K13299)	HPGDS	nahC	nidAB	nahB	phdK	adh (K00001)	adhE (K04072)	adhP (K13953)	ALDH (K00128)	chnB	frmA	phdE	sdh (K00480)	dbfA1A2	ligAB	nagGH	nahAabcd	pcaGH	phdIJ	yaiY
N4_23	*Gammaproteobacteria*	95,66	1,40	2496415	+	-	-	+	-	+	-	+	+	+	+	+	+	+	+	-	+	+	+	+	-	+	+	+	-	-	+	+	+	+	+	+	+	+	+	+	+	+	+	+	+	+	-	+	+	-	-	-
N3_12	*Cycloclasticus*	77,46	8,11	2085704	+	-	-	+	-	+	-	+	+	+	-	+	+	+	+	-	+	+	+	+	-	+	+	+	-	+	+	+	+	+	+	+	+	+	+	+	+	+	+	+	+	+	-	+	+	-	-	-
N6_15	*Cycloclasticus*	67,02	2,51	1493680	+	-	-	+	-	+	-	+	+	+	+	+	+	+	+	-	+	+	+	-	-	+	+	+	-	+	+	-	+	+	+	+	+	+	+	+	+	+	+	+	+	+	-	+	+	-	-	-
N2_43	*Piscirickettsiaceae*	62,93	5,17	2087224	+	-	-	+	-	-	+	+	+	+	+	+	+	+	+	+	+	+	+	-	+	+	+	+	-	-	+	+	+	+	+	+	+	+	+	+	+	+	+	+	+	+	-	+	+	-	-	-
N8_17	*Cycloclasticus*	77,24	5,00	2173765	+	+	-	+	-	-	+	+	+	+	+	+	+	+	+	+	+	+	+	+	+	+	+	+	-	-	+	-	+	+	+	+	+	+	+	+	+	+	+	+	+	+	-	+	+	-	-	-
N7_26	*Gammaproteobacteria*	98,2	1,85	2717576	+	-	-	-	-	-	-	+	+	+	+	+	+	+	-	-	-	+	+	-	-	-	-	-	-	-	+	-	+	-	-	+	+	+	+	+	+	+	+	+	+	-	-	-	+	-	-	+
N6_35	*SAR86 cluster bacterium*	93,13	2,96	3565847	-	-	-	-	-	-	-	+	+	+	+	+	+	+	-	-	-	+	-	-	-	-	-	-	-	-	+	+	+	-	-	+	+	+	+	+	+	+	+	+	+	-	-	-	-	-	-	-
N6_11	*Gammaproteobacteria*	93,74	0,37	2296743	+	+	+	+	-	+	-	+	+	+	+	+	+	+	+	-	+	+	+	-	-	+	+	+	+	+	+	+	+	+	+	+	+	+	+	+	+	+	+	+	+	+	-	+	+	-	-	-
N3_27	*Gammaproteobacteria*	93,41	1,19	2291261	+	+	+	+	-	+	-	+	+	+	+	+	+	+	+	-	+	+	+	-	-	+	+	+	+	+	+	+	+	+	+	+	+	+	+	+	+	+	+	+	+	+	-	+	+	-	-	-
N5_14	*Gammaproteobacteria*	77,59	0,00	2153643	+	+	+	+	-	+	-	+	+	+	+	+	+	+	+	-	+	+	+	-	-	+	+	+	+	+	+	+	+	+	+	+	+	+	+	+	+	+	+	+	+	+	-	+	+	-	-	-
N2_26	*Gammaproteobacteria*	93,14	1,48	2241751	+	+	+	+	-	+	-	+	+	+	+	+	+	+	+	-	+	+	+	-	-	+	+	+	+	+	+	+	+	+	+	+	+	+	+	+	+	+	+	+	+	+	-	+	+	-	-	-
N4_18	*Marinobacter antarcticus*	99,14	0,59	4058849	+	+	-	+	-	-	-	+	+	+	+	+	+	+	+	-	-	+	-	-	-	-	+	+	-	-	+	+	+	-	-	+	+	+	+	+	+	+	+	+	+	-	-	-	-	-	-	+
N6_10	*Gammaproteobacteria*	95,99	0,56	3310602	-	-	-	+	-	-	-	+	+	+	+	+	+	+	-	-	-	+	-	-	-	-	-	-	-	-	+	-	+	-	-	+	+	+	+	+	+	+	+	+	+	-	-	-	-	-	-	+
N3_9	*Gammaproteobacteria*	96,36	0,56	3375724	-	-	-	+	-	-	-	+	+	+	+	+	+	+	-	-	-	+	-	-	-	-	-	-	-	-	+	-	+	-	-	+	+	+	+	+	+	+	+	+	+	-	-	-	-	-	-	+
N4_5	*Gammaproteobacteria*	93,77	0,93	3261844	-	-	-	+	-	-	-	+	+	+	+	+	+	+	-	-	-	+	-	-	-	-	-	-	-	-	+	-	+	-	-	+	+	+	+	+	+	+	+	+	+	-	-	-	-	-	-	+
N7_24	*Gammaproteobacteria*	85,91	1,14	3031761	-	-	-	+	-	-	-	+	+	+	+	+	+	+	-	-	-	+	-	-	-	-	-	-	-	-	+	-	+	-	-	+	+	+	+	+	+	+	+	+	+	-	-	-	-	-	-	+
N3_45	*Gammaproteobacteria*	96,72	0,74	3690258	-	-	-	+	-	-	-	+	+	+	+	+	+	+	-	-	-	+	-	-	-	-	-	-	-	-	+	-	+	-	-	+	+	+	+	+	+	+	+	+	+	-	-	-	-	-	-	-
N5_41	*Spongiibacteraceae*	94,87	0,37	3679173	+	+	+	+	-	-	-	+	+	+	+	+	+	-	+	-	-	+	+	-	-	-	+	+	+	+	+	+	+	+	-	+	+	+	+	+	+	+	+	+	+	-	-	-	-	-	-	+
N2_11	*Colwellia*	80,68	3,17	2773444	+	+	+	+	-	-	-	+	+	+	+	+	-	+	+	-	-	+	-	-	-	-	+	+	+	+	+	+	+	+	-	+	+	+	+	+	+	+	+	+	+	-	-	-	-	-	-	+
N5_46	*Cellvibrionales*	99,05	0,90	3923773	+	+	+	+	-	-	-	+	+	+	+	+	+	+	+	-	+	+	+	-	-	+	+	+	+	+	+	+	+	+	+	+	+	+	+	+	+	+	+	+	+	+	-	-	+	-	-	+
N5_84	*Gammaproteobacteria*	81,89	7,96	2209996	-	-	-	-	-	-	-	+	+	+	+	-	+	+	-	-	-	+	-	-	-	-	-	-	-	-	+	-	+	-	-	+	-	+	-	+	-	+	+	+	+	-	-	-	-	-	-	-
N2_31	*Porticoccaceae*	61,64	6,54	1594393	-	-	-	-	-	-	-	+	+	+	+	+	+	-	-	-	-	+	-	-	-	-	-	-	-	-	+	-	+	-	-	+	+	+	+	+	+	+	+	+	+	-	-	-	-	-	-	-
N5_79	*Porticoccaceae*	94,81	4,09	2339217	+	-	-	-	-	-	-	+	+	+	+	+	+	-	-	-	-	+	-	-	-	-	-	-	-	-	+	+	+	-	-	+	+	+	+	+	+	+	+	+	+	-	-	-	-	-	-	+
N5_26	*Gammaproteobacteria*	96,3	0,00	2638518	+	-	+	+	-	-	-	+	+	+	+	+	+	+	+	-	-	+	+	-	-	-	+	+	+	+	+	+	+	+	-	+	+	+	+	+	+	+	+	+	+	-	-	-	-	-	-	+
N5_17	*Gammaproteobacteria*	100	8,62	3352294	+	+	-	-	-	+	-	+	+	+	+	+	+	+	-	-	+	+	+	-	-	+	-	+	-	+	+	-	+	-	-	+	+	+	+	+	+	+	+	+	+	-	-	+	+	-	-	+
N2_39	*Gammaproteobacteria*	61,09	6,17	1859633	+	+	-	-	-	+	-	+	+	+	+	+	+	+	-	-	-	+	+	-	-	-	-	+	-	+	+	-	+	-	-	+	+	+	+	+	+	+	+	+	+	-	-	+	-	-	-	+
N6_42	*Proteobacteria*	92,57	1,07	1762173	+	-	-	+	-	-	-	+	-	+	+	+	-	-	-	-	-	+	-	-	-	-	-	-	-	-	+	-	+	-	-	+	+	+	+	+	+	+	-	+	+	-	-	-	-	-	-	+
N3_20	*Betaproteobacteria*	55,23	2,65	959450	+	-	-	+	-	-	-	+	+	+	-	+	+	-	-	-	-	+	+	-	-	-	-	-	-	-	+	-	+	-	-	+	+	+	+	+	+	+	+	+	+	-	-	-	-	-	-	+
N6_31	*Gammaproteobacteria*	94,35	2,02	2157803	+	-	-	+	-	-	-	+	+	+	+	+	+	+	-	-	+	+	+	-	-	-	-	-	-	-	+	-	-	-	-	+	+	+	+	+	+	+	+	+	+	-	-	-	-	-	-	-
N1_44	*Gammaproteobacteria*	92,81	5,84	2284731	+	-	-	+	-	-	-	+	+	+	+	+	+	+	-	-	+	+	+	-	-	-	-	-	-	-	+	-	-	-	-	+	+	+	+	+	+	+	+	+	+	-	-	-	-	-	-	-
N3_36	*Gammaproteobacteria*	86,97	2,09	2033133	+	-	-	+	-	-	-	+	+	+	+	+	+	+	-	-	+	+	+	-	-	-	-	-	-	-	+	-	-	-	-	+	+	+	+	+	+	+	+	+	+	-	-	-	-	-	-	-
N5_37	*Gammaproteobacteria*	89,66	4,51	2105752	+	-	-	+	-	-	-	+	+	+	+	+	+	+	-	-	+	+	+	-	-	-	-	-	-	-	+	-	-	-	-	+	+	+	+	+	+	+	+	+	+	-	-	-	-	-	-	+
N3_31	*Gammaproteobacteria*	97,59	1,96	5297462	+	+	+	+	-	-	-	+	+	+	+	+	+	+	-	-	+	+	+	-	-	-	-	+	+	+	+	+	+	-	-	+	+	+	+	+	+	+	+	+	+	-	-	-	+	-	-	+
N1_68	*Bacteria*	79,87	1,98	1631373	+	+	-	-	-	-	-	+	+	+	+	+	+	-	-	-	-	+	-	-	-	-	-	-	-	-	+	-	-	-	-	+	-	+	+	+	+	+	+	+	+	-	-	-	-	-	-	-
N6_39	*SAR92 clade*	58,62	0,00	2021497	+	-	+	+	-	-	-	+	+	+	+	+	+	-	+	-	-	+	+	-	-	-	+	+	+	+	+	+	+	-	-	+	+	+	+	+	+	+	+	+	+	-	-	-	-	-	-	+
N5_24	*Proteobacteria*	94,26	1,82	3719080	-	-	-	-	-	-	-	+	+	+	+	+	+	-	-	-	+	+	+	-	-	-	-	+	-	+	+	-	+	-	-	+	+	+	+	+	+	+	+	+	+	+	-	-	+	+	-	-
N5_47	*Gammaproteobacteria*	91,51	1,30	1279432	-	-	-	-	-	-	-	+	+	+	+	+	-	+	-	-	-	+	-	-	-	-	-	-	-	-	+	-	+	-	-	+	+	+	+	+	+	+	+	+	+	-	-	-	-	-	-	-
N1_10	*Gammaproteobacteria*	90,65	2,04	1356332	-	-	-	-	-	-	-	+	+	+	+	+	-	+	-	-	-	+	-	-	-	-	-	-	-	-	+	-	+	-	-	+	+	+	+	+	+	+	+	+	+	-	-	-	-	-	-	-
N5_75	*Gammaproteobacteria*	97,84	1,08	5300521	-	-	-	+	-	-	-	+	+	+	+	+	+	+	+	-	-	+	-	-	-	-	+	-	-	-	+	+	+	-	-	+	+	+	+	+	+	+	+	+	+	-	-	-	-	-	-	-
N7_2	*Paraperlucidibaca baekdonensis*	98,01	0,25	2474824	+	-	+	+	-	-	-	+	+	+	+	+	+	-	+	-	-	+	-	-	-	-	+	+	+	+	+	+	-	-	-	+	+	+	+	+	+	+	+	+	+	-	-	-	-	-	-	+
N4_3	*Paraperlucidibaca baekdonensis*	98,01	0,25	2474821	+	-	+	+	-	-	-	+	+	+	+	+	+	-	+	-	-	+	-	-	-	-	+	+	+	+	+	+	-	-	-	+	+	+	+	+	+	+	+	+	+	-	-	-	-	-	-	+
N8_7	*Paraperlucidibaca baekdonensis*	50,42	0,00	955062	+	-	+	+	-	-	-	+	-	+	+	+	-	-	-	-	-	+	-	-	-	-	-	+	+	+	+	+	-	-	-	+	+	+	+	-	+	+	-	+	+	-	-	-	-	-	-	+
N6_1	*Halomonas sp, KO116*	100	0,93	4382077	+	-	-	-	-	-	-	+	+	+	+	+	+	+	-	-	+	+	+	-	-	+	-	-	-	-	+	-	+	-	+	+	+	+	+	+	+	+	+	+	+	+	-	+	+	+	+	+
N1_37	*Gammaproteobacteria*	83,02	9,75	3018370	+	-	-	+	-	-	-	+	+	+	+	+	+	+	-	-	+	+	+	-	-	-	-	-	-	-	+	+	+	-	-	+	+	+	+	+	+	+	+	+	+	-	+	+	+	+	-	+
N5_72	*Gammaproteobacteria*	97,56	0,61	3392055	+	+	-	+	-	-	+	+	+	+	+	+	+	+	+	+	+	+	+	+	+	+	+	-	-	-	+	+	+	+	+	+	+	+	+	+	+	+	+	+	+	+	+	+	+	-	+	-
N5_30	*Bacteroidetes*	88,2	0,55	2053937	-	-	-	-	-	-	-	+	+	+	+	+	-	-	-	-	-	+	-	-	-	-	-	-	-	-	-	-	-	-	-	+	+	+	+	+	+	+	-	+	+	-	-	-	-	+	-	+
N2_27	*Bacteroidetes*	96,43	0,48	2564131	-	-	-	-	-	-	-	+	+	+	+	+	-	-	-	-	-	+	-	-	-	-	-	-	-	-	-	-	-	-	-	+	+	+	+	+	+	+	-	+	+	-	-	-	-	+	-	+
N5_66	*Doktonia pacifica*	97,88	3,76	4952315	-	-	+	+	-	-	-	+	+	+	+	+	+	-	+	-	+	+	+	-	-	-	+	+	+	+	-	-	-	-	-	+	+	+	+	+	+	+	+	+	+	-	-	-	+	+	-	-
N1_65	*Flavobacteriales*	92,74	1,36	1459003	-	-	-	-	-	-	-	+	-	+	+	+	-	-	-	-	-	+	-	-	-	-	-	-	-	-	-	-	-	-	-	+	+	-	+	-	+	-	-	+	+	-	-	-	-	-	-	-
N1_15	*Bacteria*	87,66	3,30	1985634	-	-	-	-	-	-	-	+	+	+	-	+	+	-	-	-	-	+	+	-	-	-	-	-	-	-	-	-	-	-	-	+	+	+	+	+	+	+	+	+	+	-	-	-	-	-	-	-

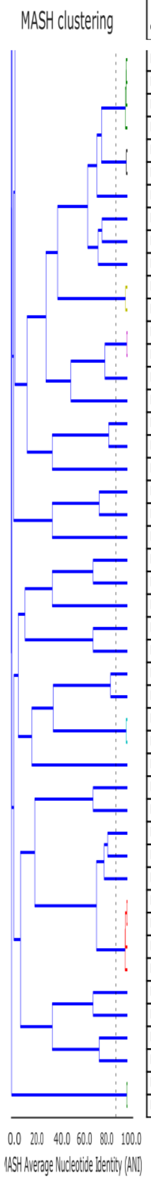

MAG code	MAG affiliation (Kaiju)	Completeness	Contamination	Genome size (bp)	*almA*	*ladA*	*MAH1*	*alkB1_2*	*bmoBCDXYZ*	*pmoABC*	*prmABCD*	*rubB*	*α-adh* (K00055)	*ped* (K14746)	*PHE* (K03380)	*xylC*	*ADH1* (K18857)	*bsdCD*	*cymAab*	*dmpKLMNOP*	*etbAabc*	*hcaB*	*hcaCDEF*	*phcCF*	*tmoABCDEF*	*todABC1C2*	*xylAM*	*CYP1A1A2*	*CYP2A6*	*CYP3A4*	*gst* (K00799)	*GSTK1* (K13299)	*HPGDS*	*nahC*	*nidAB*	*nahB*	*phdK*	*adh* (K00001)	*adhE* (K04072)	*adhP* (K13953)	*ALDH* (K00128)	*chnB*	*frmA*	*phdE*	*sdh* (K00480)	*dbfA1A2*	*ligAB*	*nagGH*	*nahAabcd*	*pcaGH*	*phdIJ*	*yaiY*
N4_26	*Ulvibacter litoralis*	99,24	0,57	3547593	-	-	+	+	-	-	-	+	+	+	+	+	+	-	+	-	-	+	-	-	-	-	+	+	+	+	-	-	-	-	-	+	+	+	+	+	+	+	+	+	+	-	-	-	-	-	-	+
N3_21	*Ulvibacter litoralis*	94,83	3,14	2914506	-	-	+	-	-	-	-	+	+	+	+	+	+	-	-	-	-	+	-	-	-	-	-	+	+	+	-	-	-	-	-	+	+	+	+	+	+	+	+	+	+	-	-	-	-	-	-	+
N8_2	*Ulvibacter*	89,66	9,80	3283242	-	-	+	-	-	-	-	+	+	+	+	+	-	-	-	-	-	+	-	-	-	-	-	+	+	+	-	-	-	-	-	+	+	+	+	+	+	+	+	+	+	-	-	-	-	-	-	+
N7_25	*Ulvibacter litoralis*	60,34	0,00	2135277	-	-	-	-	-	-	-	+	-	+	+	+	-	-	-	-	-	+	-	-	-	-	-	-	-	-	-	-	-	-	-	+	+	-	+	-	+	+	-	+	+	-	-	-	-	-	-	+
N6_29	*Flavobacteriaceae*	99,01	0,77	3097952	+	-	+	-	-	-	-	+	+	+	+	+	-	-	-	-	-	+	-	-	-	-	-	+	+	+	-	-	-	-	-	+	+	+	+	+	+	+	+	+	+	-	-	-	-	-	-	+
N3_29	*Flavobacteriaceae*	97,69	3,04	3069083	+	-	+	-	-	-	-	+	+	+	+	+	-	-	-	-	-	+	-	-	-	-	-	+	+	+	-	-	-	-	-	+	+	+	+	+	+	+	+	+	+	-	-	-	-	-	-	+
N2_45	*Doktonia*	52,59	0,00	1905872	-	-	-	-	-	-	-	+	-	+	+	+	-	-	-	-	-	+	-	-	-	-	-	-	-	-	-	-	-	-	-	+	+	-	+	-	+	+	-	+	+	-	-	-	-	+	-	-
N6_20	*Flavobacteriaceae*	98,57	0,38	3688655	-	-	+	+	-	-	-	+	+	+	+	+	+	-	+	-	-	+	-	-	-	-	+	+	+	+	-	-	-	-	-	+	+	+	+	+	+	-	+	+	+	-	-	-	-	-	-	+
N3_3	*Flavobacteriaceae*	68,97	1,72	3950076	-	-	-	-	-	-	-	+	+	+	+	+	+	-	-	-	-	+	-	-	-	-	-	-	-	-	-	-	-	-	-	+	+	+	+	+	+	-	+	+	+	-	-	-	-	-	-	+
N7_5	*Aequorivita*	91,26	4,63	3240517	-	-	+	-	-	-	-	+	+	+	+	+	+	-	-	-	-	+	-	-	-	-	-	+	+	+	-	-	-	-	-	+	+	+	+	+	+	+	+	+	+	-	-	-	-	-	-	+
N5_25	*Flavobacteriaceae*	72,39	1,86	3401147	-	-	+	-	-	-	-	+	+	+	+	+	+	-	-	-	+	+	+	-	-	-	-	+	+	+	-	-	-	-	-	+	+	+	+	+	+	-	+	+	+	-	-	-	+	-	-	+
N2_7	*Maribacter antarcticus*	73,01	0,54	2925499	-	-	+	+	-	-	-	+	+	+	+	+	+	-	+	-	+	+	+	-	-	-	+	+	+	+	-	-	-	-	-	+	+	+	+	+	+	-	+	+	+	-	-	-	+	-	-	+
N5_6	*Flavobacteriaceae*	94,6	4,65	2390527	+	+	-	+	-	-	-	+	+	+	+	+	-	-	+	-	-	+	+	-	-	-	+	-	-	-	-	-	-	-	-	+	+	+	+	+	+	+	+	+	+	-	-	-	+	-	-	-
N2_37	*Flavobacteriaceae*	95,32	3,08	2397404	+	+	-	+	-	-	-	+	+	+	+	+	-	-	+	-	-	+	+	-	-	-	+	-	-	-	-	-	-	-	-	+	+	+	+	+	+	+	+	+	+	-	-	-	+	-	-	-
N5_40	*Flavobacteriaceae*	95,66	7,78	3278340	-	-	-	-	-	-	-	+	+	+	+	+	+	-	-	-	+	+	+	-	-	-	-	-	-	-	-	-	-	-	-	+	+	+	+	+	+	-	+	+	+	-	-	-	+	+	-	+
N5_63	*Bacteroidetes*	98,81	1,19	1274867	-	-	-	-	-	-	-	+	+	+	+	+	+	-	-	-	-	+	-	-	-	-	-	-	-	-	+	+	-	-	-	+	+	+	+	+	+	+	+	+	+	-	-	-	-	-	-	+
N6_24	*Polaribacter*	75,44	8,77	2617131	-	-	-	-	-	-	-	+	+	+	+	+	-	-	-	-	-	+	-	-	-	-	-	-	-	-	-	-	-	-	-	+	+	+	+	+	+	+	+	+	+	-	-	-	-	+	-	-
N1_61	*Polaribacter*	57,35	2,07	1148554	-	-	-	-	-	-	-	+	+	+	+	+	-	-	-	-	-	+	-	-	-	-	-	-	-	-	-	-	-	-	-	+	+	+	+	+	+	+	+	+	+	-	-	-	-	-	-	-
N1_70	*Gammaproteobacteria*	61,87	5,50	2310463	+	+	+	+	-	-	-	+	+	+	+	+	+	-	-	-	+	+	+	-	-	+	-	+	+	+	+	+	+	-	-	+	+	+	+	+	+	+	+	+	+	-	-	-	+	-	-	+
N5_43	*Proteobacteria*	95,09	1,79	3961459	-	-	+	+	-	-	-	+	+	+	+	+	+	-	+	-	+	+	+	-	-	-	+	+	+	+	+	-	+	-	-	+	+	+	+	+	+	+	+	+	+	-	-	-	+	+	-	+
N5_16	*Proteobacteria*	78,95	7,97	3580505	-	-	+	+	-	-	-	+	+	+	+	+	+	-	+	-	+	+	+	-	-	-	+	+	+	+	+	-	+	-	-	+	+	+	+	+	+	+	+	+	+	-	-	-	+	-	-	+
N1_62	*Planctomycetes*	76,7	8,11	1607967	-	-	-	-	-	-	-	+	+	+	+	+	+	+	-	-	-	+	-	-	-	-	-	-	-	-	+	-	-	-	-	+	+	+	+	+	+	-	+	+	+	-	-	-	-	-	-	-
N2_42	*Proteobacteria*	91,61	1,61	7164090	+	-	+	-	-	-	-	+	+	+	+	+	+	+	-	-	+	+	+	-	-	-	-	+	+	+	+	+	+	-	-	+	+	+	+	+	+	+	+	+	+	-	-	-	-	+	-	+
N2_30	*Deltaproteobacteria*	93,87	2,15	8502960	+	-	+	-	-	-	-	+	+	+	+	+	+	-	-	-	+	+	+	-	-	-	-	+	+	+	+	+	+	-	-	+	+	+	+	+	+	+	+	+	+	-	-	-	-	+	-	+
N5_74	*Bacteria*	94,6	4,84	10217815	+	+	+	-	-	-	-	+	+	+	-	+	+	-	-	-	+	+	+	-	-	+	-	+	+	+	+	+	+	-	-	+	+	+	+	+	+	+	+	+	+	-	-	-	+	+	-	-
N1_48	*PVC group*	66,35	5,60	4443651	+	+	-	-	-	-	-	+	+	+	+	+	+	+	-	-	-	+	-	-	-	-	-	-	-	-	+	-	-	-	-	+	+	+	+	+	+	+	+	+	+	-	-	-	-	-	-	+
N1_32	*Bacteria*	85,64	1,10	2053734	-	+	-	-	-	-	-	+	+	+	-	+	+	+	-	-	-	+	-	-	-	-	-	-	-	-	-	-	-	-	-	+	-	+	+	+	+	+	+	+	+	-	-	-	-	-	+	+
N3_25	*UC*	97,73	1,14	6480998	-	-	-	-	-	-	-	+	+	+	+	+	+	+	-	-	-	+	-	-	-	-	-	-	-	-	-	-	-	-	-	+	+	+	+	+	+	-	+	+	+	-	-	-	-	+	-	+
N1_63	*Bacteria*	95,39	0,00	4900825	-	-	-	-	-	-	-	+	+	+	-	+	+	+	-	-	-	+	-	-	-	-	-	-	-	-	-	-	-	-	-	+	+	+	+	+	+	-	+	+	+	-	-	-	-	+	-	+
N5_78	*Planctomycetes*	75,37	0,60	5962533	-	-	-	-	-	-	-	+	+	+	-	+	+	+	-	-	+	+	+	-	-	-	-	-	-	-	-	-	-	-	-	+	+	+	+	+	+	+	+	+	+	-	-	-	+	-	-	-
N2_24	*Planctomycetes*	95,7	1,08	7367198	-	-	+	-	-	-	-	+	+	+	+	+	+	+	-	-	+	+	+	-	-	-	-	+	+	+	+	-	+	-	-	+	+	+	+	+	+	+	+	+	+	-	-	-	+	-	-	-
N1_58	*Planctomycetes*	94,62	0,00	3562003	-	-	-	-	-	-	-	+	+	+	+	+	+	+	-	-	-	+	-	-	-	-	-	-	-	-	-	-	-	-	-	+	+	+	+	+	+	+	+	+	+	-	-	-	-	-	-	+
N5_19	*PVC group*	91,81	2,20	6604528	+	-	+	+	-	-	-	+	+	+	+	+	+	-	-	-	-	+	-	-	-	-	-	+	+	+	-	-	-	-	-	+	+	+	+	+	+	+	+	+	+	-	-	-	-	+	+	+
N1_2	*Verrucomicrobia*	61,52	1,15	1691887	-	-	-	-	-	-	-	+	+	+	+	+	+	+	-	-	-	+	-	-	-	-	-	-	-	-	-	-	-	-	-	+	+	+	+	+	+	-	+	+	+	-	-	-	-	+	-	+
N5_68	*Bacteria*	75,44	3,60	1806223	-	-	-	-	-	-	-	+	+	+	-	+	+	+	-	-	-	+	-	-	-	-	-	-	-	-	-	-	-	-	-	+	+	+	+	+	+	-	+	+	-	-	-	-	-	-	-	-
N2_19	*Bacteria*	78,32	1,27	2467512	-	-	-	-	-	-	-	+	+	+	+	+	+	+	-	-	-	+	-	-	-	-	-	-	-	-	-	-	-	-	-	+	+	+	+	+	+	-	+	+	+	-	-	-	-	-	-	+
N5_56	*Bacteria*	74,77	1,70	2189201	-	-	-	-	-	-	-	+	+	+	-	+	+	-	-	-	-	+	-	-	-	-	-	-	-	-	-	-	-	-	-	+	+	+	+	+	+	-	+	+	+	-	-	-	-	-	-	-
N6_3	*Bacteria*	92,05	1,14	2804083	-	-	-	-	-	-	-	+	+	+	+	+	+	+	-	-	-	+	-	-	-	-	-	-	-	-	-	-	-	-	-	+	+	+	+	+	+	+	+	+	+	-	-	-	-	-	-	-
N5_10	*Bacteria*	90,91	0,00	2797024	-	-	-	-	-	-	-	+	+	+	-	+	+	+	-	-	-	+	-	-	-	-	-	-	-	-	-	-	-	-	-	+	+	+	+	+	+	+	+	+	+	-	-	-	-	-	-	-
N2_34	*Bacteria*	92,05	1,14	2857624	-	-	-	-	-	-	-	+	+	+	+	+	+	+	-	-	-	+	-	-	-	-	-	-	-	-	-	-	-	-	-	+	+	+	+	+	+	+	+	+	+	-	-	-	-	-	-	-
N3_38	*Pseudomonas*	97,73	1,14	3154249	-	-	-	-	-	-	-	+	+	+	+	+	+	+	-	-	-	+	-	-	-	-	-	-	-	-	-	-	-	-	-	+	+	+	+	+	+	+	+	+	+	-	-	-	-	-	-	-
N1_52	*Ca. Poseidoniales archaeon*	80,4	2,13	1479766	-	-	-	+	-	-	-	+	+	+	+	+	+	+	-	-	-	+	-	-	-	-	-	-	-	-	+	-	+	-	-	+	+	+	+	+	+	-	+	+	+	-	-	-	-	-	-	-
N1_11	*Ca. Poseidoniales archaeon*	71,6	0,80	1400458	-	-	-	-	-	-	-	+	+	+	+	+	+	+	-	-	-	+	+	-	-	-	-	-	-	-	-	-	-	-	-	+	+	+	+	+	+	-	+	+	+	-	-	-	+	-	-	-
N1_82	*Ca, Poseidoniales archaeon*	82,93	2,40	1799778	-	-	-	+	-	-	-	+	+	+	+	+	+	+	-	-	+	+	+	-	-	-	+	-	-	-	+	-	+	-	-	+	+	+	+	+	+	+	+	+	+	-	-	-	-	-	-	-
N1_60	*Euryarchaeota*	64,4	5,73	1283264	-	-	-	+	-	-	-	+	+	+	+	+	+	+	-	-	-	+	-	-	-	-	+	-	-	-	-	-	-	-	-	+	+	+	+	+	+	-	+	+	+	-	-	-	-	-	-	-
N8_14	*Alphaproteobacteria*	82,17	0,87	2940151	+	-	-	-	-	-	-	+	+	+	+	+	+	+	-	-	-	+	-	-	-	-	-	-	-	-	+	+	+	-	-	+	+	+	+	+	+	+	+	+	+	-	-	-	-	-	-	+
N7_18	*Alphaproteobacteria*	97,39	0,87	3440625	+	-	-	-	-	-	-	+	+	+	+	+	+	+	-	-	-	+	-	-	-	-	-	-	-	-	+	+	+	-	-	+	+	+	+	+	+	+	+	+	+	-	-	-	-	-	-	+

Table S22. Major operational taxonomic units (OTUs) of bacterial and archaeal genera found in seawater (SW), oil contaminated seawater (SWO), and biostimulated oil contaminated seawater (SWOB) after four and eight months of incubation according to amplicon-based sequencing. Multiple hits within the same genera are indicated in brackets after the genera name.

**Treat-ment type**	***Phylum***	***Class***	***Order***	***Family***	***Genus***	**4 months**	**8 months**
**SW**	*Bacteroidetes*	*Bacteroidia*	*Flavobacteriales*	*Crocinitomicaceae*	*Unclassified Crocinitomicaceae*	+	-
				Flavobacteriaceae	*Doktonia*	+	+
					Ulvibacter	+	-
					Unclassified Flavobacteriacea	+	+
				NS9 marine group	*NS9 marine group genus*	+	-
			Sphingobacteriales	*NS11-12 marine group*	*NS11-12 marine group genus*	+	+
	Firmicutes	*Clostridia*	*Clostridiales*	*Clostridiaceae*	*Clostridium (sensu stricto)*	+	-
	Planctomycetes	*OM190*			*OM190 genus*	+	+
		Phycisphaerae	*Phycisphaerales*	*Phycisphaeraceae*	*SM1A02*	+	+
	Proteobacteria	*α-proteobacteria*	*Kordiimonadales*	*Uncultured Kordiimonadales*		+	-
			Rhizobiales	*Rhizobiaceae*	*Lentilitoribacter*	+	+
			Rhodobacterales	*Rhodobacteraceae*	*Planktomarina*	+	-
					Pseudophaeobacter	+	+
					Unclassified Rhodobacteraceae (2)	+	+
					Sedimentitaleae	+	+
			Rhodospirillales	*Magnetospiraceae*	*Uncultured Magnetospiraceae*	+	+
			SAR11 clade	*Clade III*	*Clade III genus*	+	+
		γ-proteobacteria	*Betaproteobacteriales*	*Methylophilaceae*	*OM43_clade*	+	+
			Cellvibrionales	*Porticoccaceae*	*Porticoccus*	+	+
				Spongiibacteraceae	*Zhongshania*	+	+
				Unclassified Cellvibrionales		+	+
		δ-proteobacteria	*Bdellovibrionales*	*Bdellovibrionaceae*	*OM27_clade*	+	-
			Myxococcales	*Sandaracinaceae*	*Uncultured Sandaracinaceae*	+	-
**SWO**	*Bacteroidetes*	*Bacteroidia*	*Flavobacteriales*	*Flavobacteriaceae*	*Cellulophaga*	+	-
					Maribacter (2)	+	+

**Treat-ment type**	***Phylum***	***Class***	***Order***	***Family***	***Genus***	**4 months**	**8 months**
					Ulvibacter	+	-
					Unclassified Flavobacteriaceae (2)	+	+
			Parvibaculales	*Parvibaculaceae*	*Parvibaculum*	+	+
		α-proteobacteria	*Rhodobacterales*	*Rhodobacteriaceae*	*Pacificibacter*	+	+
	Proteobacteria		*Rhodovibrionales*	*Kiloniellaceae*	*Pelagibius*	-	+
		γ-proteobacteria	*Alteromonadales*	*Alteromonadaceae*	*Colwellia*	-	+
			Oceanospirillales	*Pseudohongiellaceae*	*Pseudohongiella*	+	+
	Thaumarchaeota	*Nitrososphaeria*	*Nitrosopumilales*	*Nitrosopumiliaceae*	*Ca. Nitrosopumilus*	+	-
	Bacteroidetes	*Bacteroidia*	*Flavobacteriales*	*Flavobacteriaceae*	*Aequorivita*	+	+
		α-proteobacteria	*Rhodobacterales*	*Rhodobacteriaceae*	*Unclassified Rhodobacteraceae*	+	+
			Sphingomonadales	*Sphingomonadaceae*	*Unclassified Sphingomonadaceae*	+	+
**SWOB**	*Proteobacteria*		*Alteromonadales*	*Alteromonadaceae*	*Paraglaciecola*	+	+
		γ-proteobacteria	*Pseudomonadales*	*Moraxellaceae*	*Paraperlucidibaca*	+	+
				Pseudomonadaceae	*Pseudomonas (2)*	+	+
					Tenacibaculum	+	-
	Bacteroidetes	*Bacteroidia*	*Flavobacteriales*	*Flavobacteriaceae*	*Ulvibacter (2)*	+	+ (SW)
					Unclassified Flavobacteriaceae (2)	+	+
	Planctomycetes	*OM190*			*OM190 genus*	+	+ (SW)
					Algimonas	+	+ (SW)
			Caulobacterales	*Hyphomonadaceae*	*Hyphomonas*	+	+ (SWO)
		α-proteobacteria			*Maricaulis*	+	+ (SW)
**SW & SWO**			*Rhodobacterales*	*Rhodobacteriaceae*	*Unclassified Rhodobacteraceae (2)*	+	-
	Proteobacteria		*Rhodospirillales*	*Terasakiellaceae*	*Uncultured Terasakiellaceae*	+	+ (SW)
			SAR11 clade	*Clade I*	*Clade Ia genus*	+	-
				Alteromonadaceae	*Paraglaciecola*	+	+
		γ-proteobacteria	*Alteromonadales*	*Colwelliaceae*	*Colwellia*	+ (SW)	+
					Uncultured Colwelliaceae	-	+
			Nitrosococcales	*Methylophagaceae*	*Uncultured Methylophagaceae*	+	+
**SW &**	*Acidobacteria*	*Subgroup 6*			*Subgroup 6 genus (2)*	+ (SW)	+
	Bacteroidetes	*Bacteroidia*	*Flavobacteriales*	*Flavobacteriaceae*	*Flavobacterium*	+	+

Treat-ment type	*Phylum*	*Class*	*Order*	*Family*	*Genus*	4 months	8 months
	Proteobacteria	*α-proteobacteria*	*Unclassified α-proteobacteria*			-	+
	Proteobacteria	*γ-proteobacteria*	*Methylococcales*	*Cycloclasticaceae*	*Cycloclasticus*	+	+
**SWO & SWOB**	*Proteobacteria*	*α-proteobacteria*	*Sneathiellales*	*Sneathiellaceae*	*Sneathiella*	+	+
**SWO & SWOB**	*Proteobacteria*	*γ-proteobacteria*	*Oceanospirillales*	*Marinomonadaceae*	*Marinomonas*	+	+
**SWO & SWOB**	*Proteobacteria*	*γ-proteobacteria*	*Oceanospirillales*	*Nitrincolaceae*	*Amphritea*	+	+ (SWOB)
**SW & SWO & SWOB**	*Bacteroidetes*	*Bacteroidia*	*Flavobacteriales*	*Flavobacteriaceae*	*Aequorivita*	+ (SWO. SWOB)	+
**SW & SWO & SWOB**	*Bacteroidetes*	*Bacteroidia*	*Flavobacteriales*	*Flavobacteriaceae*	*Aurantivirga*	+	+
**SW & SWO & SWOB**	*Bacteroidetes*	*Bacteroidia*	*Flavobacteriales*	*Flavobacteriaceae*	*Jejudonia*	+ (SWO)	+
**SW & SWO & SWOB**	*Bacteroidetes*	*Bacteroidia*	*Flavobacteriales*	*Flavobacteriaceae*	*Ulvibacter*	+	+
**SW & SWO & SWOB**	*Bacteroidetes*	*Bacteroidia*	*Flavobacteriales*	*Flavobacteriaceae*	*Unclassified Flavobacteriaceae (3)*	+	+
**SW & SWO & SWOB**	*Planctomycetes*	*Phycisphaerae*	*Phycisphaerales*	*Phycisphaeraceae*	*SM1A02*	+ (SW. SWO)	+
**SW & SWO & SWOB**	*Proteobacteria*	*α-proteobacteria*	*Rhizobiales*	*Rhizobiaceae*	*Hoeflea*	+ (SW. SWO)	+
**SW & SWO & SWOB**	*Proteobacteria*	*α-proteobacteria*	*Rhodobacterales*	*Rhodobacteraceae*	*Pacificibacter*	+	+
**SW & SWO & SWOB**	*Proteobacteria*	*α-proteobacteria*	*Rhodobacterales*	*Rhodobacteraceae*	*Unclassified Rhodobacteraceae*	+	+
**SW & SWO & SWOB**	*Proteobacteria*	*α-proteobacteria*	*Sneathiellales*	*Sneathiellaceae*	*Sneathiella*	+	+
**SW & SWO & SWOB**	*Proteobacteria*	*α-proteobacteria*	*Sphingomonadales*	*Sphingomonadaceae*	*Sphingorhabdus*	+	+
**SW & SWO & SWOB**	*Proteobacteria*	*γ-proteobacteria*	*Alteromonadales*	*Colwelliaceae*	*Colwellia (2)*	+	+
**SW & SWO & SWOB**	*Proteobacteria*	*γ-proteobacteria*	*Enterobacteriales*	*Enterobacteraceae*	*Unclassified Enterobacteraceae*	-	+
**SW & SWO & SWOB**	*Proteobacteria*	*γ-proteobacteria*	*Methylococcales*	*Cycloclasticaceae*	*Cycloclasticus*	+	+
**SW & SWO & SWOB**	*Proteobacteria*	*γ-proteobacteria*	*Oceanospirillales*	*Marinomonadaceae*	*Marinomonas*	+	+
**SW & SWO & SWOB**	*Proteobacteria*	*γ-proteobacteria*	*Oceanospirillales*	*Nitrincolaceae*	*Uncultured Nitrincolaceae*	+	+
**SW & SWO & SWOB**	*Proteobacteria*	*γ-proteobacteria*	*Oceanospirillales*	*Saccharospirillaceae*	*Oleispira*	+	-
**SW & SWO & SWOB**	*Proteobacteria*	*γ-proteobacteria*	*Pseudomonadales*	*Moraxellaceae*	*Paraperlucidibaca*	+ (SW. SWOB)	+
**SW & SWO & SWOB**	*Proteobacteria*	*γ-proteobacteria*	*Unclassified Gammaproteobacteria*			+	+

Supplementary figures

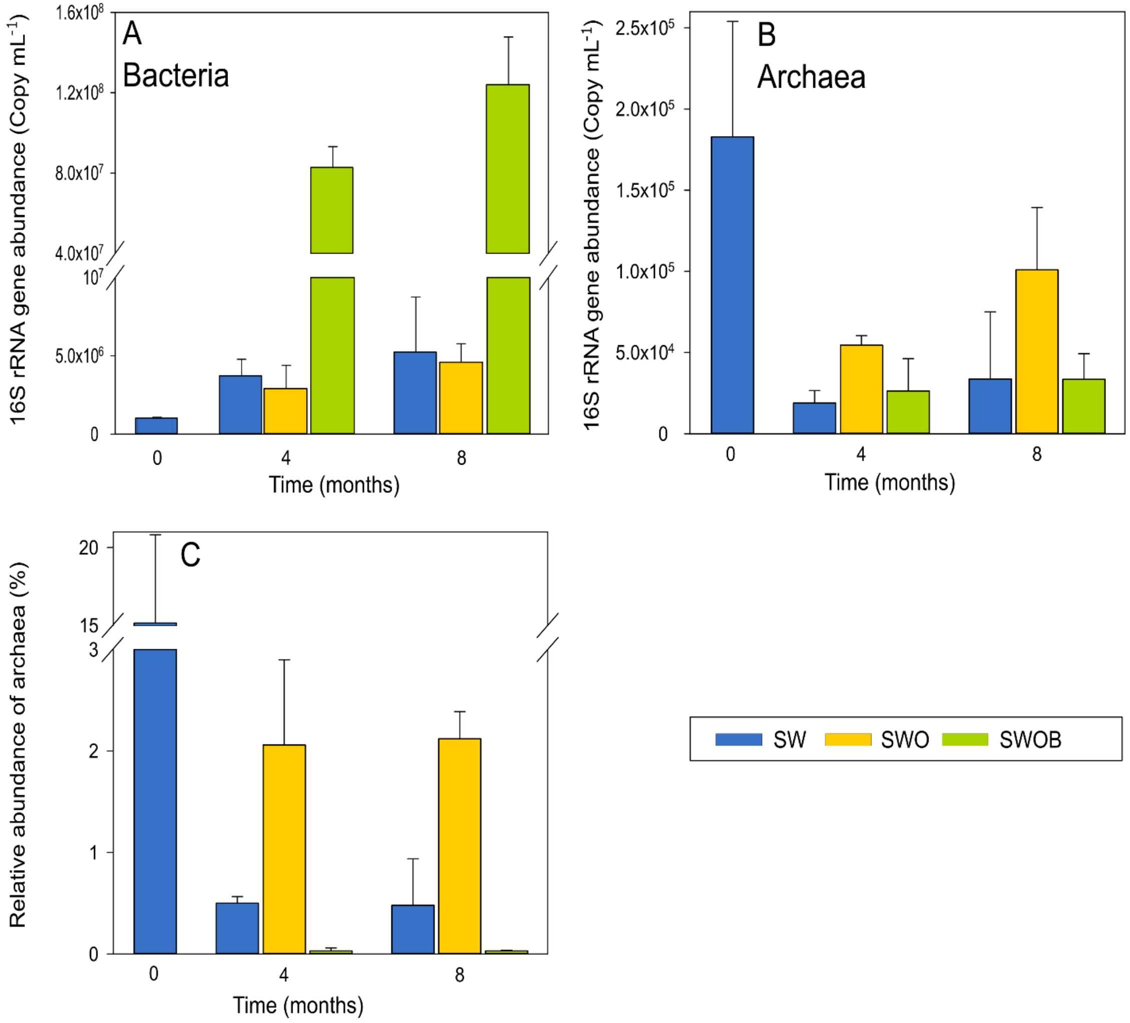

Fig. S1. The abundances of bacterial (A) and archaeal (B) communities, as well as the relative abundance of the archaeal community (C) in seawater (SW), oil contaminated seawater (SWO) and biostimulated oil contaminated seawater (SWOB), *n = 2*.

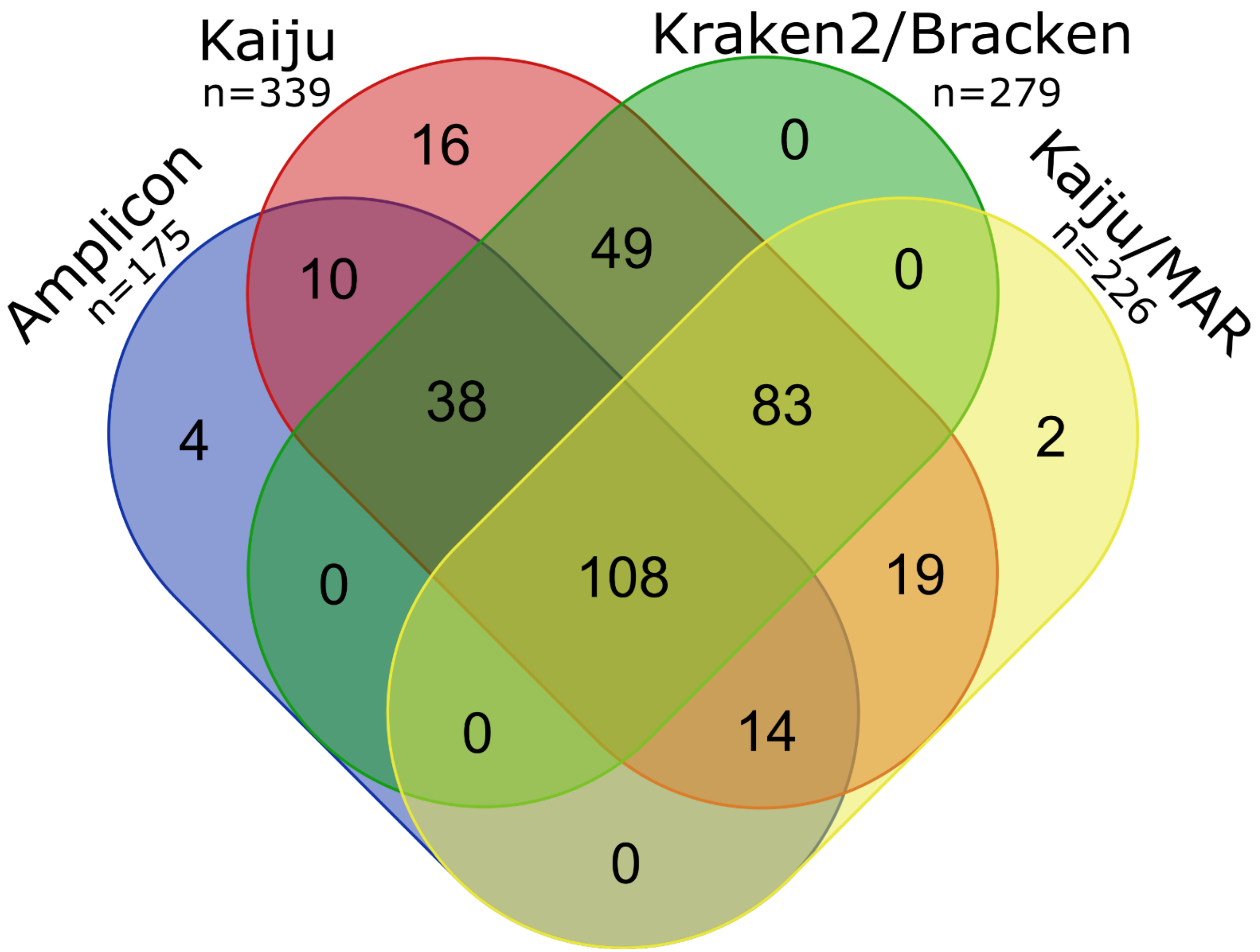

Fig. S2. Venn diagram showing the overlap of detected genera containing oil hydrocarbon degraders between different taxonomic classification methods (Kaiju with the NCBI-nr database, Kaiju with the MAR$_{DB}$ database, Kraken2 with the Standard Kraken 2 database, Bracken with the Standard Kraken 2 database, and amplicon-based sequencing with the SILVA database).

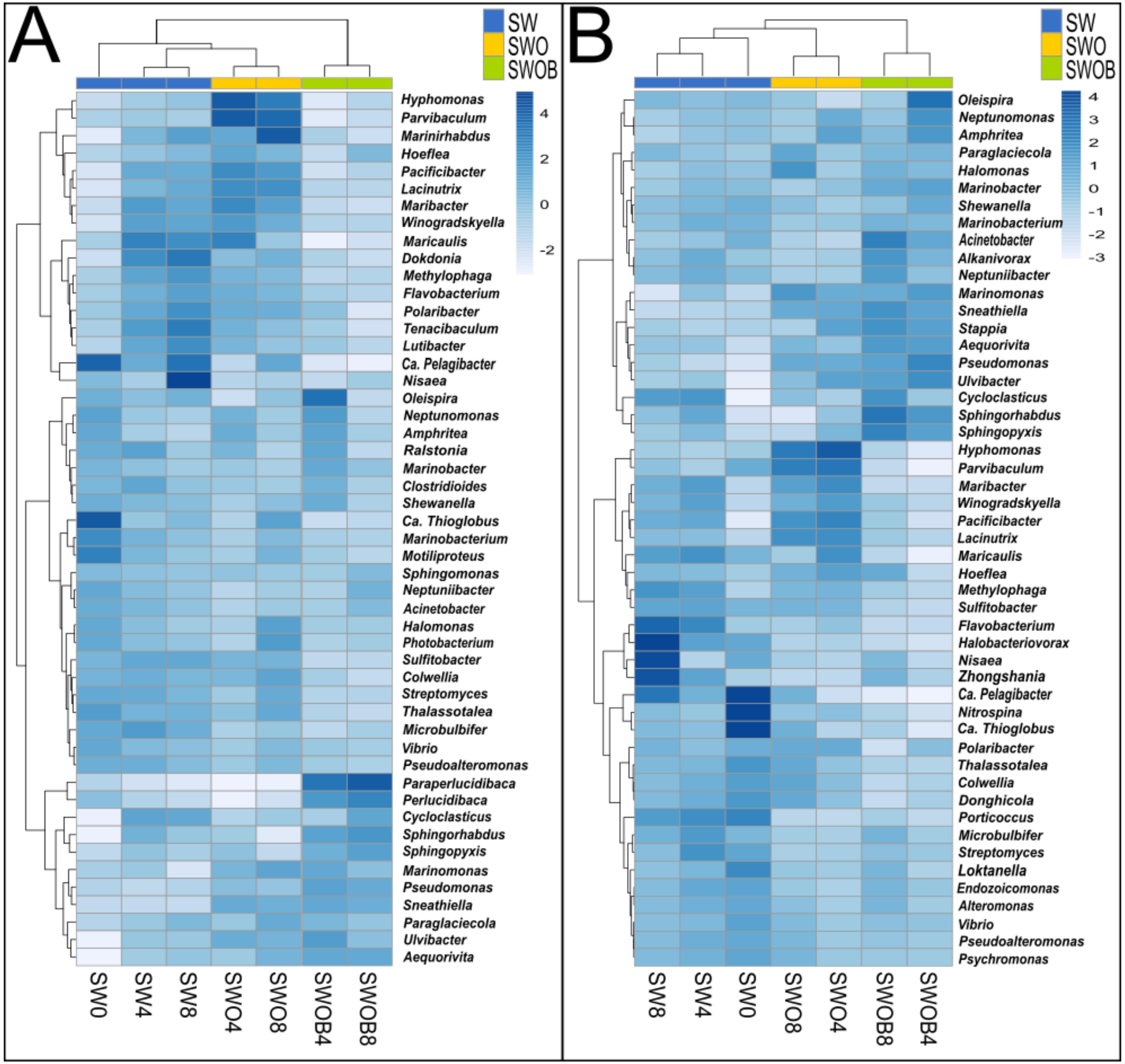
A
SW
SWO
SWOB
4
2
0
-2
Hyphomonas
Parvibaculum
Marinirhabdus
Hoeflea
Pacificibacter
Lacinutrix
Maribacter
Winogradskyella
Maricaulis
Dokdonia
Methylophaga
Flavobacterium
Polaribacter
Tenacibaculum
Lutibacter
Ca. Pelagibacter
Nisaea
Oleispira
Neptunomonas
Amphritea
Ralstonia
Marinobacter
Clostridioides
Shewanella
Ca. Thioglobus
Marinobacterium
Motiliproteus
Sphingomonas
Neptuniibacter
Acinetobacter
Halomonas
Photobacterium
Sulfitobacter
Colwellia
Streptomyces
Thalassotalea
Microbulbifer
Vibrio
Pseudoalteromonas
Paraperlucidibaca
Perlucidibaca
Cycloclasticus
Sphingorhabdus
Sphingopyxis
Marinomonas
Pseudomonas
Sneathiella
Paraglaciecola
Ulvibacter
Aequorivita
SW0
SW4
SW8
SWO4
SWO8
SWOB4
SWOB8
B
SW
SWO
SWOB
4
3
2
1
0
-1
-2
-3
Oleispira
Neptunomonas
Amphritea
Paraglaciecola
Halomonas
Marinobacter
Shewanella
Marinobacterium
Acinetobacter
Alkanivorax
Neptuniibacter
Marinomonas
Sneathiella
Stappia
Aequorivita
Pseudomonas
Ulvibacter
Cycloclasticus
Sphingorhabdus
Sphingopyxis
Hyphomonas
Parvibaculum
Maribacter
Winogradskyella
Pacificibacter
Lacinutrix
Maricaulis
Hoeflea
Methylophaga
Sulfitobacter
Flavobacterium
Halobacteriovorax
Nisaea
Zhongshania
Ca. Pelagibacter
Nitrospina
Ca. Thioglobus
Polaribacter
Thalassotalea
Colwellia
Donghicola
Porticoccus
Microbulbifer
Streptomyces
Loktanella
Endozoicomonas
Alteromonas
Vibrio
Pseudoalteromonas
Psychromonas
SW8
SW4
SW0
SWO8
SWO4
SWOB8
SWOB4

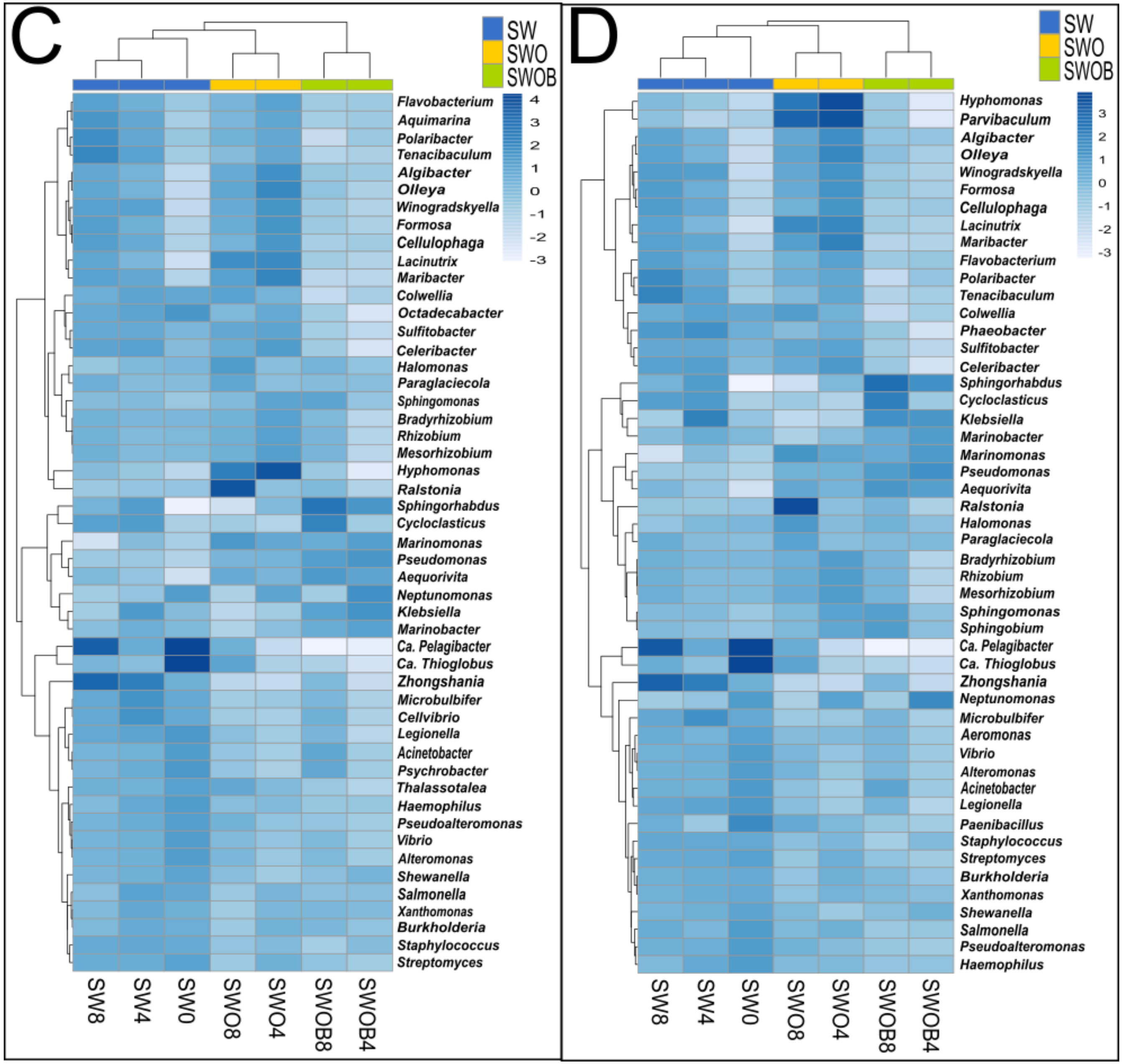
C
SW
SWO
SWOB
4
3
2
1
0
-1
-2
-3
Flavobacterium
Aquimarina
Polaribacter
Tenacibaculum
Algibacter
Olleya
Winogradskyella
Formosa
Cellulophaga
Lacinutrix
Maribacter
Colwellia
Octadecabacter
Sulfitobacter
Celeribacter
Halomonas
Paraglaciecola
Sphingomonas
Bradyrhizobium
Rhizobium
Mesorhizobium
Hyphomonas
Ralstonia
Sphingorhabdus
Cycloclasticus
Marinomonas
Pseudomonas
Aequorivita
Neptunomonas
Klebsiella
Marinobacter
Ca. Pelagibacter
Ca. Thioglobus
Zhongshania
Microbulbifer
Cellvibrio
Legionella
Acinetobacter
Psychrobacter
Thalassotalea
Haemophilus
Pseudoalteromonas
Vibrio
Alteromonas
Shewanella
Salmonella
Xanthomonas
Burkholderia
Staphylococcus
Streptomyces
SW8
SW4
SW0
SWO8
SWO4
SWOB8
SWOB4
D
SW
SWO
SWOB
3
2
1
0
-1
-2
-3
Hyphomonas
Parvibaculum
Algibacter
Olleya
Winogradskyella
Formosa
Cellulophaga
Lacinutrix
Maribacter
Flavobacterium
Polaribacter
Tenacibaculum
Colwellia
Phaeobacter
Sulfitobacter
Celeribacter
Sphingorhabdus
Cycloclasticus
Klebsiella
Marinobacter
Marinomonas
Pseudomonas
Aequorivita
Ralstonia
Halomonas
Paraglaciecola
Bradyrhizobium
Rhizobium
Mesorhizobium
Sphingomonas
Sphingobium
Ca. Pelagibacter
Ca. Thioglobus
Zhongshania
Neptunomonas
Microbulbifer
Aeromonas
Vibrio
Alteromonas
Acinetobacter
Legionella
Paenibacillus
Staphylococcus
Streptomyces
Burkholderia
Xanthomonas
Shewanella
Salmonella
Pseudoalteromonas
Haemophilus
SW8
SW4
SW0
SWO8
SWO4
SWOB8
SWOB4

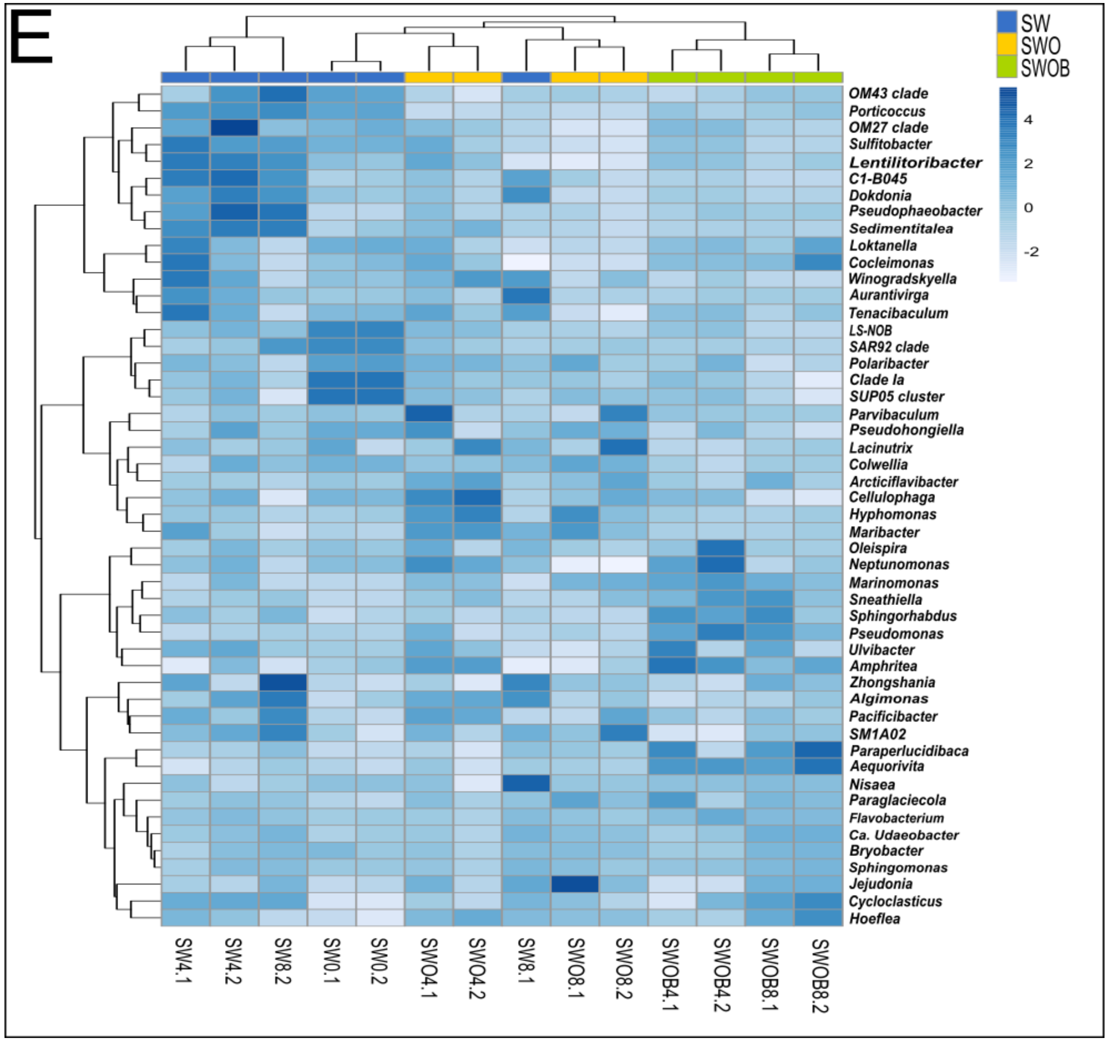

Fig. S3. The clustering of seawater (SW), oil contaminated seawater (SWO) and biostimulated oil contaminated seawater (SWOB) microcosms based on the crl-transformed proportions of the 50 predominant genera of Kaiju (A), Kaiju/MAR (B), Kraken2 (C), Bracken (D), and Amplicon (E) taxonomic classifications. The numbers in sample codes denote time in months, second number in sample code on subplot E denotes the treatment parallel number. The color intensity corresponds to crl-transformed proportion values with dark blue denoting genera with high proportion values in bacterial community.

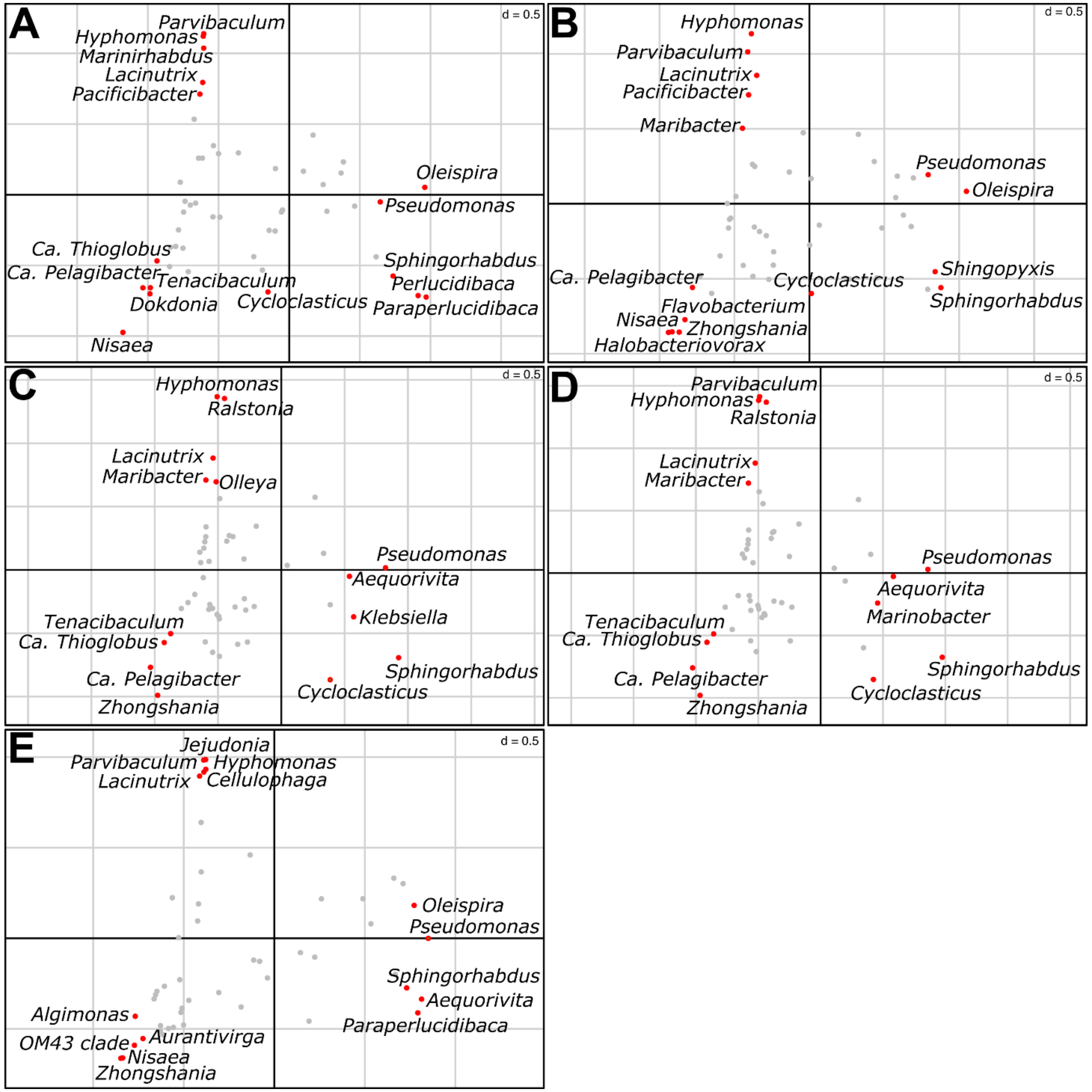

Fig. S4. Multiple co-inertia analysis results based on datasets of the top 50 bacterial genera proportions based on five taxonomic classification methods. Shown are variable spaces for each dataset (Kaiju with the NCBI-nr database (A), Kaiju with the MAR$_{DB}$ database (B), Kraken2 (C) and Bracken (D) with the Standard Kraken2 database, and Amplicon based sequencing with the SILVA database (E)).

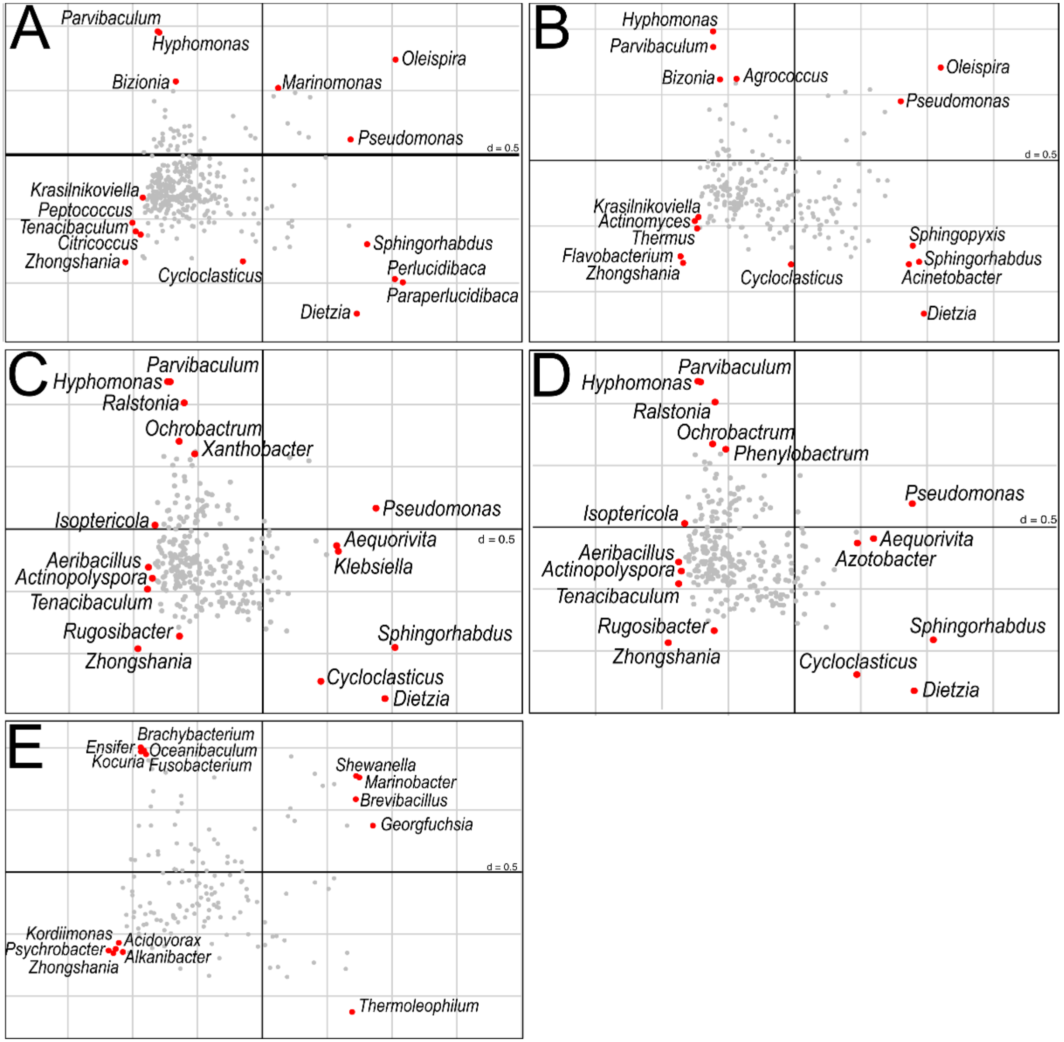

Fig. S5. Multiple co-inertia analysis results based on datasets of proportions of bacterial genera containing oil hydrocarbon degraders based on five taxonomic classification methods. Shown are variable spaces for each data set (Kaiju with the NCBI-nr database (A), Kaiju with the MAR$_{DB}$ database (B), Kraken2 (C) and Bracken (D) with the Standard Kraken2 database, and Amplicon based sequencing with the SILVA database (E)).

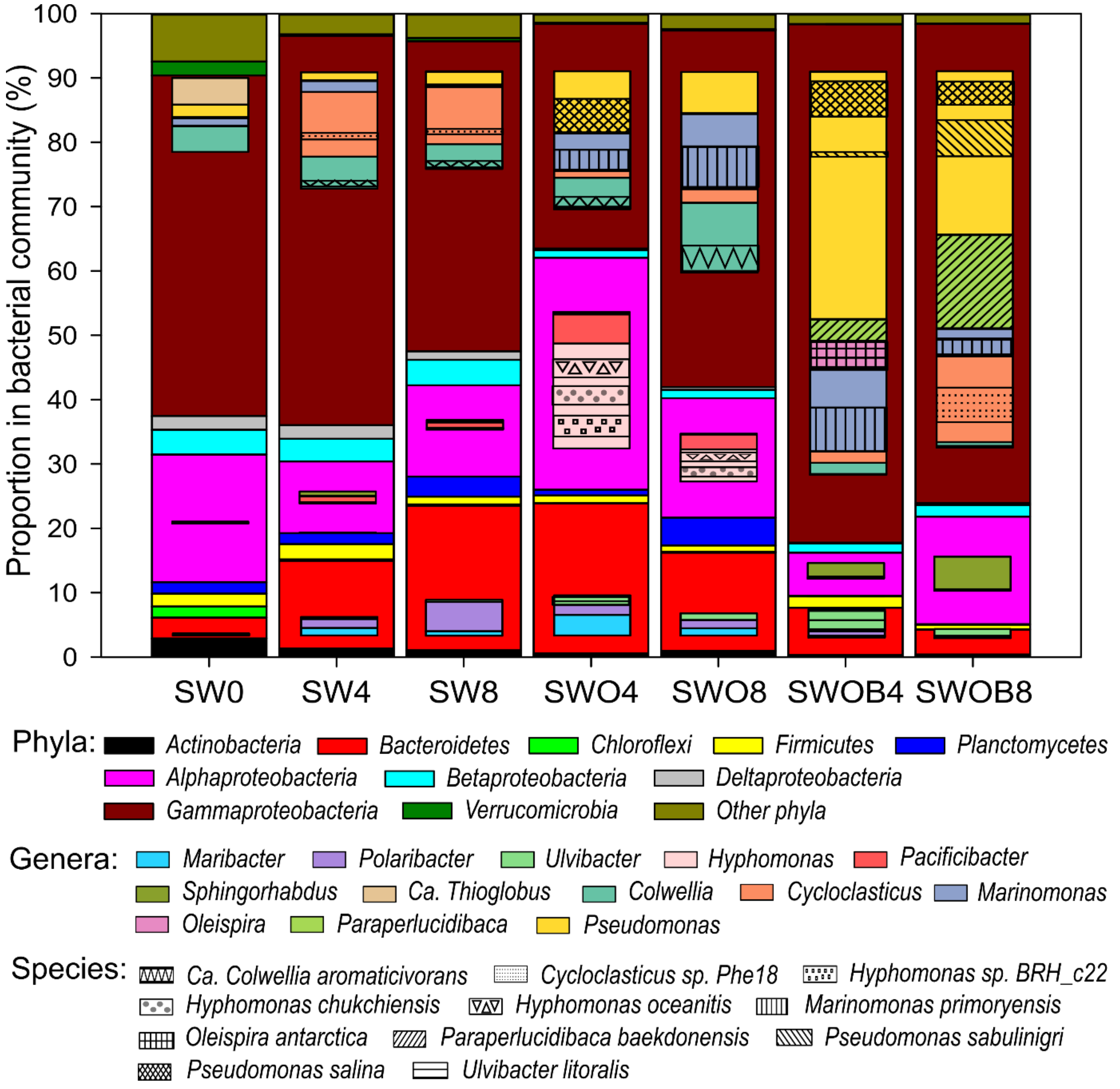

Fig. S6. The bacterial community structure at phylum, genus (>3%), and species (>3%) level in seawater (SW), oil-contaminated seawater (SWO), and biostimulated oil-contaminated seawater (SWOB). The numbers in sample codes denote time in months. Proteobacteria are presented at class level.

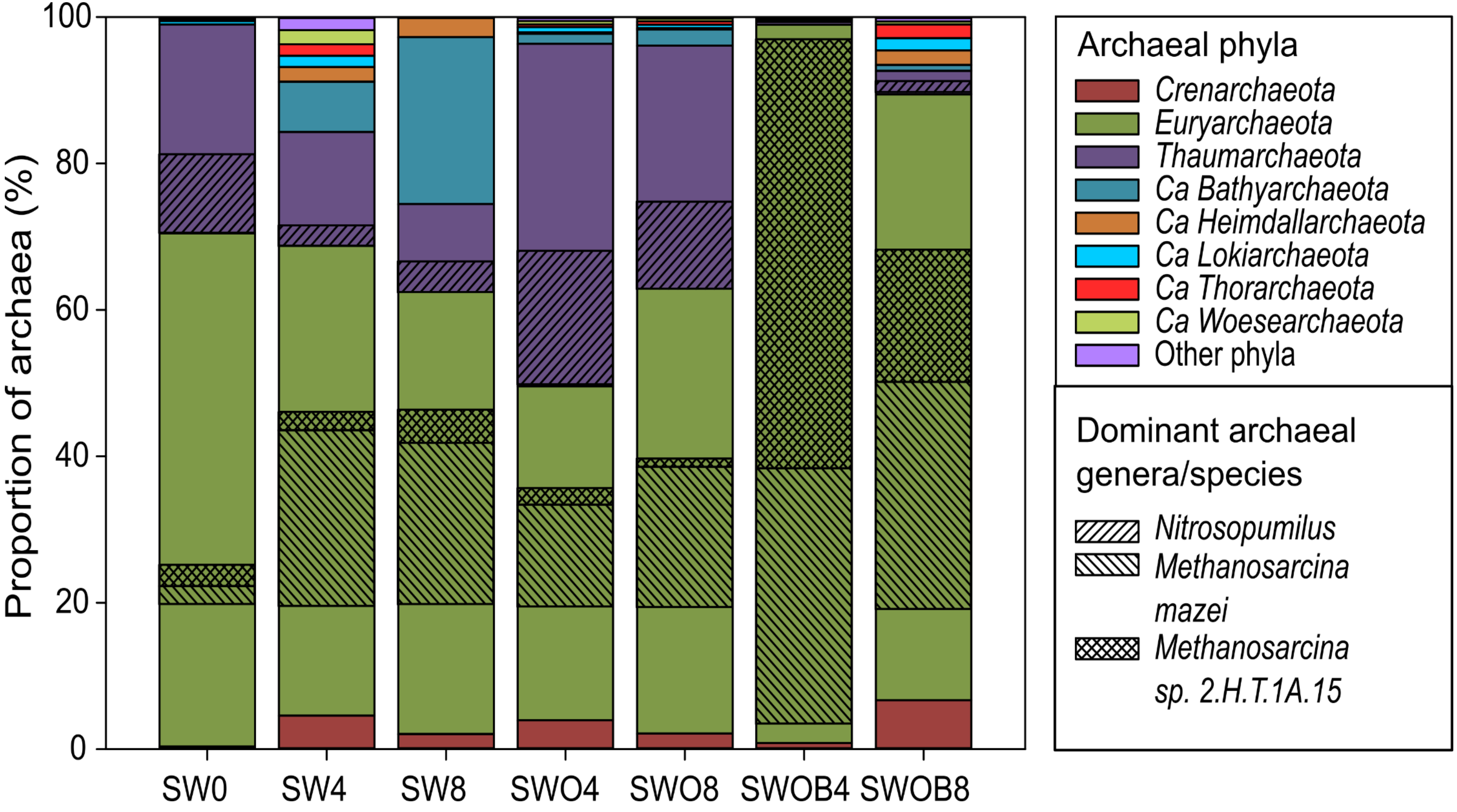

Fig. S7. The proportions of archaeal phyla as well as the most dominant archaeal genera and species in the archaeal community in seawater (SW), oil contaminated seawater (SWO) and biostimulated oil contaminated seawater (SWOB) according to taxonomic classification using Kaiju with the NCBI-nr database. The numbers in sample codes denote time in months.

Section S1: Prokaryotic Community Structure in Different Treatments According to Kaiju

Since Kaiju with the NCBI-nr database generally resulted in the highest number of classified reads (Table S3), the closest rank estimate to the average of all methods on the phylum level (Figure 3), and the highest overlap with other methods when considering the list of 50 most abundant bacterial genera (Figure 4), the prokaryotic community structure in different treatments of the current study is described according to this method.

At the phylum level, the bacterial community in the initial seawater (SW0), used to set up the treatment microcosms, was dominated by Proteobacteria (especially Gamma- and Alphaproteobacteria), which accounted for 79% of the community (Figure S6). Gammaproteobacterial genera Ca. *Thioglobus* and *Colwellia* (4.1% and 4.0%, respectively) were the predominant genera in SW0. In SW, the Bacteroidetes proportion increased over time, from 3.2% in SW0 to 22.5% of the bacterial community in SW8; this increase was mostly at the expense of Alphaproteobacteria in the first half of the experiment and also Gammaproteobacteria during the second half. In this treatment, the proportion of *Colwellia* was quite stable (3.9–4.9%) throughout the experiment, while *Cycloclasticus* became the most predominant genus (8.8–10%) from the fourth month onward.

The addition of Troll B type crude oil markedly changed the seawater bacterial community structure. By the fourth month, the proportions of Gamma-, Beta-, and Deltaproteobacteria, as well as those of less abundant phyla Actinobacteria, Planctomycetes, Chloroflexi, and Verrucomicrobia, were markedly diminished. In SWO4, the proportion of Alphaproteobacteria was almost doubled, and the proportion of Bacteroidetes increased 7-fold compared to SW0. The Alphaproteobacterial genus *Hyphomonas* dominated in SWO4 (16.3%), with four predominant species (Table S18), followed by Gammaproteobacterial genera *Pseudomonas* (especially the species *P. salina*) and *Marinomonas* (9.6% and 5.8%, respectively). At the phylum level, the bacterial community structure in SWO8 became quite similar to that in SW8, but differences were evident at the genus level. In SWO8, *Marinomonas* (especially *M. primoryensis*) and *Colwellia* (especially Ca. *C. aromaticivorans*) replaced *Hyphomonas* in the dominant position (11.7%, 10.9%, and 5.0% of the bacterial community, respectively).

In the SWOB treatment, the proportion of Gammaproteobacteria was increased at the expense of all other phyla (except Bacteroidetes) and Proteobacterial classes compared to SW0. Gammaproteobacteria formed 75–81% of the bacterial community throughout the experiment. *Pseudomonas* strongly dominated in SWOB4 (38%), followed by *Marinomonas*, especially *M. primoryensis* (6.8%), and *Oleispira*, especially *O. antarctica* (4.4%) (Table S18). The proportion of the second most abundant taxon, Alphaproteobacteria, rose from 6.8% in SWOB4 to 17% in SWOB8, largely owing to the increase in the genus *Sphingorhabdus* (5.1%). Although still dominant, the proportions of *Pseudomonas* and *Marinomonas* (25.4% and 4.3%, respectively) decreased over time, and *Paraperlucidibaca* (especially *P. baekdonensis* (14.6%)) and *Cycloclasticus* (13.4%) emerged among community dominants in SWOB8.

Roughly 2/3 of the archaeal community in SW0 was formed by Euryarchaeota, while Thaumarchaeota accounted for 1/3 (Figure S7). In SW, the proportion of Thaumarchaeota decreased over time, and Ca. Lokiarchaeota emerged as the second most dominant phylum by the eighth month. In SWO, the proportion of Thaumarchaeota and especially its genus *Nitrosopumilus* initially increased at the expense of Euryarchaeota, while the community structure at the phylum level reverted to that in initial seawater (SW0) by the eighth month. In the SWOB treatment, on the other hand, two Euryarchaeotal *Methanosarcina* species (*M. mazei* and *M. sp. 2HT1A15*) became highly dominant, forming 90% and 45% of the archaeal community in SWOB4 and SWOB8, respectively.

References

1. Liu, Z.; Lozupone, C.; Hamady, M.; Bushman, F.D.; Knight, R. Short pyrosequencing reads suffice for accurate microbial community analysis. *Nucleic Acids Res*. **2007**, *35*(18), e120, doi:10.1093/nar/gkm541.
2. Dethlefsen, L.; Huse, S.; Sogin, M.L.; Relman, D.A. The pervasive effects of an antibiotic on the human gut microbiota. as revealed by deep 16S rRNA sequencing. *PloS biol*. **2008**, *6*(11), 2383–2400, doi:10.1371/journal.pbio.0060280.
3. Espenberg, M.; Truu, M.; Truu, J.; Maddison, M.; Nõlvak, H.; Järveoja, J.; Mander, Ü. Impact of reed canary grass cultivation and mineral fertilisation on the microbial abundance and genetic potential for methane production in residual peat of an abondoned peat extraction area. *Plos ONE* **2016**, *11*(9), e0163864, doi:10.1371/journal.pone.0163864.
4. Krolicka, A.; Boccadoro, C.; Maeland, M.; Preston, C.M.; Birch, J.; Scholin, C.; Baussant, T. Detection of oil leaks by quantifying hydrocarbonoclastic bacteria in cold marine environments using the environmental sample processor. In *Proceedings of the 37th AMOP Technical Seminar on Environmental Contamination and Response*, Environment Canada, **2014**, pp. 791–807.
5. Gutierrez, T.; Biddle, J.F.; Teske, A.; Aitken, M.D. Cultivation dependent and cultivation independent characterization of hydrocarbon-degrading bacteria in Guaymas Basin sediments. *Front. Microbiol*. **2015**, *6*, 695, doi:10.3389/fmicb.2015.00695.
6. Widmer, F., Seidler, R.J., Gillevet, P.M., Watrud, L.S., Di Giovanni, G.D. (1998). A highly selective PCR protocol for detecting 16S rRNA genes of the genus Pseudomonas (sensu stricto) in environmental samples. *Appl. Environ. Microbiol*. **1998**, *64*(7), 2545-2553, doi:10.1128/aem.64.7.2545-2553.1998.
